# Synthesis of prebiotic organics from CO_2_ by catalysis with meteoritic and volcanic particles

**DOI:** 10.1038/s41598-023-33741-8

**Published:** 2023-05-25

**Authors:** Sophia Peters, Dmitry A. Semenov, Rupert Hochleitner, Oliver Trapp

**Affiliations:** 1 Department of Chemistry, Ludwig-Maximilians-Universität München, Butenandtstr. 5-13, 81377 Munich, Germany; 2 Max Planck Institute for Astronomy, Königstuhl 17, 69117 Heidelberg, Germany; 3 Mineralogische Staatssammlung München, Theresienstr. 41, 80333 Munich, Germany

**Keywords:** Origin of life, Asteroids, comets and Kuiper belt, Atmospheric chemistry, Early solar system, Meteoritics

## Abstract

The emergence of prebiotic organics was a mandatory step toward the origin of life. The significance of the exogenous delivery versus the in-situ synthesis from atmospheric gases is still under debate. We experimentally demonstrate that iron-rich meteoritic and volcanic particles activate and catalyse the fixation of CO_2_, yielding the key precursors of life-building blocks. This catalysis is robust and produces selectively aldehydes, alcohols, and hydrocarbons, independent of the redox state of the environment. It is facilitated by common minerals and tolerates a broad range of the early planetary conditions (150–300 °C, ≲ 10–50 bar, wet or dry climate). We find that up to 6 × 10^8^ kg/year of prebiotic organics could have been synthesized by this planetary-scale process from the atmospheric CO_2_ on Hadean Earth.

## Introduction

The formation of reactive organic molecules to form the building blocks of life on the nascent Earth is one of the prerequisites for abiogenesis^1–3^. The emergence of a stable continental crust and liquid water on the Earth at *∼* 4*.*4 Gyr ago^4,5^, and the earliest biogenic carbon isotope signatures at *∼* 3*.*8*–*4*.*1 Gyr ago^6,7^ suggest that life originated only ∼ 400–700 million years after the formation of the Earth^8–10^. This relatively short time span indicates that the major part of organic precursors has been already formed on the Hadean Earth as early as 4.4 Gyr ago. One possibility is that the prebiotic organic constituents that had been formed in the solar nebula, carbon-rich asteroids, and comets have been delivered onto the early Earth^11–21^. Other theories consider the synthesis in the atmosphere and in the ocean by catalytic or high energy processes (lightnings, volcanic energy, impact shocks)^22–64^. Some of the underlying chemical processes have been experimentally simulated, such as Miller-Urey synthesis in the CH_4_ rich atmosphere, Fischer–Tropsch^64^—catalysis in the CO rich environments, the CO_2_ fixation to pyruvate over iron and nickel nanoparticles^65^, the aqueous Strecker synthesis of amino acids inside carbonaceous asteroids, or the interstellar ice chemistry in comets.

Yet, it is difficult to deduce with certainty which scenario was the most dominant (if any) due to missing key data. In the exogeneous delivery scenario, besides debated early bombardment rates, a poorly known fraction of the organic matter would have been lost upon the atmospheric entry by ablation, due to impact shocks and pyrolysis by heating, or dissolution in the melted crust. In the insitu synthesis scenario, the Miller-Urey-like synthesis would have been less productive in a CO_2_- and N_2_-rich, neutral atmosphere of the early Earth compared to the reduced, CH_4_-, N_2_-, H_2_-rich atmospheric conditions assumed in the early experiments^66–68^.

Another possible scenario, the organic synthesis around hydrothermal vents at the ocean floor has been extensively studied^58,61,69,70^. The organic synthesis in the ocean has limitations related to dilution of the reaction products or vaporization by giant impacts^71^. There are other scenarios proposed e.g., organic synthesis in the Darwinian ponds on the continental surface^72^*,* synthesis driven by the native iron reduction of CO_2_^61^, or the Urey-Miller-like synthesis driven by converting a neutral Earth atmosphere to a reduced state upon collision with a single 10^23^ kg iron core^73^, etc.

**Figure 1:**
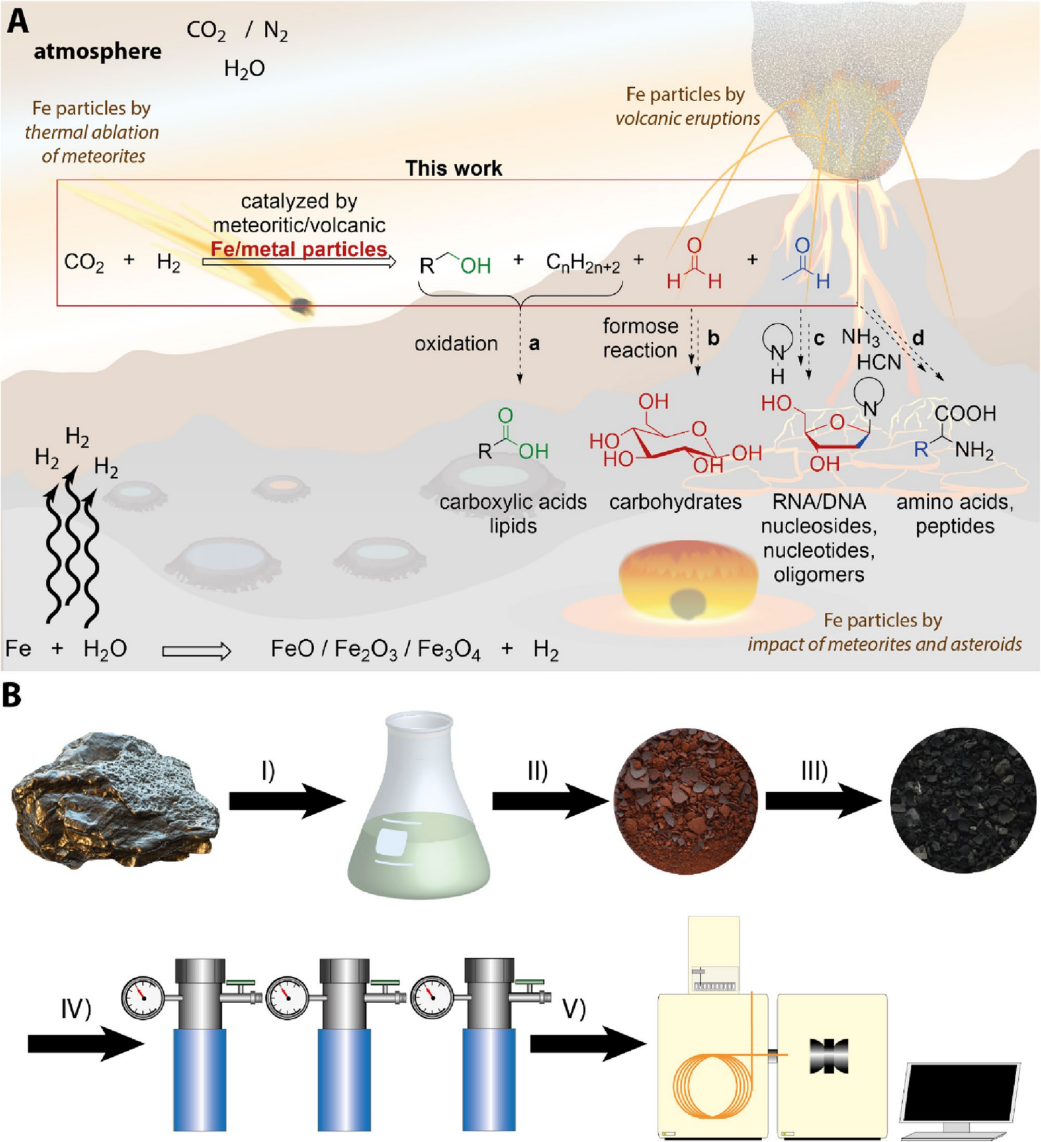
Formation of prebiotic key organic matter from CO_2_ by catalysis with meteoritic and volcanic particles. (**A**) Early Earth scenario with sources of catalytically active iron, and iron-rich particles. The exogenous sources include iron and iron-containing stony meteorites and asteroids producing nanoparticles by their thermal ablation in the atmosphere or after giant impacts. In situ sources are active volcanic chains similar to Hawaii, which produce iron-rich volcanic ash particles. These nano- and microscopic particles of elemental iron show catalytic activity and drive a robust synthesis of the feedstock atmospheric CO_2_ and H_2_ or H_2_O into key prebiotic organic compounds, at temperatures and pressures representative on the early Earth. Alternatively, H_2_ can be formed during the oxidation of elemental iron with water. These prebiotic organic compounds can act as reactants in further organic syntheses leading to the formation of carbohydrates, lipids, sugars^23^, amino acids^55^, and RNA and DNA molecules^35^. (**B**) Catalyst particles were prepared by acidic dissolution of iron meteorites *Campo del Cielo* and *Muonionalusta,* stone meteorite *Gao-Guenie* and volcanic ash from Etna (Sicily, Italy) (I), followed by the impregnation of support material, calcination at 450 °C (II), and reduction (III). To simulate a giant impact or volcanic eruption these materials were also ground in a ball mill. These catalytic particles were investigated in high-pressure autoclave experiments applying a broad range of conditions (9–45 bar and 150–300 °C) with a mixture of CO_2_ and H_2_ (IV). The reaction products were identified and quantified by GC–MS measurements (V).

We propose another robust pathway to the formation of key prebiotic organic matter on the early Earth. In this scenario, microscopic iron-rich particles from space or formed in situ by the giant impacts^74^, ablation of meteorites^75^, or produced by volcanic eruptions had been catalytically driving the CO_2_ fixation from the atmosphere. Natural minerals available on volcanic islands on the early Earth would have served as support materials in this synthesis (cf. Fig. 1). To test this idea, we investigated experimentally the catalytic properties of iron particles from meteorites and volcanic ash under the simulated reaction conditions of the early Earth in presence of CO_2_, H_2_ and H_2_O.

### Preparation of catalysts from meteorites and volcanic ash

As catalytically active metal sources on the early Earth we considered iron meteorites, stony meteorites, and volcanic ash. In our experiments we investigated representative samples of the iron meteorites Campo del Cielo and Muonionalusta, the stony meteorite Gao-Guenie, and volcanic ash from Etna. Their different metal composition is summarized in Table 1.

**Table 1:** Summarized composition of the metal precursors: name, mass percent of silicates or silicon (Si, %_mass_), carbon (C, %_mass_), iron (Fe, %_mass_), nickel (Ni, %_mass_), cobalt (Co, %_mass_), phosphorus (P, %_mass_), calcium (Ca, %_mass_), potassium (K, %_mass_), magnesium (Mg, %_mass_), sodium (Na, %_mass_), gallium (Ga, ppm), germanium (Ge, ppm), and iridium (Ir, ppm).

Name	Si	C	Fe	Ni	Co	P	Ca	K	Mg	Na	Ga	Ge	Ir
	[%_mass_]										[ppm]		
*Campo del Cielo*	–	–	92.6	6.7	0.4	0.3	–	–	–	–	87	407	3.6
*Muoniona-lusta*	–	–	91.3	8.7	–	–	–	–	–		0.3	0.1	1.6
*Gao-Guenie*^c^	76−70^b^	–	24–30	~ 1	–	–	–	–	–		–	–	–
Volcanic ash^a^	17.6	–	12.0	10^−3^	4·10 ^−3^	0.9	4.7	1.7	1.9	1.6	5·10^3^	10^3^	3·10^3^

^a^Measured by ICP.

^b^The complete mass of the silicates is given here.

^c^General composition of H5 chondrites.

The iron meteorites *Campo del Cielo* and *Muonionalusta* contain considerable amounts of Ni and traces of Ir, which are good hydrogenation catalysts.

It is well known that the catalyst particles stabilized on the surface enlarging supports (e.g. Al_2_O_3_) increase the catalytic activity and stability. Here, we follow a similar approach by combining our iron sources with natural minerals that might have been available on the early Earth. This simulates experimentally a process where the formed metal particles were deposited on minerals, e.g. (1) montmorillonite, (2) olivine, (3) diopside, and (4) hydroxyapatite. All of them are present either in the Earth crust or in meteorites and asteroids: While montmorillonite and hydroxyapatite can be formed under hydrothermal conditions, olivine and diopside are products of mafic volcanism. In addition, we use silica gel (5) as a reference system. The elemental compositions of these supports were determined via the energy dispersive X-ray analysis (EDX) and are summarized in Table 2.

**Table 2:** Summarized composition of the mineral supports determined by scanning electron microscopy (SEM).

Name	Sum formula	O	Mg	Si	Ca	Fe	P	Na	Al
		[%_atom_]							
Silica gel	SiO_2_	66.5	–	33.5	–	–	–	–	–
Montmorillonite	(Na,Ca)_0.33_(Al,Mg)_2_ Si_4_O_10_(OH)_2_	63.6	1.41	22.8	0.8	1.2	–	1.3	8.8
Olivine	(Mg,Fe)_2_[SiO_4_]	57.2	26.1	14.2	–	2.4	–	–	–
Diopside	MgCaSi_2_O_6_	60.2	9.8	20.1	9.9	–	–	–	–
Hydroxy apatite	Ca_5_[OH|(PO_4_)_3_]	64.3	−	–	22.1	–	13.6	–	–

The meteorites and the volcanic ash were dissolved in aqueous nitric acid yielding the stock solutions. The stone meteorite and the volcanic ash are not completely dissolved under these conditions, therefore the solutions were dispersed and used without filtration. For the preparation of the supported oxidized catalysts, the support (silica gel, hydroxyapatite, olivine, diopside and montmorillonite clay) was impregnated with the stock solution. The prepared suspension was dried at 100 °C and subsequently calcined at 450 °C for 4.5 h. Under these conditions the metal nitrates completely decompose under formation of the corresponding metal oxides.

For preparation of the ball milled oxidized catalysts from the *Campo del Cielo* meteorite, the meteorite was dissolved in aqueous nitric acid vide supra. This solution was dried at 100 °C. The resulting powder or volcanic ash was mixed with the support materials (silica gel, hydroxyapatite, olivine, diopside and montmorillonite clay) and milled in the ball mill at 800 rpm for 15 min.

**Figure 2:**
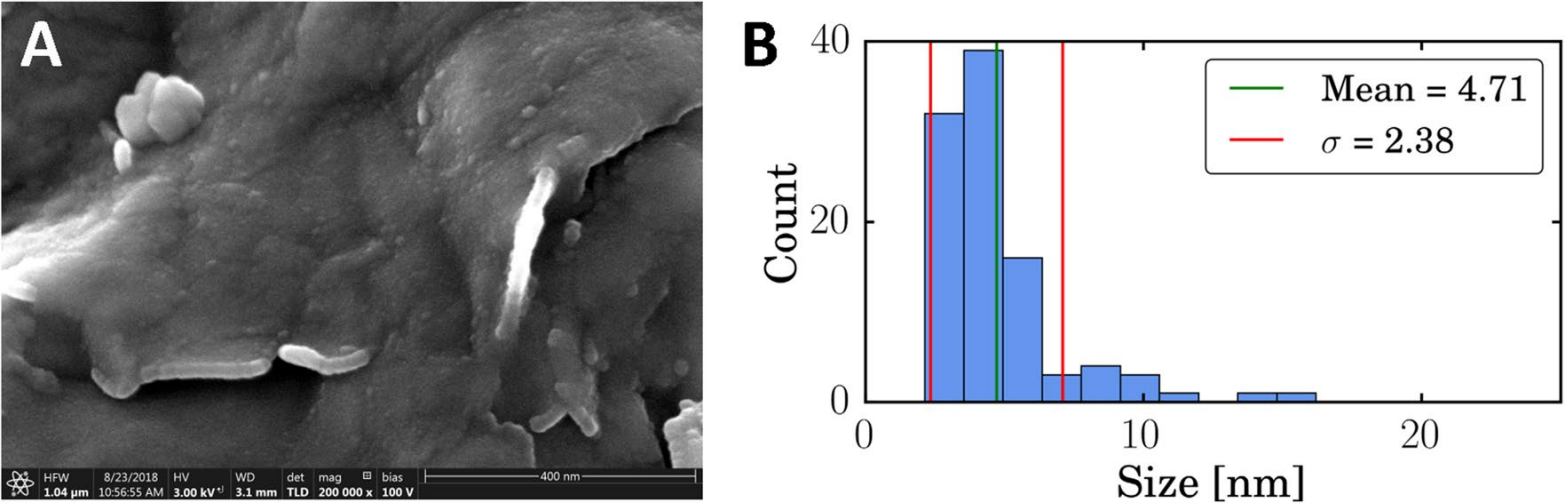
The pre-catalyst prepared from the *Campo del Cielo* meteorite supported on Montmorillonite. (**A**) Scanning electron microscopy (SEM) picture of oxidized particles of the Campo del Cielo meteorite on the support montmorillonite. (**B**) Size distribution of the nanoparticles with mean and standard deviation (σ).

This procedure simulates the crushing process of meteorites and asteroids or the formation of the volcanic ash particles. The freshly obtained catalysts were examined by the scanning electron microscopy (SEM) to determine their surface topology and the size of the formed nanoparticles. In Fig. 2 an SEM picture and the size distribution of the particles prepared by dissolution of a sample of the *Campo del Cielo* meteorite and impregnation on the montmorillonite support is shown. The mean size and narrow distribution of 4.7 ± 2.4 nm shows that small nanoparticles can be obtained, which can act as highly active catalysts. All samples were characterized by the SEM and the size distributions determined (see Supplementary Information section IV).

For the reduction of the oxidized catalysts, ≈ 1 g of the impregnated support materials was transferred in the glass insert (quartz glass) of the autoclave. The autoclave was evacuated and flushed with nitrogen (three times). After evacuation (9 × 10^–3^ mbar), hydrogen (≈ 50 bar) was pressurized into the autoclave. Reduction of the oxidized catalysts was achieved by heating to 300 °C for 17  hours.

### Screening of the reaction range and the catalytic properties of the catalysts prepared from meteorites and volcanic ash

After reduction the excess hydrogen of the reduced and cooled down supported catalyst was released and carbon dioxide and, subsequently, hydrogen or water were added with a defined partial pressure or volume, respectively. The pressurized autoclave was then heated to the corresponding temperature. The detailed reaction conditions are listed in the Supplementary Information section VII. After the set reaction time, the autoclave was cooled to 0 °C (to condense the volatile compounds). In order to separate the formed organic compounds from the catalyst, a distillation was performed. The black, frozen catalyst was cooled to − 180 °C and transferred to the glass distillation apparatus. This apparatus was evacuated to 3 × 10^–2^ mbar. Then, the leg of the distillation apparatus with the catalyst was heated to 210–220 °C and the evaporated compounds were condensed into a flask cooled with liquid nitrogen (− 180 °C). After completion of the distillation process the apparatus was opened and, after a warming, the reaction products were collected by adding dichloromethane (3 × overall 0.3 mL or 0.5 mL).

To investigate the catalytic properties of the prepared catalysts, a reaction screening was performed using a high-pressure autoclave system. The mineral supported catalysts were pressurized with CO_2_ and H_2_ gases at various partial pressures (CO_2_: 5–40 bar; H_2_: 5*–*41 bar; total pressure: 9–45 bar) and heated (150 °C*–*300 °C) in the autoclave system to cover a broad range of conditions. Under these conditions, the oxidized catalysts, which are typically red coloured by the oxidized Fe, are reduced and activated, turning to dark black coloured materials. The reduced material corresponds to the deposited particles formed by thermal ablation upon an entry of meteorites in the atmosphere or crushing of material by (geo-) mechanical forces. These autoclave reactions were performed for several days and weeks. After cooling down and release of pressure, the reaction products were isolated by separation from the catalysts by micro-distillation, and then identified and quantified using gas chromatography–mass spectrometry (GC–MS) (see Supplementary Information section V).

**Figure 3:**
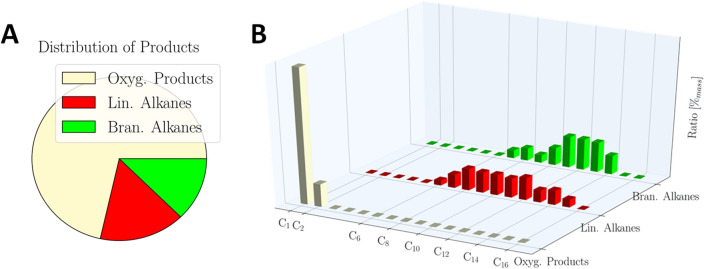
Reaction product distribution by the carbon number. Reaction conditions: a gas mixture of CO_2_:H_2_ = 1:2, a total pressure of 45 bar, temperature of 300 °C, and reaction time of 4 days. Catalyst: Metal particles prepared from the *Campo del Cielo* meteorite on montmorillonite as support. The overall yield was 934 µg and turn over number was 3.97 g/(kg d). (**A**) Pie chart of the product distribution (in mass percentage) separating oxygenated reaction products (yellow; methanol, acetaldehyde, and ethanol), *n*-alkanes (red; *n*-hexane to *n*-hexadecane) and *iso*-alkanes (green; from *iso*-heptane to *iso*-pentadecane). (**B**) Bar plot showing the mass percentage of compounds with a different number of C-atoms in each group: oxygenated reaction products (yellow), *n*-alkanes (red) and *iso*-alkanes (green).

Figure 3 shows the product distribution resulting from experiments with the catalytic active particles obtained from the *Campo del Cielo* meteorite supported on montmorillonite at 300 °C, a H_2_:CO_2_ ratio of 2:1 and a total pressure of 45 bar. The total product mass was 934 μg, with the yield being comparable to the results under hydrothermal conditions^59^. The main components were methanol, ethanol, and acetaldehyde, summing up to 70wt% in total. The residue consists of *n*-alkanes (*n*-hexane to *n*-pentadecane) and *iso*-alkanes (*iso*-heptane to *iso*-tetradecane), each accounting for approx. 15% of the total product mass. We also detected formaldehyde under these conditions (see Supplementary Information section V-B). The identification of such large amounts of oxygenated organic compounds is an exciting result, because formaldehyde and acetaldehyde are important building blocks for the synthesis of carbohydrates, amino acids and for forming deoxyribonucleosides. This analysis was performed for catalytic screening reaction with all catalytic materials prepared (see Supplementary Information VII). Under these conditions no other reaction products were detected. The product ratios of the compounds, the overall yield, and the distributions of the detected reaction products of catalysis depend on the physical conditions. Surprisingly, even the reaction temperatures as low as 150 °C and the extended reaction times of 14 days yielded reaction products, which shows that the materials are catalytically active even under mild reaction conditions. Therefore, a comprehensive screening by varying these reaction conditions was performed (vide infra).

To exclude potential contamination due to the solvents and from the experimental setup itself, we conducted a series of blank measurements to validate the results. Additionally, we performed experiments with the pure supports and metal sources to quantify their catalytic activities as well as impurities under the same reaction conditions. In all blank experiments, the resulting masses of oxygenated products, *n*-alkanes, and *iso*-alkanes did not exceed 4 μg, which is significantly lower than, for example, the  934 μg that have been obtained in the experiment shown in Fig. 3.

To perform a quantitative comparison of all experiments, we standardized the results by calculating turnover numbers (TON) for each experiment. The TON is defined as the mass of the reaction product (${m}_{product}$) divided by the product of the mass of the catalyst (${m}_{metcat}$) and time ($t$):

1 $$TON= \frac{{m}_{product} [g]}{{m}_{metcat}[kg] \cdot t[d]}$$

The turnover number is independent of the size of the nanoparticles. The size of the nanoparticles is very similar for the all considered materials, which have the size distribution between ~ 5 and 10 nm.

### Comprehensive catalytic investigations

**Figure 4:**
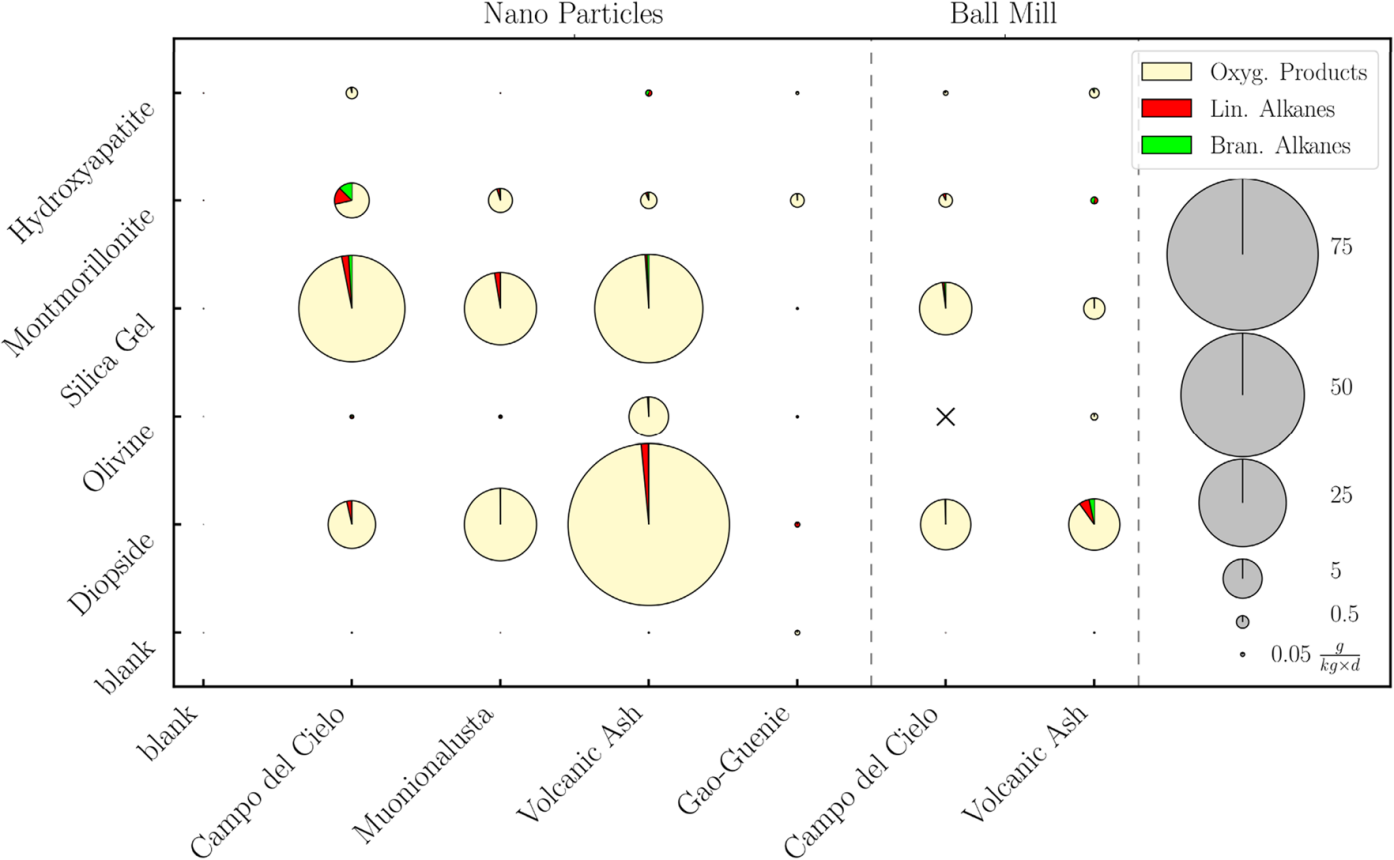
Screening of the metal sources and supports for the CO_2_ fixation. Metal sources (x-axis): blank (measurements without a supporter), Campo del Cielo (iron meteorite) as nanoparticles (Nanoparticles) and microscopic particles (Ball Mill), Muonionalusta (iron meteorite) as nanoparticles (Nanoparticles), Gao-Gunie (stone meteorite) as nanoparticles (Nanoparticles) and volcanic ash as nanoparticles (Nanoparticles) and microscopic particles (Ball Mill). Supports (y-axis): blank (without a metal source, ${m}_{metcat}$ is set to the mass of the support) silica gel, hydroxyapatite, olivine, montmorillonite, and diopside. A symbol "x" indicates that no reaction has occurred. The size of the circle reflects the turnover number of the catalyst (see scale at the right). The pie charts within these circles show the product distribution containing the oxygenated products (yellow: methanol, acetaldehyde and ethanol), *n*-alkanes (red: *n*-hexane to *n*-hexadecane) and *iso*-alkanes (green: from *iso*-heptane to iso-pentadecane). Reaction conditions: a gas mixture of CO_2_:H_2_ = 1:2, a total pressure of 45 bar, temperature of 300 °C, and reaction time of 4–5 days.

We investigated the catalytic materials prepared from the meteorite and volcanic ash samples systematically (reaction conditions: a gas mixture of CO_2_:H_2_ = 1:2, a total pressure of 45 bar, temperature of 300 °C, and reaction time of 4–5 days). Figure 4 summarizes the yields and the product distribution for all the catalysts on the various supports and prepared by the wet impregnation approach and by ball milling. The supports have a strong influence on the activity and can significantly enhance the catalytic activity [up to TON = 85.50 g/(kg d)]. This effect is smaller in the experiments with hydroxyapatite [up to TON = 1.00 g/(kg d)] and olivine [up to TON = 5.13 g/(kg d)]. In the case of hydroxyapatite, this is probably due to the alkaline surface, since an experiment with calcium carbonate showed similar results (see Supplementary Information). For olivine, it is likely that the surface properties influence the activity. In the same experiment with a synthetic forsterite (Mg_2_[SiO_4_]) that has a similar composition, but a different structure than the natural olivine, the activity is significantly increased [TON = 8.30 g/(kg d); see SI].

Comparing the metal sources, the volcanic ash shows the highest activity and, hence, has the highest turnover number. A reason for this observation could be the presence of catalytic promoters such as potassium and sodium salts, which were found in the volcanic ash (Table 1).

Iron and nickel rich *Muonionalusta* and *Campo del Cielo* meteoritic catalysts are slightly less active. The lowest turnover number is observed when using the stony meteorite as a source of catalytic particles. A possible reason could be the lower yield in catalytically active nanoparticles obtained in the preparation of these materials (see Tables 1 and 5).

The highest selectivity in favour of the formation of alkanes and branched alkanes was observed for the combination of metallic *Campo del Cielo* catalyst and montmorillonite (up to 30% alkanes) (cf. Fig. 3). In contrast to this, we found that all other catalysts showed higher selectivity for oxygenated products, which make up more than 95% of the total product mass.

We then studied the influence of the nanoparticle size on the catalytic activity. For this purpose, different metal salt concentrations of *Campo del Cielo* were mixed with silica gel or montmorillonite, dried, and calcined. By increasing the metal salt concentrations, the nanoparticles became smaller (silica gel: 13.38 nm > 11.81 nm > 9.63 nm) and we observed that the yield of CO_2_ [silica gel: TON = 1.37 g/(kg d) < 4.39 g/(kg d) < 36.97 g/(kg d)] conversion increased in catalytic experiments (for more details see Table S10 in the SI). Using a ball mill, we produced microscopic catalysts with a 100 times bigger particle size. Surprisingly, such large particles still showed catalytic activity. However, in contrast to the catalysts formed during wet impregnation, the metal ratio in these micrometer-sized catalysts had to exceed a threshold to show a significant product formation (see Supplementary Information Table S11). For the catalyst prepared from the *Campo del Cielo* meteorite supported on diopside, we observed a similar activity for ball milled micrometer-sized particles and for the nanoparticle catalysts. For the other catalysts, the turnover number for the ball mill particles was 3–24% of that of the nanoparticles (see Supplementary Information Table S11). This can be explained by the lower surface-to-volume ratio of the ball mill particles as compared to the nanoparticles, leading to a smaller number of catalytic surface sites per unit area of the support.

**Figure 5:**
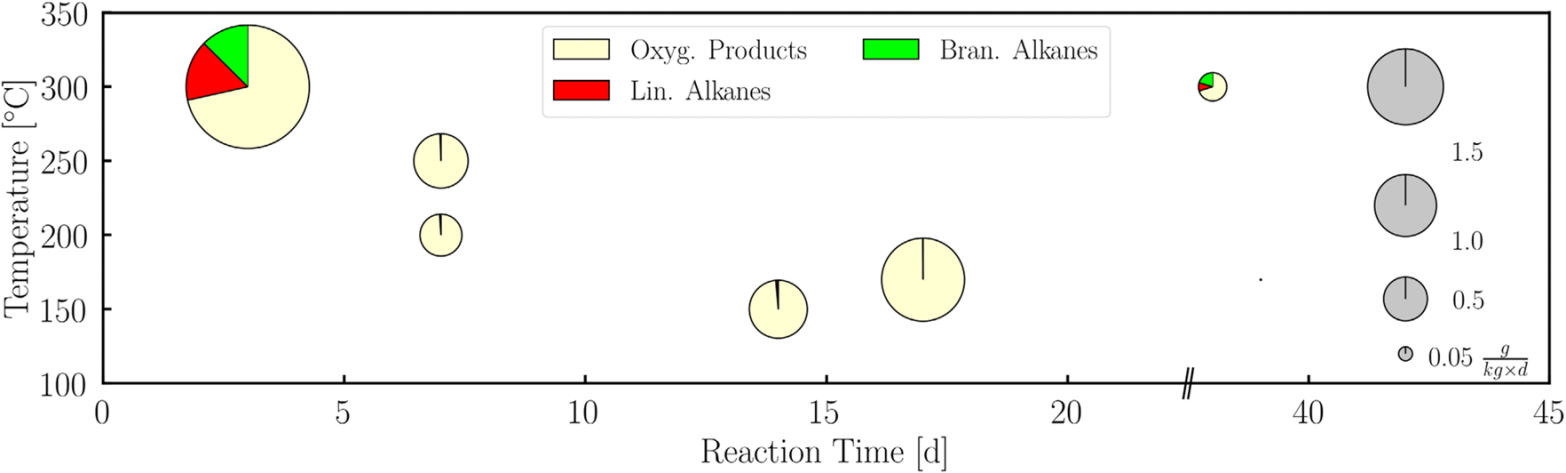
Temperature and reaction time dependence of the CO_2_ fixation. Turnover numbers at different reaction times (3–38 d) and temperatures (150–300 °C). The size of the circle reflects the turnover number (see scale at the right). The pie charts within these circles show the product distribution containing the oxygenated products (yellow: methanol, acetaldehyde and ethanol), *n*-alkanes (red: *n*-hexane to *n*-hexadecane) and *iso*-alkanes (green: from *iso*-heptane to *iso*-pentadecane). Reaction conditions: a gas mixture of CO_2_:H_2_ = 1:2 and a total pressure of 45 bar. Iron particles were prepared from the *Campo del Cielo* meteorite at montmorillonite as support.

**Figure 6:**
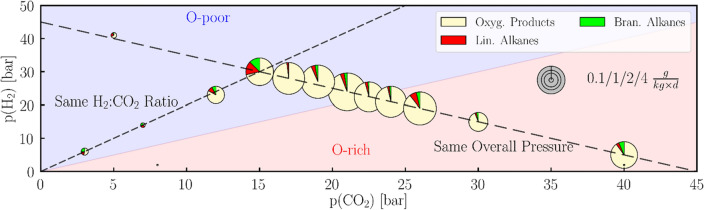
Partial pressure dependence of the CO_2_ fixation. Turnover number at different partial pressures of carbon dioxide (3–40 bar) and partial pressures of hydrogen (2–40 bar). The size of the circle reflects the turnover number (see scale at the right). The pie charts within these circles show the product distribution containing the oxygenated products (yellow: methanol, acetaldehyde and ethanol), *n*-alkanes (red: *n*-hexane to *n*-hexadecane) and *iso*-alkanes (green: from *iso*-heptane to *iso*-pentadecane). Reaction conditions: either a gas mixture of CO_2_:H_2_ with equal ratio 1:1 but varied total pressure or CO_2_:H_2_ with varied ratio but a total pressure of 45 bar. Temperature is 300 °C, and reaction time is 3–4 days. Iron particles prepared from the *Campo del Cielo* meteorite on montmorillonite as support.

We identified favourable environmental conditions for the formation of key prebiotic organic matter on the early Earth by systematic screening of the reaction conditions. The results for various temperatures as well as for various CO_2_:H_2_ ratios and pressures using the catalytic particles from the *Campo del Cielo* iron meteorite on the montmorillonite support are depicted in Figs. 5 and 6, respectively.

Figure 5 shows that there is a strong temperature influence on the reaction products. Below 300 °C, only oxygenated products were formed with the TON increasing with temperature and reaction time, while at 300 °C, both *n*-alkanes and *iso*-alkanes were detected. Here, the total mass of the products decreased with reaction time [38 d and 300 °C: TON = 0.21 g/(kg d)]. However, a larger variety of branched alkanes was observed at the end of the experiment after 38 days, likely formed by cracking alkanes and rearrangement reactions on the montmorillonite support^41^.

The most striking result is that the catalysis with meteoritic particles is robust and produces prebiotic organic matter under both neutral and reducing atmospheric conditions (see Fig. 6). The highest overall yield and the largest share of oxygenated products was observed at a CO_2_:H_2_ pressure ratio of 21:24 and a total pressure of 45 bar [TON = 7.3 g/(kg d)]. The overall catalytic activity was lower at lower total gas pressures, as commonly observed in the Fischer–Tropsch catalysis^76^. The catalysis is more efficient in a CO_2_-rich [CO_2_:H_2_ = 40:5; TON = 3.72 g/(kg d)] than in an H_2_-rich environment [CO_2_:H_2_ = 5:41; TON = 0.16 g/(kg d)]. Both the dependence on the temperature and the atmospheric conditions suggest that the oxygenated compounds such as aldehydes and alcohols are formed under milder conditions, while alkanes are formed by reactions of these intermediates at elevated temperatures.

As already pointed out, the most readily produced oxygenated products (especially the aldehydes) are essential compounds for the formation of building blocks of life such as (deoxy) ribosyl moiety of RNA and DNA nucleosides^35,36^, amino acids^55^, and carbohydrates^22,57^. If we project our experimental results to the history of the early Earth, the yield and the selectivity towards oxygenated products should have increased with the gradual loss of the primordial H_2_ and the cooling of the atmosphere.

**Figure 7:**
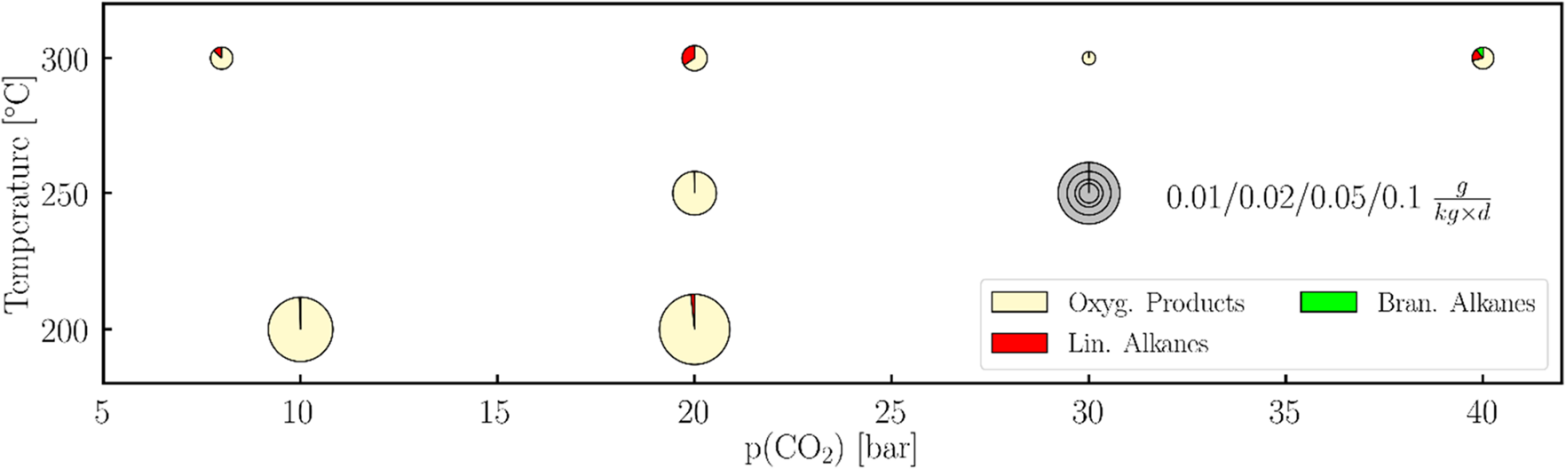
CO_2_ fixation with low hydrogen partial pressure. Turnover numbers at different partial pressures of carbon dioxide (8–40 bar) and temperatures (200–300 °C) are shown. The size of the circle reflects the turnover number (see scale at the right). The pie charts within these circles show the product distribution containing the oxygenated products (yellow: methanol, acetaldehyde and ethanol), *n*-alkanes (red: *n*-hexane to *n*-hexadecane) and *iso*-alkanes (green: from *iso*-heptane to *iso*-pentadecane). Reaction conditions: The partial pressures of hydrogen in these experiments were 1–2 bar and the reaction time was 3–10 days. Iron particles prepared from the *Campo del Cielo* meteorite supported on montmorillonite.

To consider environmental conditions under which H_2_ was not readily available, experiments have also been carried out under reduced partial pressure of H_2_ (see Fig. 7) and solely in the presence of CO_2_ and H_2_O (see Supplementary Information Table S14). In the former we can clearly see that the CO_2_ fixation still works under the mild conditions of 10 bar carbon dioxide, 1 bar hydrogen and 200 °C, although the turn-over number is reduced by two orders of magnitude compared to the experiments at higher pressures. These reaction conditions also reflect other models predicting lower atmospheric pressures on the Hadean earth, e.g. a maximum of ~10 bars of CO_2_ and ~1 bar of nitrogen^77^ or even lower pressures^78^. Again, we observe that alkanes are only formed at higher temperatures. In the experiments with water, we observed that the redox reaction of reduced iron with water led to the continuous in situ generation of H_2_^78^. This coupled process of the H_2_ formation and its immediate reaction with CO_2_ produces oxygenated products, as for the case of the CO_2_–H_2_ experiments.

### Mechanistic considerations

**Figure 8:**
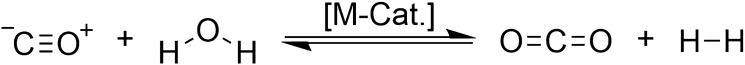
Water gas shift reaction, which could have a positive effect on the formation of alkanes in CO_2_ fixation.

Because alkanes were formed only under harsher reaction conditions and their yield is a few orders of magnitude lower than the yield of the oxygenated products, it is likely that two different, partially independent mechanisms are responsible for their production. The formation of the alkanes and the fact that they are only observed under certain conditions (montmorillonite, *Campo del Cielo*, high temperatures, high partial pressure of hydrogen) can be explained by the water gas shift reaction shown in Fig. 8.

The use of montmorillonite as a catalyst leads to the removal of water. Therefore, it shifts the equilibrium to the carbon monoxide side. The same holds when the partial pressure of carbon dioxide is low, and the partial pressure of hydrogen is high. The latter also leads to a very high concentration of hydrides on the surface, and thus a highly reductive environment. For the explanation of the effect of temperature, we have to look at the state of aggregation of water, because the presence of gaseous water leads to a shift of the equilibrium to the side of carbon monoxide. Starting from 250 °C and the resulting pressure of 89 bar in the autoclave, water is gaseous. Therefore, our conditions favour the presence of carbon monoxide which can form linear and branched alkanes via the classical Fischer–Tropsch reaction. Only *Campo del Cielo* shows a significant production of alkanes, since all other metal sources contain elements that hamper the Fischer–Tropsch reaction, for example, sulphur. Since the conditions favouring alkanes also shift the water gas reaction towards the side of carbon monoxide, it is assumed that they are formed by the classical Fischer–Tropsch reaction.

**Figure 9:**
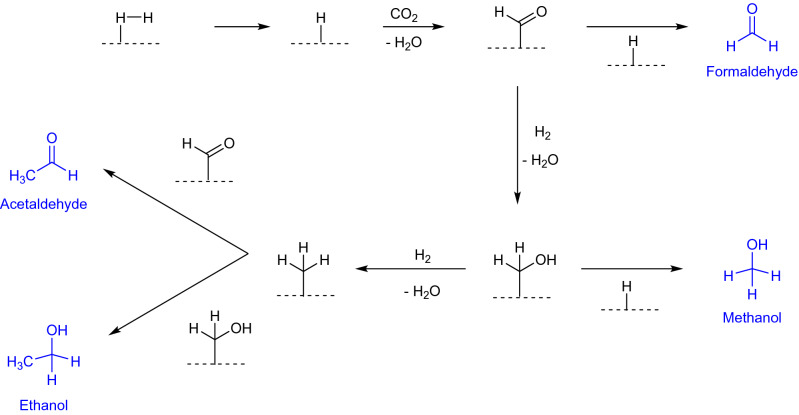
Proposed mechanism for the carbon fixation. As a first step, hydrogen bond is cleaved on the surface as in the Fischer–Tropsch and Haber–Bosch synthesis. By addition of carbon dioxide and condensation, a surface-formyl is formed, similar to the mechanism proposed by Varma, Muchowska, Chatelain, and Moran^61^. Next, via reduction and recombination of the surface-species, the products, which are marked blue, are formed. A dashed horizontal line shows the metal surface.

We propose a mechanism for the formation of the oxygenated products (see Fig. 9). This mechanism is also in agreement with the findings by Varma, Muchowska, Chatelain, and Moran for the formation of formic acid, methanol, and acetate under hydrothermal conditions^61^ and also observed for nanoparticle catalysts^79^. The first step is the dissociation of hydrogen on the catalytic surface, as known from the Haber–Bosch and the Fischer–Tropsch reaction. With addition of carbon dioxide, a surface-formyl is formed. This compound can be reduced to a surface-hydroxymethyl and a surface-methyl.

Recombination of these compounds with each other or with a surface-hydride leads to the formation of formaldehyde, methanol, acetaldehyde, and ethanol. This mechanism also suggests production of methane and ethane, which were however not detected under investigated reaction conditions. Please note that there is no possibility to build CH_2_-fragments to achieve a chain elongation (as in the Fischer–Tropsch reaction), and hence only products with a maximum of two carbon atoms are generated. The proposed mechanism additionally explains why, in contrast to hydrothermal^59,61^ and laser spark experiments^24,59^, no carboxylic acids were formed.

### Implications for the early Earth

The catalysed synthesis as a source of prebiotic organic matter should have been most active during Hadean, as it requires surface temperatures above about 150 °C and several bar of atmospheric pressure to operate. The conditions on the early Earth at that time, with high planetesimal bombardment rates and volcanic activity should have been the most favourable for the production of iron-rich catalytic particles and atmospheric H_2_, driving the synthesis. Following predictions of the atmospheric model for the early Earth with at least several to several tens of giant impacts during Hadean^80^, we considered a post-impact neutral CO_2_:H_2_:N_2_ atmosphere with a total pressure of about 3–10 bar and a surface temperature of the volcanically active islands of about 130–200 °C (for details, see Supplementary Information section VIII). Next, we assumed that montmorillonite-like minerals, a common product of the acidic weathering of volcanic rocks, covered the surface of these islands and served as support for this synthesis. The subaerial surface at that time was at most about 1% of the modern continental landmass. The minimum and maximum total delivery rates of the exogenous matter of ∼ 3 × 10^12^ and 10^15^ kg/yr were adopted^52^, and a mass fraction of FeNi and iron-rich compounds was calculated using measured meteoritic and IDP abundances. Assuming that only < 0.1 – 1% fraction of the metals or iron-rich silicates would recondense as catalytic nano- and micron-sized particles, the total delivery rate of the exogenous catalysts on the subaerial surface of the Hadean Earth could have been ∼ 5 × 10^7^–4 × 10^11^ kg/yr (Supplementary Information, section VIII.B).

We also assumed that the volcanic activity on the Hadean Earth has been at most several times higher than on the modern Earth, producing a total ash volume of up to ~ 20–100 km^3^ (Supplementary Information, section VIII.C). Based on modern geological data, about 4 wt% of that volume would be represented by the iron-rich silicate particles smaller than 30 micron. The resulting total deposition rate of the fine ash particles into the subaerial surface could have been ~ 2 × 10^11^–10^12^ kg/yr. Finally, based on our experimental results, we assumed that the catalytic particles deposited on minerals would be active for about 30 days.

By taking the measured synthesis yields at *T* = 150–200 °C and scaling these values down to the total pressures of ~ 3–7 bar, the resulting production rate of the prebiotic organics in a CO_2_-rich atmosphere with *P*(CO_2_)/*P*(H_2_) ~ 1–2 could have been up to ~ 10^6^–6 × 10^8^ kg/yr (Supplementary Information, section VIII.D). This is comparable or exceeding the delivery rate of the exogenous organic matter onto the subaerial surface of ~ 2 × 10^7^ kg/yr and endogenous production in the post-impact zones of ~ 10^4^ kg/yr^44^. The Urey-Miller-like organic synthesis driven by volcanic lightnings could have added another ≲4 × 10^9^ kg/yr of organics to this production rate^59^. In contrast, if the early Earth atmosphere had been CO-rich instead of CO_2_-rich^80,81^, the Fischer–Tropsch synthesis driven by the native iron or exogenous FeNi particles would produce up to 7 × 10^10^–5 × 10^13^ kg/yr of CH_4_, 7 × 10^9^–5 × 10^12^ kg/yr of HCN, and other organics^82,83^.

We conclude that the catalytic synthesis presented in this study could have produced a substantial amount of key oxygenated organics in a neutral, CO_2_-rich atmosphere on the Hadean Earth^77^, complementing production of precursors of sugars and amino acids by other processes^66,84,85^. Over a geologically short time span of several tens of Myr, the mass of prebiotic organics synthesized in situ on the early Earth could have been as high as ~ 10^13^–6 × 10^16^ kg, comparable with or exceeding the modern aquatic and total terrestrial biomasses, respectively^86^.

## Conclusions

In this work, we experimentally showed that meteoritic and volcanic iron-rich particles are efficient catalysts for converting atmospheric carbon dioxide into hydrocarbons, methanol, ethanol, formaldehyde, and acetaldehyde, which are important precursors for lipids, nucleosides, sugars, and amino acids. These particles exhibit catalytic activity in the presence of naturally occurring minerals and atmospheric CO_2_, H_2_ or H_2_O at temperatures and pressures representative of the early Earth. Since the early Earth’s atmosphere has likely been evolving toward a more oxidizing state, the oxygen-containing organic compounds would have been produced more efficiently with time, promoting further synthesis of more complex prebiotic compounds. Thus, the early Earth and similar young rocky exoplanets could be regarded as a giant catalytic reactor converting feedstock atmospheric gases into complex prebiotic organic matter.

## Materials and methods

Anhydrous dichloromethane (Honeywell or Acros Organics, 99.8%), iron nitrate nonahydrate (Alfa Aesar, 98.0–101.0%), nickel nitrate hexahydrate (Alfa Aesar, 98%), cobalt nitrate hexahydrate (Alfa Aesar, 98.0–101.0%), germanium tetrachloride (Alfa Aesar, 99.99999%), gallium nitrate (Sigma Aldrich, GA 9–10% W/W), dimedone (Sigma Aldrich, ≥ 99.0%), anhydrous iridium chloride (Strem Chemicals, 99.95+%) were purchased and used as received. Hydrogen (99.999%) and carbon dioxide (99.998% or 99.995%) were purchased from Air Liquid. Fused silica capillaries were purchased from MicroQuartz, Munich, Germany. The gas chromatographic stationary phase GE-SE-30 was obtained from Macherey & Nagel, Düren, Germany. Montmorillonite was purchased from Alfa Aesar as 'naturally occurring mineral', ~ 200 mesh powder and used as received. Silica gel was purchased from Acros Organics as 0.035–0.070 mm, 60 Å powder and used as received. *Campo del Cielo* was purchased from Decker Meteorite-Museum, Oberwesel, Germany, and used as received. The meteorites *Muonionalusta*, and *Gao-Guenie*, the minerals olivine and diopside were provided by the 'Mineralogische Staatssammlung Bayern'. The minerals were powdered in the ball mill (400 rpm for 15 min). The volcanic ash was collected from an eruption of the Etna, Sicily, Italy, on April 23rd 2012 in Fornazzo. For the reductions and reactions under high pressure, a high-pressure stainless-steel autoclave with a 200 mL glass insert with digital pressure gauges, fine throttling valve, and temperature sensor 330 mm. The autoclave is tightened with silver gasket. The temperature was adjusted by a heating hood 20 S, equipped with a magnetic stirrer. The autoclaves were purchased from Carl Roth, Karlsruhe, Germany. For reactions under aqueous conditions an addition a three-way ball valve was installed between the autoclave and the fine throttling valve. The autoclaves were pressurized with a home build high pressure screening setup^87^. The Planetary Ball Mill 33 Pulverisette 7 was purchased from Fritsch GmbH, Idar-Oberstein, Germany and was used with two grinding bowls of 20 mL stainless steel and 12 balls of 10 mm stainless steel. A Thermo Trace gas chromatograph (San Jose, California, USA) equipped with a split-injector (250 °C), a flame ionization detector (250 °C) for the quantitative analyses, and for the identification a quadrupole ion trap (PolarisQ MS) mass spectrometer or quadrupole (ISQ single quadrupole MS) mass spectrometer was used, respectively. Gas chromatographic analysis was performed on a 25 m GE-SE-30 250 nm (ID 250 μm).

### Catalysis with meteoritic and volcanic particle catalysts

For the catalyst activities we were used the catalysts in CO_2_ fixation under standard conditions (T = 300 °C, p = 45 bar, H_2_:CO_2_ = 2:1, t = 3–4). The results are summarized in Table 3.

**Table 3:** Results of the screening of nanoparticular catalysts with the metal source: Campo del Cielo (Cdc), volcanic ash (va), Muonionalusta (Mn), Guenie-Gao (Gg), the supports Diopsid, silica gel, montmorillonite (mont.), olivine, hydroxy apatite (hydroxy ap.), calcium carbonate (calcium carb.), synthesized olivine [olivine (syn)] and the metal concentration.

Catalyst			M (products)				TON			
Metal source	Mineral	m (Met.)	oxy. p	*n*-alk	*iso*-alk	Σ	oxy. p	*n*-alk	*iso*-alk	Σ
		[‰]	[μg]				$$\frac{{\text{g}}}{{{\text{kg}}\;{\text{d}}}}$$			
va	Diopsid	8.9	1588	23	2	1613	84.20	1.22	0.08	85.50
Mn	Diopsid	38.6	909	1	–	910	17.12	0.02	–	17.14
Cdc	Diopsid	73.3	2130	74	4	2208	7.13	0.25	0.01	7.39
Gg	Diopsid	10.6	–	3	–	3	–	0.08	–	0.08
Va	Silica gel	8.8	1066	6	6	1077	37.99	0.22	0.20	38.41
CdC	Silica gel	7.4	5365	118	55	5539	35.81	0.79	0.37	36.97
Mn	Silica gel	14.2	2852	77	–	2930	16.61	0.45	–	17.06
Gg	Silica gel	10.5	–	–	–	–	–	0.01	–	0.01
Cdc	mont.	26.1	682	152	120	953	2.84	0.63	0.50	2.97
Mn	mont.	26.2	266	10	4	280	1.78	0.07	0.02	1.88
va	mont.	6.1	13	1	–	14	0.81	0.04	0.02	0.87
Gg	mont.	13.2	17	–	–	18	0.60	–	–	0.60
va	Olivine	8.9	96	1	–	97	5.07	0.03	0.03	5.13
Cdc	Olivine	54.7	2	3	2	7	0.01	0.02	0.01	0.05
Mn	Olivine	32.3	1	1	–	2	0.01	0.02	0.01	0.04
Gg	Olivine	15.6	–	–	–	1	0.01	0.01	–	0.02
Cdc	Olivine (syn)	6.0	379	1	–	380	8.26	0.04	–	8.30
Cdc	Calcium carb.	5.0	58	–	–	58	0.28	–	–	0.28
Cdc	Hydroxy ap.	34.3	151	–	–	151	1.00	–	–	1.00
va	Hydroxy ap.	8.2	–	1	1	1	-	0.07	0.05	0.12
Gg	Hydroxy ap.	13.3	1	–	–	1	0.03	–	–	0.03
Mn	Hydroxy ap.	31.3	–	–	–	–	–	–	–	–

The masses of oxygenated products (oxy. p., in mg), n-alkanes (n-alk, in mg), iso-alkanes (iso-alk, in mg) and the total mass of all detected products (Σ in mg) as well as the turnover number (TON) of of oxygenated products (oxy p., in $\frac{{\text{g}}}{{{\text{kg}}\;{\text{d}}}}$), n-alkanes (n-alk, in $\frac{{\text{g}}}{{{\text{kg}}\;{\text{d}}}}$), iso-alkanes (iso-alk, in $\frac{{\text{g}}}{{{\text{kg}}\;{\text{d}}}}$) and the total TON of all detected products (Σ$\frac{{\text{g}}}{{{\text{kg}}\;{\text{d}}}}$).

To verify the size metal source particles, catalysts were prepared from montmorillonite and silica gel, respectively, with different metal concentrations of *Campo del Cielo*. The smaller the metal concentration, the smaller the nanoparticles. These catalysts, were now used in CO_2_ fixation under standard conditions (T = 300 °C, p = 45 bar, H_2_:CO_2_ = 2:1, t = 3–4). The results are summarized in Table 4.

**Table 4:** Results of the screening of nanoparticle size catalysts using the prepared materials: supports montmorillonite (mont.) and silica gel (sg), their metal concentration [m(metal) in %] and their resulting particle size (∅ in nm) and the masses of oxygenated products (oxy. p. in mg), n-alkanes (n-alk in mg), iso-alkanes (iso-alk in mg) and the total mass of all detected products (Σ, in mg) as well as the turnover number (TON) of oxygenated products (oxy. p. in g kg d), n-alkanes (n-alk in $\frac{{\text{g}}}{{{\text{kg}}\;{\text{d}}}}$), iso-alkanes (iso-alk in $\frac{{\text{g}}}{{{\text{kg}}\;{\text{d}}}}$) and the total TON of all detected products (Σ in $\frac{{\text{g}}}{{{\text{kg}}\;{\text{d}}}}$).

Catalyst			m(products)				TON			
Mineral	m(metal)	Θ size	oxy. p	*n*-alk	*iso*-alk	Σ	oxy. p	*n*-alk	*iso*-alk	Σ
	[%]	[nm]	[μg]				$$\frac{{\text{g}}}{{{\text{kg}}\;{\text{d}}}}$$			
mont.	22.79	9.21	1626	15	–	1642	1.68	0.02	–	1.69
mont.	11.02	6.97	1588	2	–	1590	4.32	0.01	–	4.33
mont.	7.39	5.50	682	152	120	953	2.84	0.63	0.50	3.97
sg	21.64	13.38	1424	14	–	1438	1.36	0.01	–	1.37
sg	12.55	11.81	2408	28	1	2437	4.24	0.05	–	4.39
sg	7.35	9.63	5365	118	55	5539	31.81	0.79	0.37	36.97

To verify the micrometer size, catalysts were prepared from all supports, with different metal concentrations of Campo del Cielo} and volcanic ash, respectively. These catalysts, were now used in CO_2_ fixation under standard conditions (T = 300 °C, p = 45 bar, H_2_:CO_2_ = 2:1, t = 3–4). The results are summarized in Table 5.

**Table 5:** Results of the screening of micrometer size catalysts using the prepared catalyst with the metal source Campo del Cielo (Cdc) and volcanic ash (va), the supports diopsid, olivine, silica gel montmorillonite (mont.), hydroxy apatite (hydroxy ap.) and the metal concentration [m(metal) in %].

Catalyst			m(products)				TON			
Metal source	Mineral	m(Met.)	oxy. p	*n*-alk	*iso*-alk	Σ	oxy. p	*n*-alk	*iso*-alk	Σ
		[‰]	[μg]				$$\frac{{\text{g}}}{{{\text{kg}}\;{\text{d}}}}$$			
Cdc	Diopsid	3.0	51	–	–	51	8.25	0.04	–	8.29
Cdc	Olivine	3.8	–	–	–	–	–	–	–	–
Cdc	Silica gel	9.0	159	2	1	162	8.81	0.11	0.08	8.99
Cdc	Silica gel	5.0	8	–	–	8	0.17	–	–	0.17
Cdc	mont.	8.4	11	1	–	11	0.55	0.04	–	0.59
Cdc	Hydroxy ap.	22.8	3	–	–	3	0.06	0.01	–	0.07
va	Diopsid	3.9	41	3	2	46	7.70	0.57	0.28	8.55
va	Olivine	4.7	1	–	–	1	0.15	–	–	0.15
va	Silica gel	2.8	8	–	–	8	1.47	0.01	–	1.48
va	mont.	3.0	–	1	1	2	–	0.08	0.08	0.15
va	Hydroxyl ap.	9.0	7	–	–	8	0.28	0.01	0.01	0.31

The masses of oxygenated products (oxy. p. in mg), n-alkanes (n-alk in mg), iso-alkanes (iso-alk in mg) and the total mass of all detected products (Σ, in mg) as well as the turnover number (TON) of oxygenated products (oxy. p. in g kg d), n-alkanes (n-alk in $\frac{{\text{g}}}{{{\text{kg}}\;{\text{d}}}}$), iso-alkanes (iso-alk in $\frac{{\text{g}}}{{{\text{kg}}\;{\text{d}}}}$) and the total TON of all detected products (Σ in $\frac{{\text{g}}}{{{\text{kg}}\;{\text{d}}}}$).

For the temperature and reaction time screening we were used various temperatures (150–300 °C) and reaction times (3–38 days) in CO_2_ fixation under conditions (p = 45 bar, H_2_:CO_2_ = 2:1, catalyst = synthetic *Campo del Cielo* at montmorillonite). The results are summarized in Table 6.

**Table 6:** Results of the screening of temperature (T in °C) and reaction time (t in d).

Conditions		m(products)				TON			
t	T	oxy. p	*n*-alk	*iso*-alk	Σ	oxy. p	*n*-alk	*iso*-alk	Σ
[d]	[°C]	[μg]				$$\frac{{\text{g}}}{{{\text{kg}}\;{\text{d}}}}$$			
3	300	682	152	120	953	2.84	0.63	0.50	3.97
38	300	438	60	127	625	0.15	0.02	0.04	0.21
7	250	437	3	–	440	0.77	–	–	0.77
7	200	258	3	–	261	0.45	0.01	–	0.46
17	170	2280	4	–	2283	1.79	–	–	1.79
14	150	934	10	4	948	0.86	0.01	–	0.88

The masses of oxygenated products (oxy. p. in mg), n-alkanes (n-alk in mg), iso-alkanes (iso-alk in mg) and the total mass of all detected products (Σ, in mg) as well as the turnover number (TON) of oxygenated products (oxy. p. in g kg·d), n-alkanes (n-alk in $\frac{{\text{g}}}{{{\text{kg}}\;{\text{d}}}}$), iso-alkanes (iso-alk in $\frac{{\text{g}}}{{{\text{kg}}\;{\text{d}}}}$) and the total TON of all detected products (Σ in $\frac{{\text{g}}}{{{\text{kg}}\;{\text{d}}}}$).

For the pressure and partial pressure screening we were used various pressures (9–45 bar) and ratios of H_2_:CO_2_ (1:9 to 9:1) in CO_2_ fixation under conditions (T = 300 °C, t = 3–4, catalyst = synthetic *Campo del Cielo* at montmorillonite). The results are summarized in Table 7.

**Table 7:** Results of the various pressures (p in bar), partial pressure of CO_2_ [p(CO_2_) in bar] and hydrogen pressure [p(H_2_) in bar].

Conditions			m(products)				TON			
p(H_2_)	p(CO_2_)	p	oxy. p	*n*-alk	*iso*-alk	Σ	oxy. p	*n*-alk	*iso*-alk	Σ
[bar]			[μg]				$$\frac{{\text{g}}}{{{\text{kg}}\;{\text{d}}}}$$			
6	3	9	33	12	16	61	0.14	0.05	0.07	0.27
14	7	21	9	9	9	27	0.04	0.04	0.04	0.12
23	12	35	209	23	19	252	1.29	0.14	0.12	1.55
30	15	45	682	152	120	953	1.75	0.05	0.06	1.86
5	40	45	517	20	38	575	3.34	0.13	0.25	3.72
24	21	45	1611	58	44	1713	6.87	0.25	0.19	7.30
23	23	46	656	21	9	686	4.35	0.14	0.06	4.54
28	17	45	1250	22	3	1275	5.15	0.09	0.01	5.26
21	24	45	727	15	13	755	4.73	0.10	0.08	4.91
19	26	45	746	57	30	834	4.99	0.38	0.20	5.57
15	30	45	406	13	14	433	1.75	0.05	0.06	1.86
27	19	45	846	35	25	906	5.37	0.22	0.16	5.75
41	5	45	34	12	4	51	0.11	0.04	0.01	0.16

The masses of oxygenated products (oxy. p. in mg), n-alkanes (n-alk in mg), iso-alkanes (iso-alk in mg) and the total mass of all detected products (Σ, in mg) as well as the turnover number (TON) of oxygenated products (oxy. p. in g kg d), n-alkanes (n-alk in $\frac{{\text{g}}}{{{\text{kg}}\;{\text{d}}}}$), iso-alkanes (iso-alk in $\frac{{\text{g}}}{{{\text{kg}}\;{\text{d}}}}$) and the total TON of all detected products (Σ in $\frac{{\text{g}}}{{{\text{kg}}\;{\text{d}}}}$).

The results of the experiments with water instead of hydrogen are summarized in Table 8. The conditions were T = 300 °C, t = 3–4, catalyst = *synthetic Campo del Cielo* at montmorillonite and pressure of CO_2_ = 40 bar.

**Table 8:** Results of the screening of water instead of hydrogen.

Conditions	m(products)			TON		
p(H_2_O)	oxy. p	*n*-alk	Σ	oxy. p	*n*-alk	Σ
[mL]	[μg]			$$\frac{{\text{g}}}{{{\text{kg}}\;{\text{d}}}}$$		
0.1	3	–	3	0.01	–	0.01
0.2	9	–	23	0.07	–	0.07
1.0	82	2	84	0.12	–	0.12

The masses of oxygenated products (oxy. p. in mg), n-alkanes (n-alk in mg), iso-alkanes (iso-alk in mg) and the total mass of all detected products (Σ, in mg) as well as the turnover number (TON) of oxygenated products (oxy. p. in g kg d), n-alkanes (n-alk in $\frac{{\text{g}}}{{{\text{kg}}\;{\text{d}}}}$), iso-alkanes (iso-alk in $\frac{{\text{g}}}{{{\text{kg}}\;{\text{d}}}}$) and the total TON of all detected products (Σ in $\frac{{\text{g}}}{{{\text{kg}}\;{\text{d}}}}$).

## Supplementary Information

Supplementary Information.

## Data Availability

All data are available in the main text or the supplementary materials.
