# Supplementary material for: Synthesis of prebiotic organics from CO2 by catalysis with meteoritic and volcanic particles
Source: Sci Rep. 2023 May 25;13:6843. doi: 10.1038/s41598-023-33741-8 (PMC10212956; doi:10.1038/s41598-023-33741-8)
Supplement: Supplementary file 1 — Supplementary Information. [file 41598_2023_33741_MOESM1_ESM.pdf]

Supporting Information: Synthesis of prebiotic organics from CO_2 by catalysis with meteoritic and volcanic particles

Sophia Peters, Dmitry A. Semenov, and Oliver Trapp*

Department of Chemistry, Ludwig Maximilians University, Butenandtstr. 5-13, D-81377 Munich, Germany and Max Planck Institute for Astronomy, Königstuhl 17, D-69117 Heidelberg, Germany

Rupert Hochleitner

Mineralogische Staatssammlung München (SNSB), Theresienstr. 41, D-80333 Munich, Germany

* oliver.trapp@cup.uni-muenchen.de

CONTENTS

I. Materials and Equipment S4

II. Analyses of the natural used materials S6

A. Analyses of the minerals S6

B. Analyses of the metals sources S7

III. Synthesis of the oxidized catalysts S8

A. Synthesis of the nanoparticular oxidized catalysts - wet impregnation S8

1. Stock solutions S8

2. Procedure for the synthesis of the supported oxidized catalysts S9

B. Preparation of the ball milled oxidized catalysts S10

IV. Analyses of the supported oxidized catalysts S12

V. Procedure for CO_2-fixations S15

A. Reduction of the supported oxidized catalysts S15

B. Reaction procedures S15

VI. Analyses of the products S16

A. Blank measurements S16

B. Separation and qualitative analysis of the resulting products S17

1. Quantitative gas chromatography analysis S23

2. Analyses of formaldehyde S25

VII. Results S26

A. Screening of the catalyst S26

B. Screening of the particle size S28

C. Temperature S30

D. Pressure S31

VIII. Mass estimate of prebiotic organics synthesized on the early Earth S32

A. Paleo-geological and environmental conditions S32

B. Delivery rate of exogenous meteoritic particles S33

C. Deposition rate of volcanic ash particles S36
D. Mass estimate of synthesized organics S37

IX. Reactions S38

References S92

I. MATERIALS AND EQUIPMENT

Anhydrous dichloromethane (Honeywell or Acros Organics, 99.8 %), iron nitrate nonahydrate (Alfa Aesar, 98.0 – 101.0 %), nickel nitrate hexahydrate (Alfa Aesar, 98 %), cobalt nitrate hexahydrate (Alfa Aesar, 98.0 – 101.0 %), germanium tetrachloride (Alfa Aesar, 99.99999 %), gallium nitrate (Sigma Aldrich, GA 9-10 % W/W), dimedone (Sigma Aldrich, ≥99.0 %), iridium chloride anhydrous (Strem Chemicals, 99.95+ %) were purchased and used as received. Hydrogen (99.999 %) and carbon dioxide (99.998 % or 99.995 %) were purchased from Air Liquid. Fused silica capillaries were purchased from MicroQuartz, Munich, Germany. The gas chromatography stationary phase GE-SE-30 was obtained from Macherey & Nagel, Düren, Germany. Montmorillonite was purchased from Alfa Aesar as 'naturally occurring mineral', 200 mesh powder and used as received. Silica gel was purchased from Acros Organics as 0.035-0.070 mm, 60 Å powder and used as received. *Campo del Cielo* was purchased from Decker Meteorite-Museum, Oberwesel, Germany, and used as received.

The meteorites *Muonionalusta*, and *Gao-Guenie*, the minerals olivine and diopside were provided by the 'Mineralogische Staatssammlung Bayern'. The minerals were powdered in the ball mill (400 rpm for 15 min).

The volcanic ash was collected from an eruption of the Etna, Sicily, Italy, on April 23rd 2012 in Fornazzo.

Hydroxy apatite was prepared according to the procedure of Hayek and Stadlmann[1]: Ammonium dihydrogen phosphate (79,0 g, 0,598 mmol, 1 eq) was dissolved in water (400 ml) and the pH was adjusted to 12 using ammonia. Calcium nitrate was dissolved in water and adjusted to a pH of 12 using ammonia. After addition of this solution a white precipitate was formed, which was filtered off, washed with water and then subsequently dried at 100 °C for 12 hours. The resulting white powder was calcined at 450 °C for 4 h and annealed at 800 °C for 2 h.

Forsterite [(Olivine syn)] was performed according to Burlitch et. al. [2]. For this purpose, magnesium chips (2.475 g, 101.8 mmol, 2 eq) and tetraethylsilane (10.595 g, 50.9 mmol, 1 eq) were solved in methanol (100 mL). A hydrogen peroxide solution (5.30 g of a 33 % solution, 46.7 mmol, 1.9 eq in 100 mL methanol) was added over a period of one hour. Subsequently, a water/methanol mixture (1.8 g, 0.10 mol, 1.8 eq in 100 mL methanol) was added over a 6 h

period. This solution was refluxed at 53°C for 8 h. From this point on, work was carried out under air. After cooling, the reaction batch was transferred to another vessel and toluene (300 mL) was added over a period of one hour. A tetrabutylammonium hydroxide solution (7.5 mg of a 40 % solution in 7.5 mL of methanol) was added over a period of 30 min. Methanol and ethanol were removed under reduced pressure. After centrifugation, the supernatant was washed alternately with toluene and cyclohexane (3 x 90 mL). The solvent was removed under reduced pressure. The resulting white powder was calcined 450°C for 12 h at and annealed at 1000°C for 12 h.

For the reductions and reactions under high pressure, a high-pressure stainless steel autoclave with a 200 mL glass insert with digital pressure gauges, fine throttling valve, and temperature sensor 330 mm. The autoclave is tightened with silver gasket. The temperature was adjusted by a heating hood 20 S, equipped with a magnetic stirrer. The autoclaves were purchased from Carl Roth, Karlsruhe, Germany.

For reactions under aqueous conditions an addition a three-way ball valve was installed between the autoclave and the fine throttling valve. The autoclaves were pressurized with a homebuild high pressure screening setup.[3]

The Planetary Ball Mill 33 Pulverisette 7 was purchased from Fritsch GmbH, Idar-Oberstein, Germany and was used with two grinding bowls of 20 mL stainless steel and 12 balls of 10 mm stainless steel.

A Thermo Trace gas chromatograph (San Jose, California, USA) equipped with a split-injector (250 °C), a flame ionization detector (250 °C) for the quantitative analyses, and for the identification a quadrupole ion trap (PolarisQ MS) mass spectrometer or quadrupole (ISQ single quadrupole MS) mass spectrometer was used, respectively. Gas chromatography analysis was performed on a 25 m GE-SE-30 250 nm (ID 250 μm).

II. ANALYSES OF THE NATURAL USED MATERIALS

A. Analyses of the minerals

As support minerals four silicates (diopside, olivine, montmorillonite and silica gel) and a phosphate mineral (hydroxy apatite) were chosen, that have been already present on the Earth. The morphology and composition were analyzed by Scanning Electron Microscope (SEM) and Energy Dispersive X-ray Spectroscopy EDX. The elemental composition is summarized in Table S1.

Table S1. Summarized properties and composition of the mineral supports: Name, molecular formula, category, and mass percent of oxygen (O, $\%_{atom}$), magnesium (Mg, $\%_{atom}$), silicon (Si, $\%_{atom}$), calcium (Ca, $\%_{atom}$), iron (Fe, $\%_{atom}$), sodium (Na, $\%_{atom}$), aluminum (Al, $\%_{atom}$), and phosphorus (P, $\%_{atom}$) determined by scanning electron microscopy (SEM).

Name	Molecular formula	Category	O	Mg	Si	Ca	Fe	P	Na	Al
			[$\%_{atom}$]							
Olivine	$(Mg,Fe)_2[SiO_4]$	Nesosilicate	57.24	26.10	14.24	-	2.42	-	-	-
Olivine (syn.)	$Mg_2[SiO_4]$	Nesosilicate	56.44	29.11	14.45	-	-	-	-	-
Diopside	$MgCaSi_2O_6$	Inosilicate	60.21	9.81	20.08	9.89	-	-	-	-
Montmorillonite	$(Na,Ca)_{0.33}(Al,Mg)_2$ $(Si_4O_{10})(OH)_2 \cdot nH_2O$	Phyllosilicate	63.64	1.41	22.84	0.78	1.24	-	1.31	8.78
Silica gel	SiO_2	-	66.51	-	33.49	-	-	-	-	-
Hydroxy apatite	$Ca_5[OH\|(PO_4)_3]$	Phosphate	64.33	-	-	22.06	-	13.61	-	-

B. Analyses of the metals sources

Various metal precursors, which have been present on the Earth, were selected catalytically active materials: iron and stone meteorites and volcanic ash. These materials are composed of consisting of iron, nickel, and other trace metals.

The composition of these materials is summarized in Table S2 and Table S3.

Table S2. Summarized composition of the metal precursors: Name, mass percent of iron (Fe, $\%_{mass}$), nickel (Ni, $\%_{mass}$), cobalt (Co, $\%_{mass}$), phosphorus (P, $\%_{mass}$), gallium (Ga, ppm), germanium (Ge, ppm), iridium (Ir, ppm), silicates ($\%_{mass}$), and carbon ($\%_{mass}$).

Name	Silicates	Carbon	Fe	Ni	Co	P	Ga	Ge	Ir
	[$\%_{mass}$]						[ppm]		
Campo del Cielo	-	-	92.6	6.68	0.43	0.25	87	407	3.6
Muonionalusta	-	-	91.3	8.7	-	-	0.33	0.13	1.6
Gao-Guenie	79.43	0.20	20.37		-	-	-	-	-

Table S3. Summarized composition of the volcanic ash measured by ICP: mass fraction in milligram per gram volcanic ash of iron (Fe, mg/g), nickel (Ni, mg/g), cobalt (Co, mg/g), phosphorus (P, mg/g), gallium (Ga, mg/g), germanium (Ge, mg/g), iridium (Ir, mg/g), silicon (Si, mg/g), calcium (Ca, mg/g), chrome (Cr, mg/g). copper (Cu (mg/g), potassium (K, mg/g), magnesium (Mg, mg/g), manganese (Mn, g/mg), sodium (Na, mg/g), sulfur (S, mg/g), titanium (Ti, mg/g), and zinc (Zn, mg/g).

Elements	Fe	Ni	Co	P	Ga	Ge	Ir	Si	Ca
Mass fraction [mg/g]	119.60	0.01	0.04	9.22	0.05	0.01	0.03	175.65	46.55
Elements	Cr	Cu	K	Mg	Mn	Na	S	Ti	Zn
Mass fraction [mg/g]	0.01	0.01	16.93	18.45	2.22	15.97	1.63	17.80	0.25

III. SYNTHESIS OF THE OXIDIZED CATALYSTS

A. Synthesis of the nanoparticular oxidized catalysts - wet impregnation

Nanoparticular oxidized catalysts were prepared by wet impregnation, described in reference 4, using aqueous solutions of meteorites or volcanic ash (stock solutions), dissolved in aqueous nitric acid, and the corresponding powdered support materials.

1. *Stock solutions*

Preparation of the stock solution for the synthetic Campo del Cielo

For the initial screening of the reaction conditions in the autoclave setup, we prepared a stock solution according to the composition of *Campo del Cielo*. In all other experiments catalytically active materials were prepared from the authentic meteoritic and volcanic materials.

Iron nitrate nonahydrate (13.3976 g), nickel nitrate hexahydrate (0.6598 g), cobalt nitrate hexahydrate (24.8 mg), and iridium chloride (1.065 M in 0.356 mM in potassium hydroxide solution, 353 μl) were dissolved in 1 L deionized water. This composition (92.6 % iron, 6.68 % nickel, 0.25 % cobalt, and 3.6 ppm iridium) corresponds to the composition of the meteorite *Campo del Cielo*.

Preparation of the stock solution for the authentic meteoric and volcanic ash

The meteorites and the volcanic ash were dissolved in aqueous nitric acid yielding the stock solutions. The stone meteorite and the volcanic ash are not completely dissolved under these conditions, therefore the solutions were disperged and used without filtration.

Table S4. Summarized parameters for the stock solutions of the metal precursors: Metal precursor, mass of the metal precursor (m, in g), volume of nitric acid (V_1, in mL), volume of water (V_2, in mL), time of the dissolving process (t, in d), and resulting concentration of the stock solution (c, in mg/mL).

Metal precursors	m [g]	V_1 [mL]	V_2 [mL]	time [d]	c [mg/mL]
Campo del Cielo	0.3016	36	37	1	41.31
Muonionalusta	0.4966	20	11	1	16.02
Volcanic ash	0.4341	20	5	21	17.36
Gao-Guenie	0.4511	20	5	21	18.04

2. Procedure for the synthesis of the supported oxidized catalysts

For the preparation of the supported oxidized catalysts, the support (silica gel, hydroxy apatite, olivine, diopside and montmorillonite clay) was impregnated with the stock solution. The prepared suspension was dried at 100 °C and subsequently calcined at 450 °C for 4.5 h. Under these conditions the metal nitrates completely decompose under formation of the corresponding metal oxides.

Table S5. Summarized parameters for the syntheses of the nanoparticle pre-catalysts: Metal precursors, support, concentration of the stock solution (c, in mg/mL), volume of the stock solution (V, in mL), mass of the support (m, in g), and percentage share of the support (composition, in %).

metal Precursors	Support	c [mg/mL]	V [mL]	m [g]	Composition [%]
Muonionalusta	Diopside	16.02	5	2.4691	96.8
Muonionalusta	Olivine	16.02	5	2.5001	96.8
Muonionalusta	Hydroxy apatite	16.02	5	1.6510	96.8
Muonionalusta	Montmorillonite	16.02	37	6.8952	92.2
Muonionalusta	Silica gel	16.02	37	7.0213	92.2
Campo del Cielo	Diopside	4.13	40	2.4557	93.7
Campo del Cielo	Olivine	4.13	40	2.4635	93.7
Campo del Cielo	Hydroxy apatite	4.13	40	2.3056	93.3
Campo del Cielo	Montmorillonite	4.13	30	1.556	92.6
Campo del Cielo	Silica gel	4.13	30	1.6510	93.0
Volcanic ash	Diopside	17.36	5	1.0322	92.2
Volcanic ash	Olivine	17.36	5	1.0308	92.2
Volcanic ash	Hydroxy apatite	17.36	5	1.4604	94.4
Volcanic ash	Silica gel	17.36	5	0.8395	90.6
Volcanic ash	Montmorillonite	17.36	5	1.3633	94.2
Gao-Guenie	Diopside	18.04	5	1.2611	93.3
Gao-Guenie	Olivine	18.04	5	0.9338	91.2
Gao-Guenie	Hydroxy apatite	18.04	5	1.2540	93.3
Gao-Guenie	Silica gel	18.04	5	1.3208	93.6
Gao-Guenie	Montmorillonite	18.04	5	1.2052	93.1

B. Preparation of the ball milled oxidized catalysts

For preparation of the ball milled oxidized catalysts from the *Campo del Cielo* meteorite, the meteorite was dissolved in aqueous nitric acid (*vide supra*)(S4). This solution was dried

at 100 °C. The resulting powder or volcanic ash was mixed with the support materials (silica gel, hydroxy apatite, olivine, diopside and montmorillonite clay) and milled in the ball mill at 800 rpm for 15 min.

Table S6. Summarized parameters for the preparation the ball milled oxidized catalysts: Metal precursor, support, mass of metal precursors (m_1, in mg), mass of support (m_2, in g), rotation speed of the ball mill (rotation, in rpm), time of the rotation of the ball mill (t, in min), and weight of the support (composition, in %).

Metal precursors	Support	m_1[mg]	m_2 [g]	rotation [rpm]	t [min]	Composition [%]
Volcanic ash	montmorillonite	45.2	1.5041	400	15	97.1
Volcanic ash	silica gel	33.8	1.5211	400	15	97.8
Volcanic ash	olivine	63.2	1.6049	400	15	96.2
Volcanic ash	diopside	41.6	2.4054	400	15	97.1
Volcanic ash	hydroxy apatite	48.0	1.5014	400	15	92.7
Campo del Cielo	montmorillonite	13.9	1.5083	400	15	99.1
Campo del Cielo	silica gel	13.3	2.5108	400	15	99.1
Campo del Cielo	olivine	6.4	1.5021	400	15	99.6
Campo del Cielo	diopside	4.9	1.4839	400	15	99.7
Campo del Cielo	hydroxy apatite	35.3	1.5170	400	15	97.7

IV. ANALYSES OF THE SUPPORTED OXIDIZED CATALYSTS

Particle size and metal ratio of the supported nanoparticlar oxidized catalysts were analyzed by scanning electron microscopy (SEM) (FEI Helios G3 with EDX-detector) and energy-dispersive X-ray spectroscopy (EDX). For better conductibility, the samples were sputtered with carbon. The nanoparticle size distribution was determined by ImageJ. The statistical evaluation was performed in Python 3.6. The oxidized catalysts were characterized by scanning electron microscopy (SEM) imaging. Exemplary pictures are shown below.

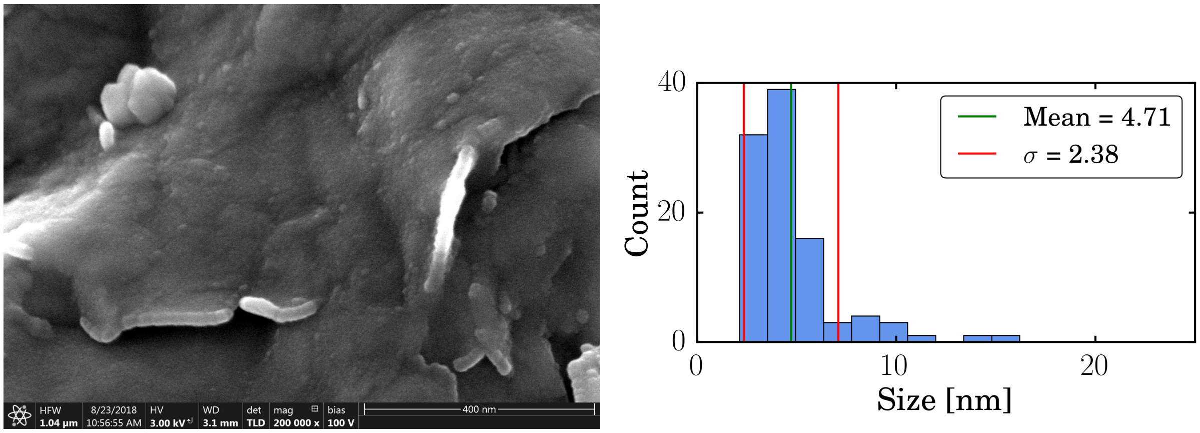

Figure S1. Scanning electron microscopy (SEM) picture of oxidized *Campo del Cielo* on the support montmorillonit clay (left) and size distribution of the nanoparticles (right).

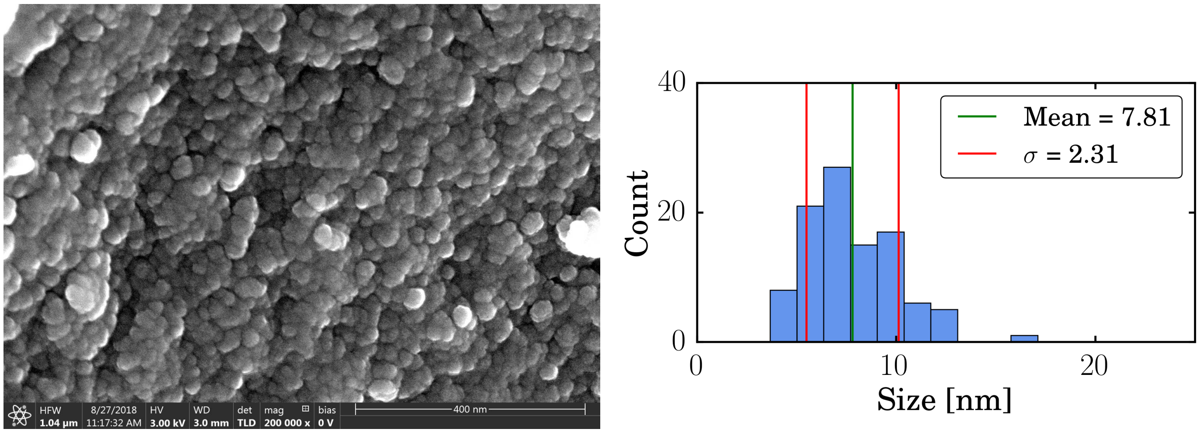

Figure S2. Scanning electron microscopy (SEM) picture of oxidized *Campo del Cielo* on the support silica gel (left) and size distribution of the nanoparticles (right).

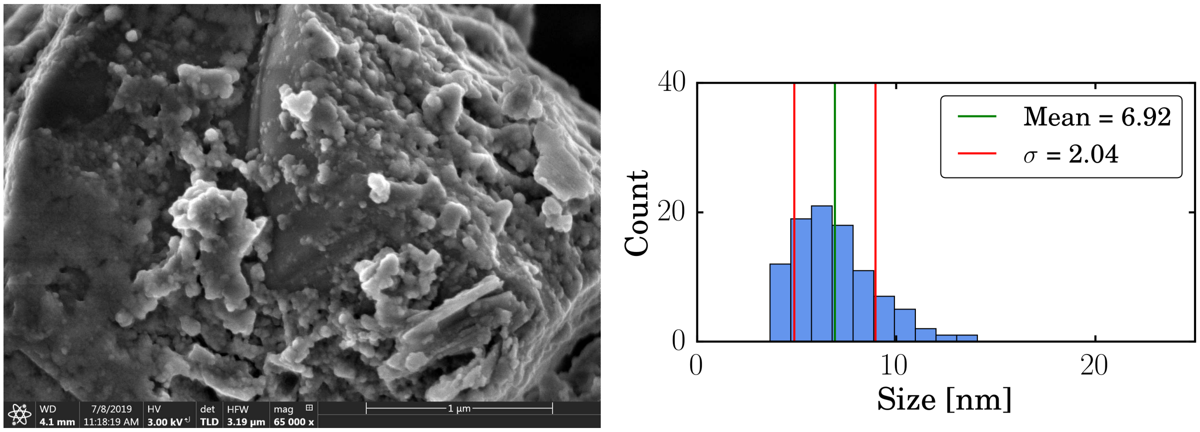

Figure S3. scanning electron microscopy (SEM) picture of oxidized *Campo del Cielo* on the support olivine (left) and size distribution of the nanoparticles (right).

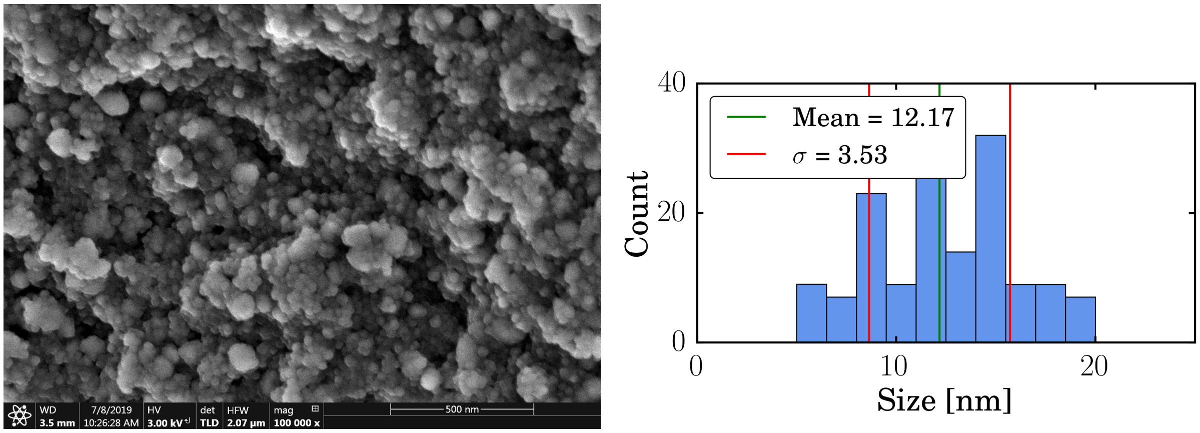

Figure S4. scanning electron microscopy (SEM) picture of oxidized *Campo del Cielo* on the support diopside (left) size distribution of the nanoparticles (right).

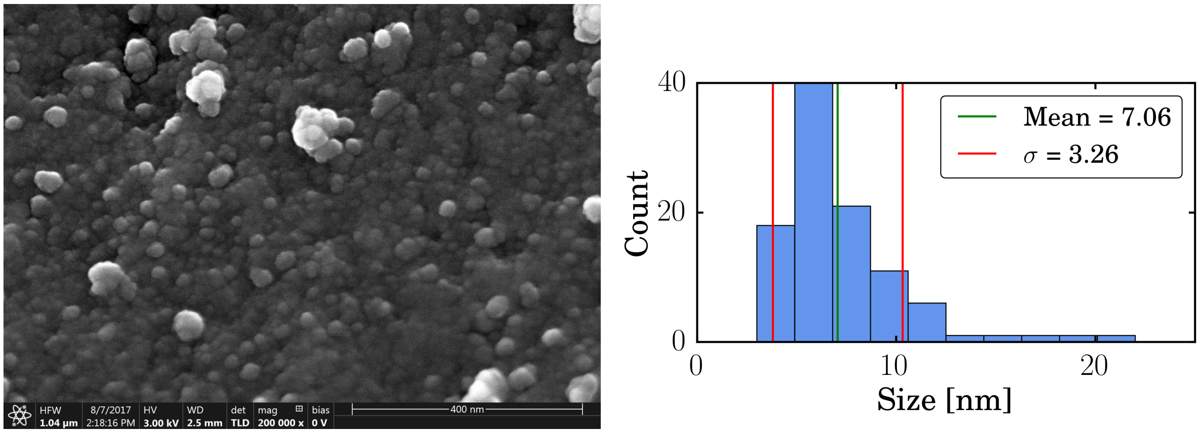

Figure S5. scanning electron microscopy (SEM) picture of oxidized *Campo del Cielo* on the support hydroxyl apatite (left) and size distribution of the nanoparticles (right).

The analysis of the ball mill catalysts is not possible *via* this analysis because they are magnetic (no complete oxidation). The manufacturer Fritsch GmbH, Idar-Oberstein, Germany stated that the particles do not become smaller than 10 μm with this kind of ball mill.

V. PROCEDURE FOR CO_2-FIXATIONS

A. Reduction of the supported oxidized catalysts

For the reduction of the oxidized catalysts ($\approx$1 g) of the impregnated support materials was transferred in the glass insert (quartz glass) of the autoclave. The autoclave was evacuated and flushed with nitrogen (three times). After evacuation (9×10^{-3} mbar), hydrogen ($\approx$ 50 bar) was pressurized into the autoclave. Reduction of the oxidized catalysts was achieved by heating to $\approx$ 300 °C for 17 h.

B. Reaction procedures

Prior reaction the excess hydrogen of the reduced and cooled down supported catalyst was released and carbon dioxide and, subsequently, hydrogen or water were added with a defined partial pressure or volume, respectively. The pressurized autoclave was then heated to the corresponding temperature. The detailed reaction conditions are listed in Section VII or IX. After the set reaction time, the autoclave was cooled to 0 °C (to condense the volatile compounds). In order to separate the formed organic compounds from the catalyst, a distillation was performed. The black, frozen catalyst was cooled to -180 °C and transferred to the glass distillation apparatus. This apparatus was evacuated 3×10^{-2} mbar. Then, the leg of the distillation apparatus with the catalyst was heated to 210 - 220 °C and the evaporated compounds were condensed into a flask cooled with liquid nitrogen (-180 °C). After completion of the distillation process the apparatus was opened and after a warming the reaction products were collected by adding dichloromethane (3× overall 0.3 mL or 0.5 mL).

VI. ANALYSES OF THE PRODUCTS

A. Blank measurements

The following blank measurements were performed to exclude contaminations of the dichlormethane, catalyst, distillation, minerals, metal sources, ball mill and autoclave system: 1. dichloromethane (the solvent used), 2. the catalyst reduced in the autoclave and then distilled (without CO_2 addition), 3. the reaction in the autoclave system without catalyst, and 4. the minerals (diopsid, hydroxy apatit, and olivine milled in the ball mill) used for the catalyst were reduced without pretreatment, as in a CO_2 fixation, and then used under standard conditions (T = 300 °C, p = 45 bar, H_2:CO_2 = 2:1, t = 3-4) in the CO_2 fixation. The detected products are summarized in the following table (table S7)

Table S7. Blank measurement with catalysts: metal source and mineral, the conditions: reaction times (t, in d), ratio of partial pressures of hydrogen and carbon dioxide (H_2:CO_2) and the overall pressure (p, in bar) and with the resulting products: Mass of oxygenated products (oxy. p., in mg), *n*-alkanes (*n*-alk, in mg), *iso*-alkanes (*iso*-alk, in mg) and the total mass of all detective products (m_{Total}, in mg) in mg. The temperature was in all experiments 300 °C

catalysts		conditions			m(products				for more
metal source	minerals	t [d]	H_2:CO_2	p [bar]	oxy. p. [μg]	*n*-alk	*iso*-alk	m_{Total} [μg]	information see
blank	blank	Dichlormethan			1	-	-	1	p. S80
blank	blank	5	2:1	45	-	-	-	-	p. S81
Campo del Cielo	silica gel	1	1:0	50	1	-	-	1	p. S40
blank	Silica gel	6	2:1	45	-	1	-	1	p. S58
blank	Montmorillonite	6	2:1	45	-	4	-	4	p. S57
blank	Diopsid	3	2:1	45	-	-	-	-	p. S62
blank	Hydroxy apatite	2	2:1	45	-	2	-	2	p. S77
blank	Olivine	4	2:1	45	2	-	-	2	p. S62
Muonionalusta	blank	3	2:1	45	2	-	-	2	p. S58
Campo del Cielo	blank	3	2:1	45	15	1	-	16	p. S82
Guenie-Gao	blank	2	2:1	45	23	1	-	24	p. S64
volcanic ash	blank	2	2:1	45	-	-	-	-	p. S60

B. Separation and qualitative analysis of the resulting products

Separation of the complex reaction mixtures has been achieved by gas chromatography employing a 25 m GE-SE-30 250 nm (ID 250 μm) separation column. The following temperature program was applied: 4 min at 40 °C, heating to 180 °C 4 °C/min, and 10 min keeping at 180 °C. The separation was performed under isobar conditions at 80 kPa and He was used as carrier gas. The identification of the reaction products was achieved by coupling to EI (70 eV) mass spectrometer. The EI mass spectra were measured by quadrupole ion trap (PolarisQ MS) or quadrupole (ISQ single quadrupole MS) MS. The compounds were identified

by comparison of the fragmentation patterns of mass spectra in the NIST Database, based on retention times and measurements of reference compounds (*n*-hexane, *n*-heptane, *n*-octane, *n*-nonane, *n*-decane, *n*-undecane, *n*-dodecane, *n*-tridecane, *n*-tetradecane, *n*-pentadecane, *iso*-alkanes (2-methyl pentane, 3-methyl pentane, 2-methyl hexane, 3-methyl heptane, 3-methyl octane, methanol and ethanol). These reference measurements were also used for quantification. The gas chromatography-MS spectra were evaluated using XCalibur software, Thermo, San Jose, California. Exemplary chromatograms are shown in the following figures.

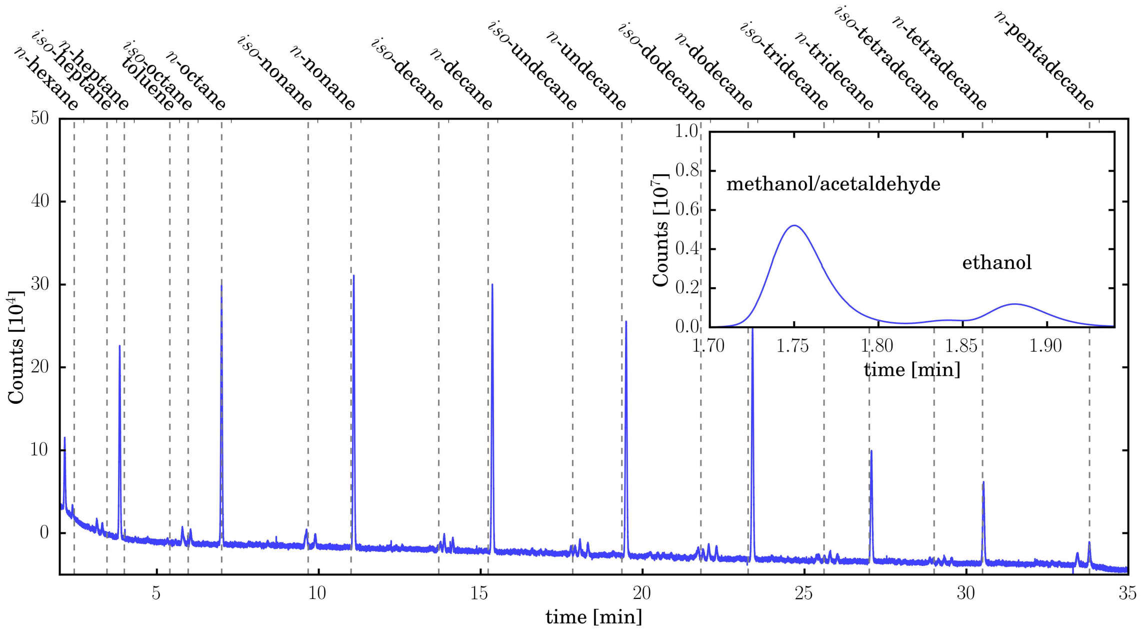

Figure S6. Liquid phase analysis: Gas chromatogram of the reaction of 2:1 H_2, CO_2 and *Campo del Cielo* on montmorillite as catalyst at T = 300 °C, p = 45 bar, p(CO_2) = 15 bar, p(H_2) = 30 bar, t = 3 d.

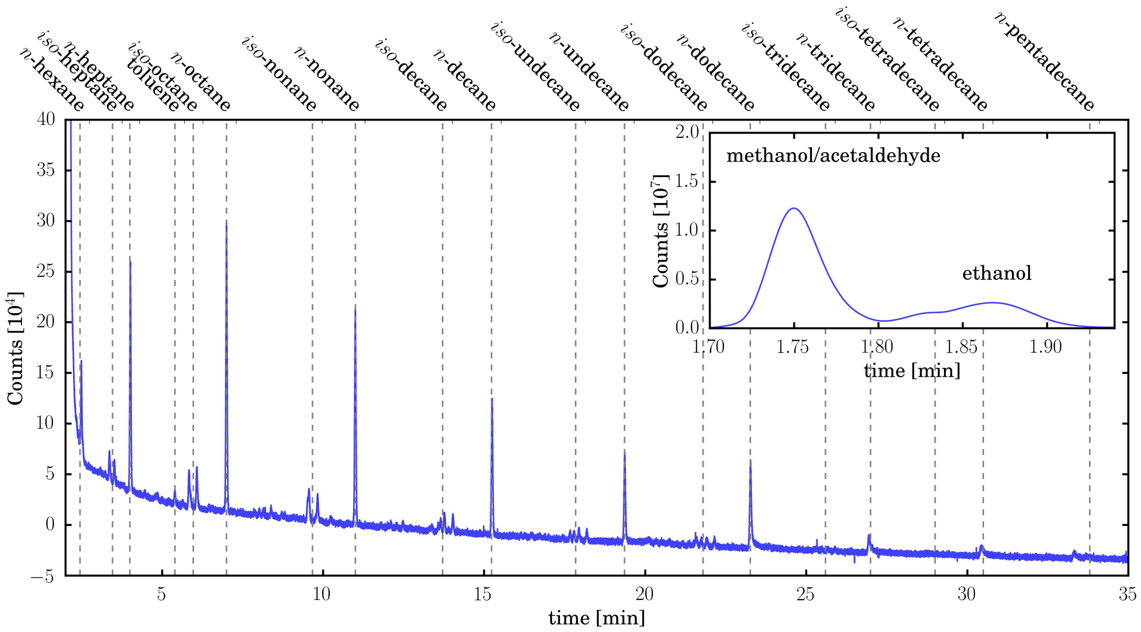

Figure S7. Liquid phase analysis: Gas chromatogram of the reaction of 2:1 H_2, CO_2 and *Campo del Cielo* on montmorillite as catalyst at T = 300 °C, p = 45 bar, p(CO_2) = 21 bar, p(H_2) = 24 bar, t = 3 d.

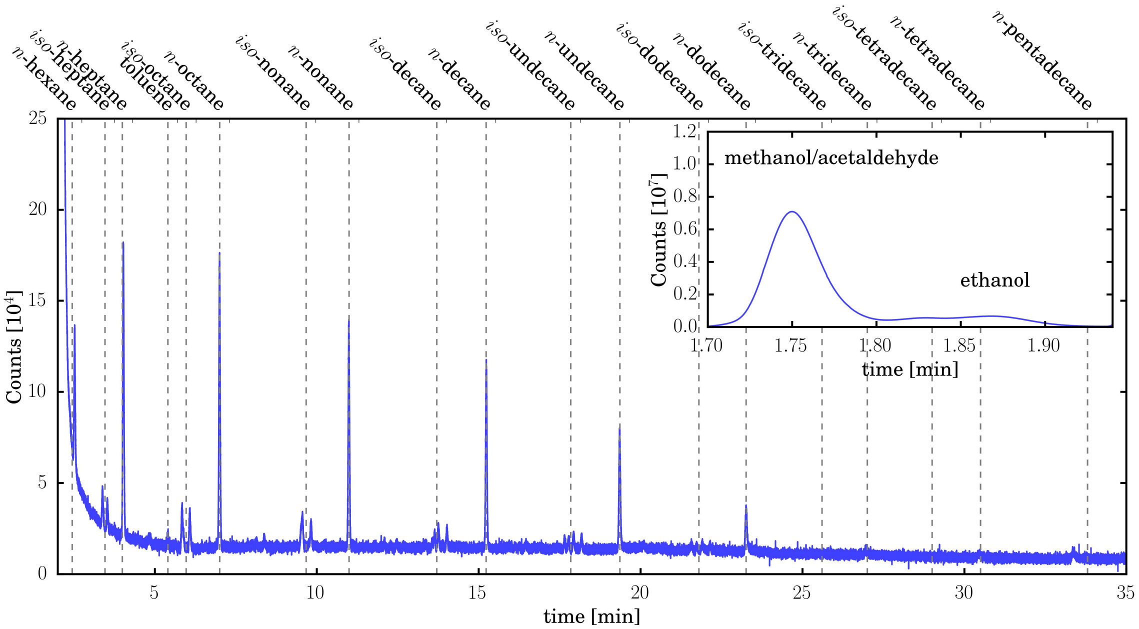

Figure S8. Liquid phase analysis: Gas chromatogram of the reaction of 2:1 H_2, CO_2 and *Campo del Cielo* on montmorillite as catalyst at T = 300 °C, p = 45 bar, p(CO_2) = 19 bar, p(H_2) = 27 bar, t = 2 d.

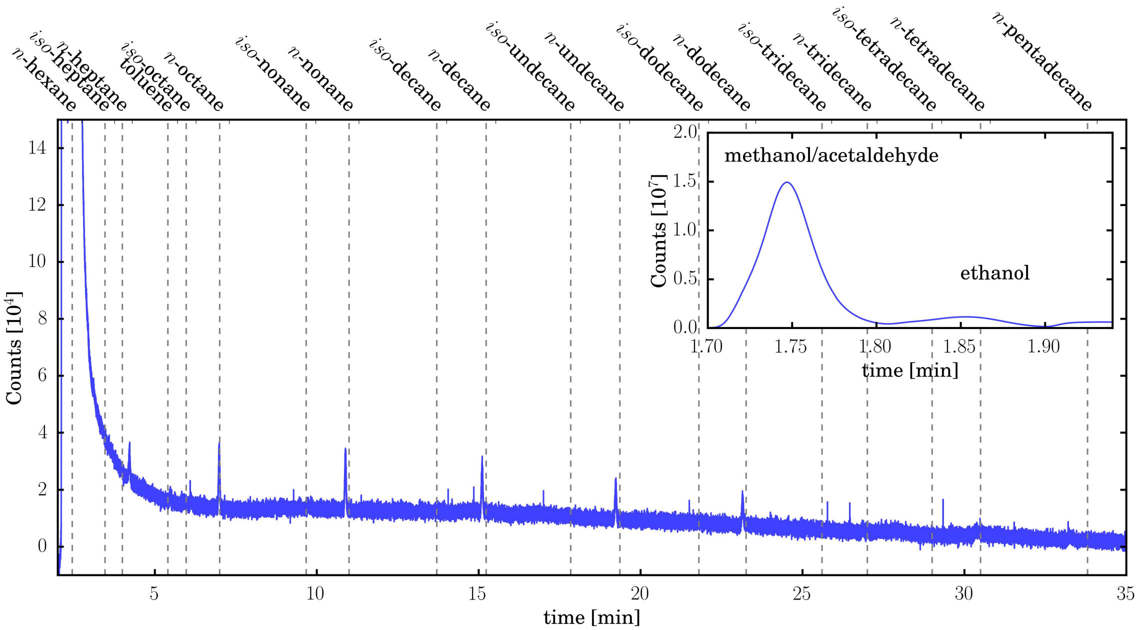

Figure S9. Liquid phase analysis: Gas chromatogram of the reaction of 2:1 H_2, CO_2 and *Campo del Cielo* on montmorillite as catalyst at T = 250 °C, p = 45 bar, p(CO_2) = 15 bar, p(H_2) = 30 bar, t = 7 d.

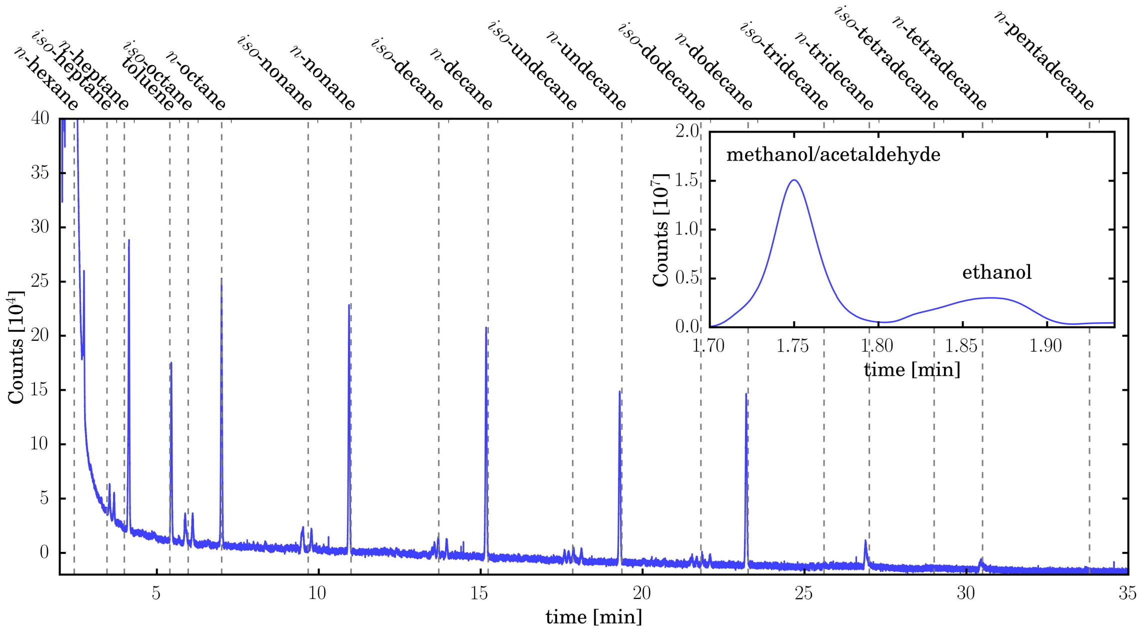

Figure S10. Liquid phase analysis: Gas chromatogram of the reaction of 2:1 H_2, CO_2 and *Campo del Cielo* on montmorillite as catalyst at T = 300 °C, p = 45 bar, p(CO_2) = 22.5 bar, p(H_2) = 22.5 bar, t = 2 d.

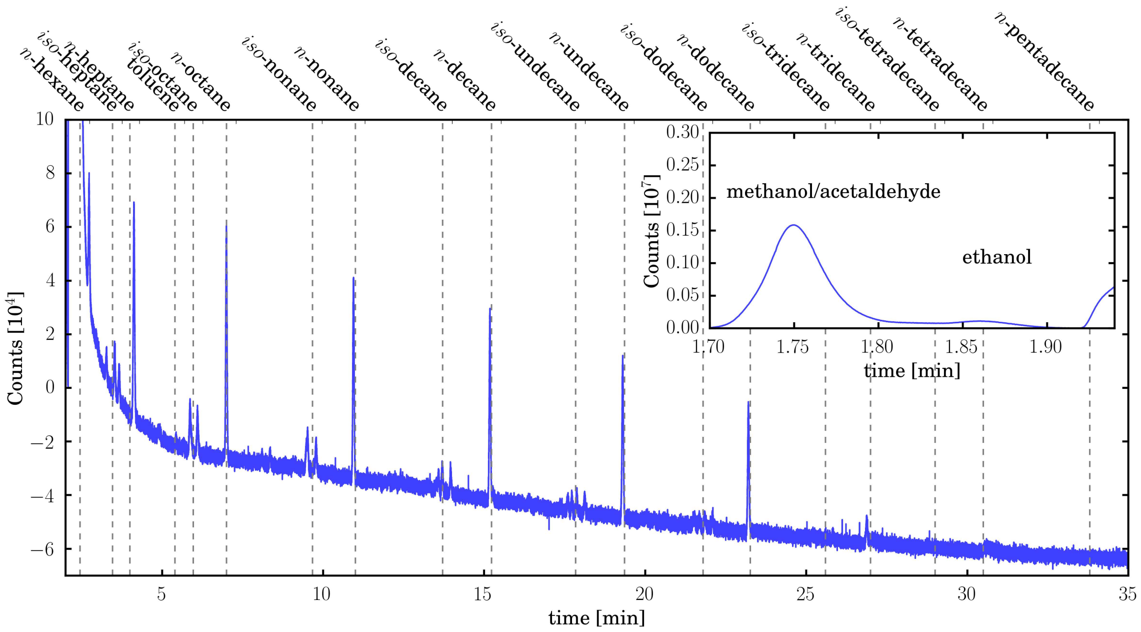

Figure S11. Liquid phase analysis: Gas chromatogram of the reaction of 2:1 H_2, CO_2 and *Campo del Cielo* on montmorillite as catalyst at T = 300 °C, p = 45 bar, p(CO_2) = 12 bar, p(H_2) = 23 bar, t = 2 d.

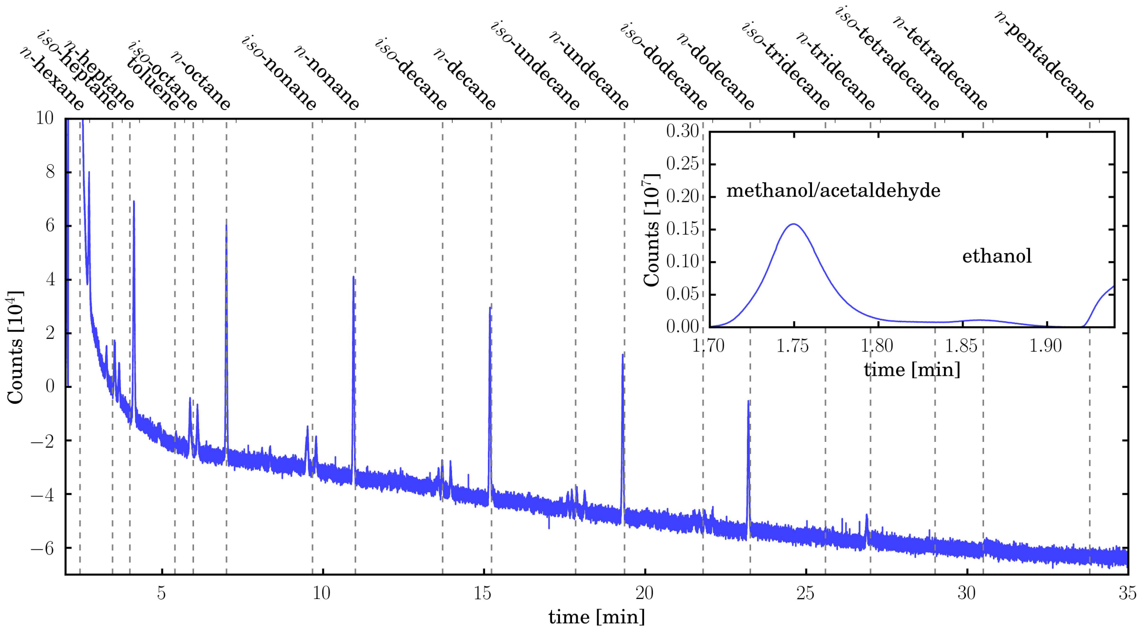

Figure S12. Liquid phase analysis: Gas chromatogram of the reaction of 2:1 H_2, CO_2 and *Campo del Cielo* on montmorillite as catalyst at T = 300 °C, p = 45 bar, p(CO_2) = 26 bar, p(H_2) = 19 bar, t = 2 d.

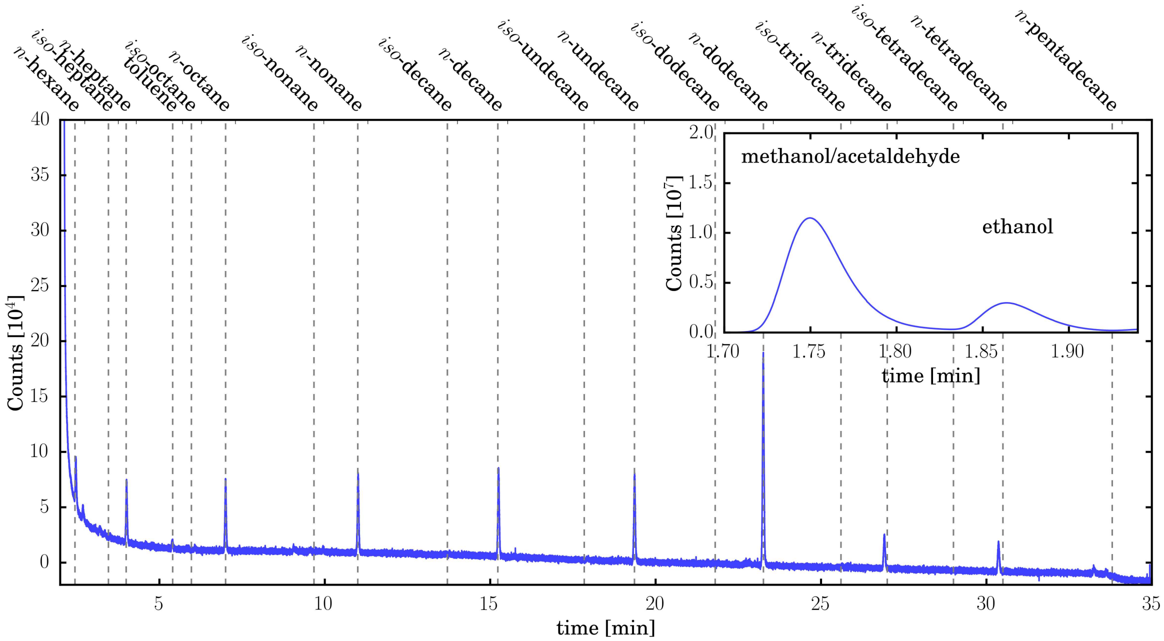

Figure S13. Liquid phase analysis: Gas chromatogram of the reaction of 2:1 H_2, CO_2 and *Campo del Cielo* on silica gel as catalyst at T = 300 °C, p = 45 bar, p(CO_2) = 15 bar, p(H_2) = 30 bar, t = 2 d.

1. Quantitative gas chromatography analysis

Quantification was achieved by calibration with dilution series of n-alkanes (n-hexane, n-heptane, n-octane, n-nonane, n-decane, n-undecane, n-dodecane, n-tridecane, n-tetradecane, and n-pentadecane), alcohols (methanol and ethanol) and *iso*-alkanes (2-methyl pentane, 3-methyl pentane, 2-methyl hexane, 3-methyl heptane, 3-methyl octane) using FID detection. Methanol and acetaldyde can not be separated under these conditions. Therefore, the methanol/acetaldehyde ratio was determined using the intensity of two fragments ($m/z = 31$ and $m/z = 44$) in mass spectra. By comparing to a reference measurement, the ratio was found to be $\approx 1\%$. The low share of acetaldehyde is probably a result of its very high volatility (boiling point is 20 °C), which leads to loses during the work-up. Evaluation was achieved with the QuanBrowser of Xcalibur, which performs regression analyses of the standards. Examples of calibration plots (methanol, n-octane, 2-methyl octane and 3-methyl octane) are shown in the following figure S14.

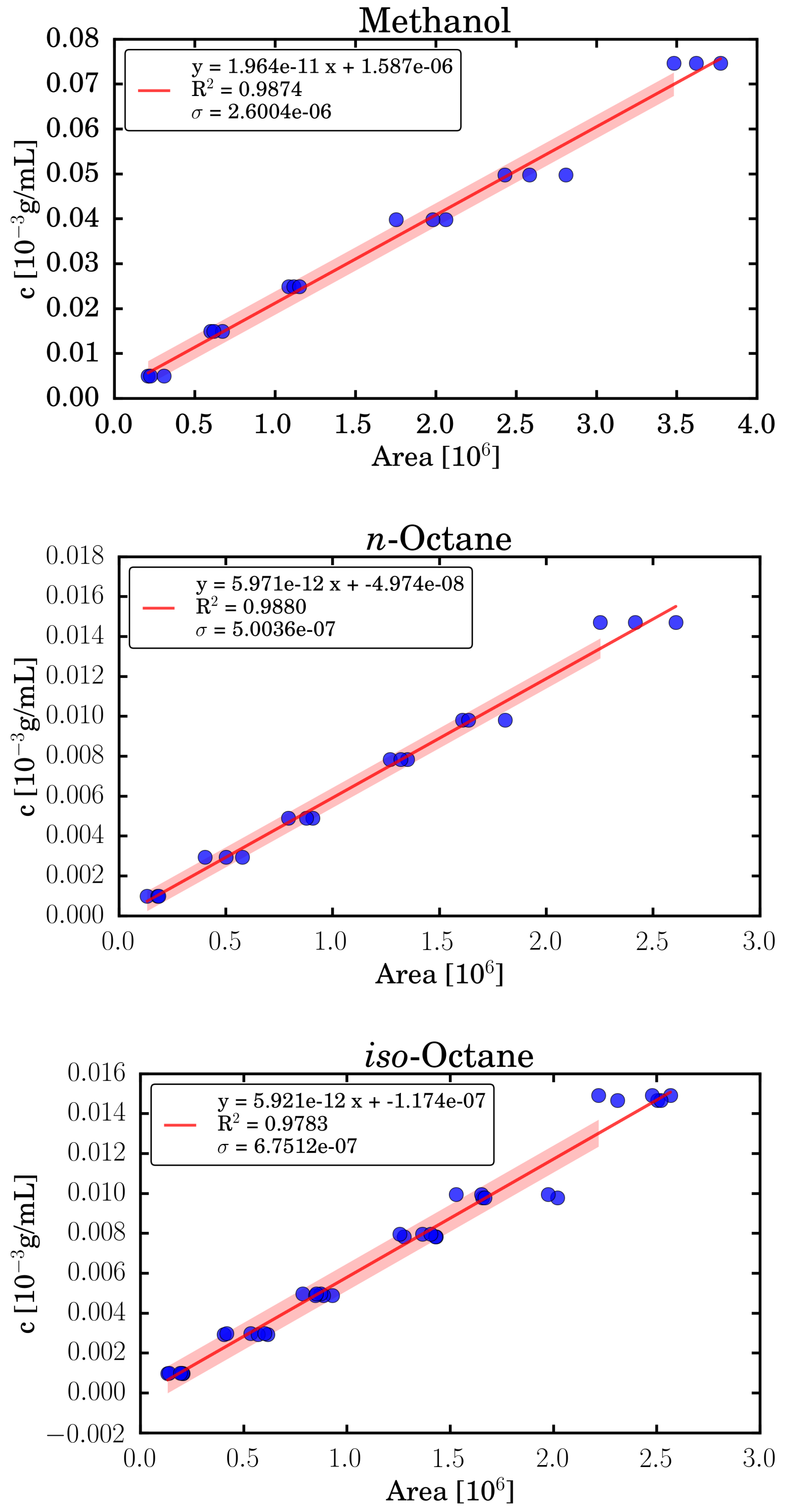

Figure S14. Dilution series of methanol, *n*-octane, and 2-methyl octane together with 3-methyl octane are plotted against the peak areas in the FID chromatogram. The linear regression analysis provide the correlation factors for quantification. R^2 represents the correlation coefficient, σ the standard deviation of the linear regression analyses.

2. Analyses of formaldehyde

Because formaldehyde is highly volatile compound, which is difficult to capture and quantify, we performed the following procedure. The volatiles in the gas phase of the autoclave after reaction were separated from carbon dioxide and hydrogen by passing through by a cooling trap (-78 °C). The outlet is fed into an aqueous dimedone methanol solution at -10°C. After completion this solution has been stirred at room temperature. Gas chromatography-MS analysis was performed to detect 3-hydroxy-2-((2-hydroxy-4,4-dimethylcyclohexyl)methyl)-5,5-dimethylcyclohexan-1-one. For the gas chromatography analysis the injector was set to 300 °C and following temperature program was applied: 4 min at 60 °C, heating to 270 °C 10 °C/min, and 30 min keeping at 270 °C. The separation was performed under isobar conditions at 80 kPa and He was used as carrier gas.

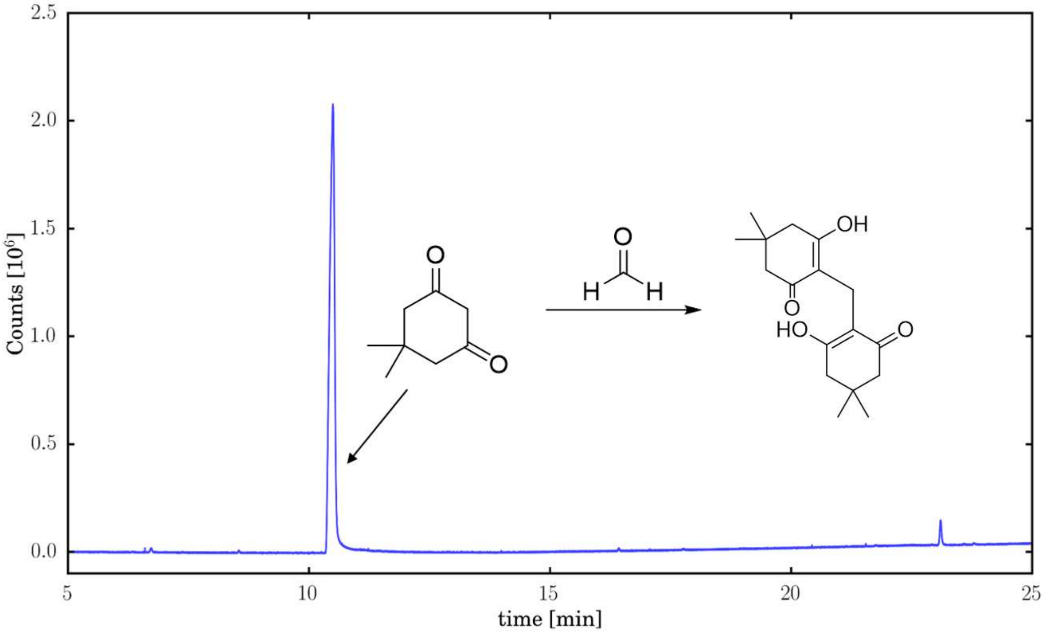

Figure S15. Formaldehyde detection: Gas chromatogram of the methanolic dimedone solution after derivatisation ion reaction of the gas phase of the reaction under following conditions: T = 250 °C, p = 21 bar, p(CO_2) = 20 bar, p(H_2) = 1 bar, t = 10 d and *Campo del Cielo* on montmorillite as catalyst. The resulting peaks can be assigned to reactant and product of the reaction, confirming the presence of formaldehyde.

VII. RESULTS

A. Screening of the catalyst

For the catalyst activities we were used the catalysts in CO_2 fixation under standard conditions (T = 300 °C, p = 45 bar, H_2:CO_2 = 2:1, t = 3-4). The results are summarized in table S8 and S9.

Table S8. Results of the screening of nanoparticular catalysts with the metal source: *Campo del Cielo* (Cdc), volcanic ash (va), *Muonionalusta* (Mn), Guenie-Gao (Gg), the supports Diopsid, silica gel, montmorillonite (mont.), and the metal concentration. The masses of oxygenated products (oxy. p., in mg), *n*-alkanes (*n*-alk, in mg), *iso*-alkanes (*iso*-alk, in mg) and the total mass of all detected products (Σ, in mg) as well as the turnover number (TON) of of oxygenated products (oxy. p., in $\frac{g}{kg \times day}$), *n*-alkanes (*n*-alk, in $\frac{g}{kg \times day}$), *iso*-alkanes (*iso*-alk, in $\frac{g}{kg \times day}$) and the total TON of all detected products (Σ, in $\frac{g}{kg \times day}$).

catalyst			m(products)				TON				for more
metal source	mineral	m(Met.) [‰]	oxy. P. [μg]	*n*-alk [μg]	*iso*-alk [μg]	Σ [μg]	oxy. P. [$\frac{g}{kg \times day}$]	*n*-alk [$\frac{g}{kg \times day}$]	*iso*-alk [$\frac{g}{kg \times day}$]	Σ [$\frac{g}{kg \times day}$]	information see
va	diopsid	8.9	1588	23	2	1613	84.20	1.22	0.08	85.50	pS75
Mn	diopsid	38.6	909	1	-	910	17.12	0.02	-	17.14	p.S63
Cdc	diopsid	73.3	2130	74	4	2208	7.13	0.25	0.01	7.39	p. S83
Gg	diopsid	10.6	-	3	-	3	-	0.08	-	0.08	p. S71
va	silica gel	8.8	1066	6	6	1077	37.99	0.22	0.20	38.41	p. S73
Cdc	silica gel	7.4	5365	118	55	5539	35.81	0.79	0.37	36.97	p. S56
Mn	silica gel	85.8	2852	77	-	2930	16.61	0.45	-	17.06	p. S79
Gg	silica gel	10.5	-	-	-	-	-	0.01	-	0.01	p. S72
Cdc	mont.	73.9	682	152	120	953	2.84	0.63	0.50	3.97	p. S80
Mn	mont.	73.8	266	10	4	280	1.78	0.07	0.02	1.88	p. S55
va	mont.	6.1	13	1	-	14	0.81	0.04	0.02	0.87	p. S74
Gg	mont.	13.3	17	-	-	18	0.60	0.00	-	0.60	p. S72

Table S9. Results of the screening of nanoparticular catalyst with the metal source: *Campo del Cielo* (Cdc), volcanic ash (va), *Muonionalusta* (Mn), Guenie-Gao (Gg), the supports Olivine, hydroxy apatite (hydroxy ap.), calcium carbonate (calcium carb.), synthesized olivine [olivine (syn)] and the metal concentration. The masses of oxygenated products (oxy. p., in mg), n-alkanes (n-alk in mg), *iso*-alkanes (*iso*-alk, in mg) and the total mass of all detected products (Σ, in mg) as well as the turnover number (TON) of of oxy. products (oxy. p., in $\frac{g}{kg \times day}$), n-alkanes (n-alk, in $\frac{g}{kg \times day}$), *iso*-alkanes (*iso*-alk, in $\frac{g}{kg \times day}$) and the total TON of all detected products (Σ, in $\frac{g}{kg \times day}$).

catalyst			m(products)				TON				for more
metal source	mineral	m(Met.) [‰]	oxy. P. [μg]	n-alk [μg]	*iso*-alk [μg]	Σ [μg]	oxy. P. [$\frac{g}{kg \times day}$]	n-alk [$\frac{g}{kg \times day}$]	*iso*-alk [$\frac{g}{kg \times day}$]	Σ [$\frac{g}{kg \times day}$]	information see
va	olivine	8.9	96	1	-	97	5.07	0.03	0.03	5.13	p. S76
Cdc	olivine	54.7	2	3	2	7	0.01	0.02	0.01	0.05	p. S52
Mn	olivine	32.3	1	1	-	2	0.01	0.02	0.01	0.04	p. S66
Gg	olivine	15.6	-	-	-	1	0.01	0.01	-	0.02	p. S76
Cdc	olivine (Syn.)	6.0	379	1	-	380	8.26	0.04	-	8.30	p. S84
Cdc	calcium carb.	5.0	58	-	-	58	0.28	-	-	0.28	p. S85
Cdc	hydroxy ap.	34.3	151	-	-	151	1.00	-	-	1.00	p. S86
Va	hydroxy ap.	8.2	-	1	1	1	-	0.07	0.05	0.12	p. S74
Gg	hydroxy ap.	13.3	1	-	-	1	0.03	-	-	0.03	p. S78
Mn	hydroxy ap.	31.3	-	-	-	-	-	-	-	-	p. S65

B. Screening of the particle size

To verify the size of metal source particles, catalysts were prepared from montmorillonite and silica gel, respectively, with different metal concentrations of *Campo del Cielo*. The smaller the metal concentration, the smaller the nanoparticles. These catalysts, were now used in CO_2 fixation under standard conditions (T = 300 °C, p = 45 bar, H_2:CO_2 = 2:1, t = 3-4). The results are summarized in table S10.

Table S10. Results of the screening of nanoparticle size catalysts using the prepared materials: supports, their metal concentration [m(metal), in %] and their resulting particle size (∅ in nm) and the masses of oxygenated products (oxy. p. in mg), *n*-alkanes (*n*-alk, in mg), *iso*-alkanes (*iso*-alk, in mg) and the total mass of all detected products (Σ, in mg) as well as the turnover number (TON) of of oxygenated products (oxy. p., in $\frac{g}{kg \times day}$), *n*-alkanes (*n*-alk, in $\frac{g}{kg \times day}$), *iso*-alkanes (*iso*-alk, in $\frac{g}{kg \times day}$) and the total TON of all detected products (Σ, in $\frac{g}{kg \times day}$).

catalyst			m(products)				TON				for more
minerals	m(metal) [%]	∅ size [nm]	oxy.p. [μg]	*n*-alk [μg]	*iso*-alk [μg]	Σ(m) [μg]	oxy. p. $[\frac{g}{kg \times day}]$	*n*-alk $[\frac{g}{kg \times day}]$	*iso*-alk $[\frac{g}{kg \times day}]$	Σ(TON) $[\frac{g}{kg \times day}]$	inormation see
Mont.	22.79	9.21	1626	15	-	1642	1.68	0.02	-	1.69	p. S60
Mont.	11.02	6.97	1588	2	-	1590	4.32	0.01	-	4.33	p. S42
Mont.	7.39	5.50	682	152	120	953	2.84	0.63	0.50	3.97	p. S80
Silica gel	21.64	13.38	1424	14	-	1438	1.36	0.01	-	1.37	p. S40
Silica gel	12.55	11.81	2408	28	1	2437	4.34	0.05	0.002	4.39	p. S45
Silica gel	7.35	9.63	5365	118	55	5539	35.81	0.79	0.37	36.97	p. S56

To verify the micrometer size, catalysts were prepared from all supports, with different metal concentrations of *Campo del Cielo* and volcanic ash, respectively. These catalysts, were now used in CO_2 fixation under standard conditions (T = 300 °C, p = 45 bar, H_2:CO_2 = 2:1, t = 3-4). The results are summarized in table S11.

Table S11. Results of the screening of micrometer size catalysts using the prepared catalyst with the metal source: *Campo del Cielo* (Cdc) and volcanic ash (va), the supports: Diopsid, olivine, silica gel montmorillonite (mont.), hydroxy apatite (hydroxy ap.) and the metal concetration [m(metal) in %]. The masses of oxygenated products (oxy. p., in mg), *n*-alkanes (*n*-alk, in mg), *iso*-alkanes (*iso*-alk, in mg) and the total mass of all detected products (Σ, in mg) as well as the turnover number (TON) of of oxygenated products (oxy. p., in $\frac{g}{kg \times day}$), *n*-alkanes (*n*-alk, in $\frac{g}{kg \times day}$), *iso*-alkanes (*iso*-alk, in $\frac{g}{kg \times day}$) and the total TON of all detected products (Σ, in $\frac{g}{kg \times day}$).

catalyst			m(products)				TON				for more
metal source	minerals	m(metal) [‰]	oxy. p. [μg]	*n*-alk [μg]	*iso*-alk [μg]	Σ(m) [μg]	oxy. p. [$\frac{g}{kg \times day}$]	*n*-alk [$\frac{g}{kg \times day}$]	*iso*-alk [$\frac{g}{kg \times day}$]	Σ(TON) [$\frac{g}{kg \times day}$]	information see
Cdc	diopsid	3.0	51	-	-	51	8.25	0.04	-	8.29	p. S84
Cdc	olivine	3.8	-	-	-	-	-	-	-	-	p. S70
Cdc	silica gel	9.0	159	2	1	162	8.81	0.11	0.08	8.99	p. S78
Cdc	silica gel	5.0	8	-	-	8	0.167	-	-	0.167	p. S86
Cdc	mont.	8.4	11	1	-	11	0.55	0.04	-	0.59	p. S61
Cdc	hydroxy ap.	22.8	3	-	-	3	0.06	0.01	-	0.07	p. S70
Va	diopsid	3.9	41	3	2	46	7.70	0.57	0.28	8.55	p. S66
Va	olivine	4.7	1	-	-	1	0.15	-	-	0.15	p. S67
Va	silica gel	2.8	8	-	-	8	1.47	0.01	-	1.48	p. S69
Va	monti	3.0	-	1	1	2	-	0.08	0.08	0.15	p. S68
Va	hydroxy ap.	9.0	7	-	-	8	0.28	0.01	0.01	0.31	p. S68

C. Temperature

For the temperature and reaction time screening we were used various temperatures (150-300 °C) and reaction times (3-38 d) in CO_2 fixation under conditions (p = 45 bar, H_2:CO_2 = 2:1, catalyst = synthetic *Campo del Cielo*@montmorillonite). The results are summarized in table S12.

Table S12. Results of the screening of temperature (T, °C) and reaction time (t, d). The masses of oxygenated products (oxy. p., in mg), *n*-alkanes (*n*-alk, in mg), *iso*-alkanes (*iso*-alk, in mg) and the total mass of all detected products (Σ, in mg) as well as the turnover number (TON) of of oxygenated products (oxy. p., in $\frac{g}{kg \times day}$), *n*-alkanes (*n*-alk, in $\frac{g}{kg \times day}$), *iso*-alkanes (*iso*-alk, in $\frac{g}{kg \times day}$) and the total TON of all detected products (Σ, in $\frac{g}{kg \times day}$). In all reactions were used synthetic *Campo del Cielo*@montmorillonite with 45 bar and ratio of H_2:CO_2 2:1.

conditions		m(products)				TON				for more
t	T	oxy. p.	*n*-alk	*iso*-alk	Σ(m)	oxy. p.	*n*-alk	*iso*-alk	Σ(TON)	information
d	[°C]	[μg]			[μg]	[$\frac{g}{kg \times day}$]			[$\frac{g}{kg \times day}$]	see
3	300	682	152	120	953	2.84	0.63	0.50	3.97	p. S80
8	300	887	6	-	893	1.43	0.01	-	1.44	p. S46
38	300	438	60	127	625	0.15	0.02	0.04	0.21	p. S48
7	250	437	3	-	440	0.77	-	-	0.77	p. S44
7	200	258	3	-	261	0.45	0.01	-	0.46	p. S44
17	170	2280	4	-	2283	1.79	-	-	1.79	p. S49
14	150	934	10	4	948	0.86	0.01	-	0.88	p. S50

D. Pressure

For the pressure and partial pressure screening we were used various pressures (9-45 bar) and ratios of H_2:CO_2 (1:9 to 9:1) in CO_2 fixation under conditions (T = 300 °C, t = 3-4, catalyst = synthetic *Campo del Cielo*@montmorillonite). The results are summarized in table S13.

Table S13. Results of the various pressures (p, in bar), partial pressure of CO_2 [p(CO_2)] and hydrogen pressure [p(H_2)]. The masses of oxygenated products (oxy. p., in mg), *n*-alkanes (*n*-alk, in mg), *iso*-alkanes (*iso*-alk, in mg) and the total mass of all detected products (Σ, in mg) as well as the turnover number (TON) of oxygenated products (oxy. p., in $\frac{g}{kg \times day}$), *n*-alkanes (*n*-alk, in $\frac{g}{kg \times day}$), *iso*-alkanes (*iso*-alk, in $\frac{g}{kg \times day}$) and the total TON of all detected products (Σ, in $\frac{g}{kg \times day}$). In all reactions were used synthetic *Campo del Cielo*@montmorillonite at 300 °C for 3-4 d.

conditions			m(products)				TON				for more
p(H_2) bar	p(CO_2) [bar]	p [bar]	oxy. p. [μg]	*n*-alk [μg]	*iso*-alk [μg]	Σ(m) [μg]	oxy. p. [$\frac{g}{kg \times day}$]	*n*-alk [$\frac{g}{kg \times day}$]	*iso*-alk [$\frac{g}{kg \times day}$]	Σ(TON) [$\frac{g}{kg \times day}$]	information see
6	3	9	33	12	16	61	0.14	0.05	0.07	0.27	p. S54
14	7	21	9	9	9	27	0.04	0.04	0.04	0.12	p. S39
23	12	35	209	23	19	252	1.29	0.14	0.12	1.55	p. S52
30	15	45	682	152	120	953	1.75	0.05	0.06	1.86	p. S80
5	40	45	517	20	38	575	3.34	0.13	0.25	3.72	p. S39
24	21	45	1611	58	44	1713	6.87	0.25	0.19	7.30	p. S41
22.5	22.5	45	656	21	9	686	4.35	0.14	0.06	4.54	p. S46
28	17	45	1250	22	3	1275	5.15	0.09	0.01	5.26	p. S47
21	24	45	727	15	13	755	4.73	0.10	0.08	4.91	p. S53
19	26	45	746	57	30	834	4.99	0.38	0.20	5.57	p. S54
15	30	45	406	13	14	433	1.75	0.05	0.06	1.86	p. S56
27	19	46	846	35	25	906	5.37	0.22	0.16	5.75	p. S42
41	5	46	34	12	4	51	0.11	0.04	0.01	0.16	p. S50

The results of the experiments with water instead of hydrogen are summarized in table S14. The conditions were T = 300 °C, t = 3-4, catalyst = synthetic *Campo del Cielo*@montmorillonite and pressure of CO_2 = 40 bar

Table S14. Results of the screening of water instead of hydrogen. The masses of oxygenated products (oxy. p., in mg), *n*-alkanes (*n*-alk, in mg), and the total mass of all detected products (Σ, in mg) as well as the turnover number (TON) of of oxygenated products (oxy. p., in $\frac{g}{kg \times day}$), *n*-alkanes (*n*-alk, in $\frac{g}{kg \times day}$), and the total TON of all detected products (Σ, in $\frac{g}{kg \times day}$). In all reactions were used synthetic *Campo del Cielo*@montmorillonite at at 300 °C for 3-4 d. *Iso*-alkanes were not detected

conditions	m(products)			TON			for more
V(water) mL	oxygenated p. [μg]	*n*-alk [μg]	Σ(m) [μg]	oxy. p. [$\frac{g}{kg \times day}$]	*n*-alk [$\frac{g}{kg \times day}$]	Σ(TON) [$\frac{g}{kg \times day}$]	information see
0.1	3	-	3	0.008	-	0.008	p. S59
0.2	23	-	23	0.074	-	0.074	p. S48
1.0	82	2	84	0.117	0.003	0.120	p. S51

VIII. MASS ESTIMATE OF PREBIOTIC ORGANICS SYNTHESIZED ON THE EARLY EARTH

A. Paleo-geological and environmental conditions

In this section, we provide a ballpark estimate of how much prebiotic organics could have been produced by the proposed scenario under the conditions representing the Hadean Earth. The major hindrance and the source of considerable uncertainties in this analysis lies in the paucity of geological and fossil records from this nascent epoch [5, 6]. The radiogenic dating of primitive meteorites and dynamical modeling of the early Solar System have shown that the main accretion phase for the Earth could have lasted about ~ 10 million years (Myr), followed by intense bombardment by planetesimals, including a Moon-forming collision at $\sim 30 - 50$ Myr after its formation [7–9].

After the Moon-forming impact, the Earth's crust has become molten, and the early

Earth has been covered by a global magma ocean with a dense ($\gtrsim 10 - 100$ bar) and hot ($\gtrsim 1\,000$ K), H_2- and silicate-rich atmosphere [8, 10, 11]. The rotational period of the Earth at that epoch has been only $\sim 2.2 - 4$ hours [12], leading to stronger wind gradients and atmospheric dynamics than nowadays. These harsh conditions unfavorable for the origin of life should have rapidly become more clement, as detrital zircon crystals provided evidence for the existence of stable continental crust and hydrosphere on the early Earth already at ~ 4.4 Ga, only $\lesssim 50 - 100$ Myr after the Moon-forming impact [13, 14].

At that stage, the early Earth's atmosphere has become cooler, $\lesssim 150 - 300$ C and CO_2-rich due to the H_2 thermal escape and volatile outgassing during the solidification of the magma ocean [15–21]. The water vapor has condensed out from the atmosphere and formed a hot pristine ocean with twice the volume of the modern hydrosphere, where the subaerial surface has likely been dominated by the volcanoes and volcanic islands [8, 22, 23]. The subaerial surface area has been much smaller than today, covering at most $S_{\rm land} \lesssim 0.1 - 1\%$ of the entire Earth' surface (which corresponds to $\lesssim 5 \times 10^5 - 5 \times 10^6$ km^2) [23, 24]. The conditions on the Hadean Earth at $\sim 4 - 4.4$ Ga could have been the most favorable for the production of prebiotic organics via our catalytic synthesis, as it requires $T \gtrsim 150 - 200$ C at the surface and a high flux of iron(-rich) catalytic particles. The hyperthermophilic organisms proliferate at temperatures up to 122 C [25], which is not that far from the temperature conditions relevant for our synthesis.

B. Delivery rate of exogenous meteoritic particles

According to the lunar cratering records, the initial intense, sterilizing bombardment of the Earth at ~ 4.5 has been followed by a steady decline in the impactor's flux, potentially with another intense, $\sim 10 - 100$ Myr period of the Late Heavy Bombardment (LHB) at $\sim 3.8 - 3.9$ Ga [26–31]. The exogenous delivery has likely been dominated by massive, $\gtrsim 10 -\ 000$ km asteroids from the inner terrestrial planet zone [32–35]. The size-number statistics for the late veneer' carriers suggests a range of possibilities, from a single delivery of the highly siderophile elements via a Pluto-sized object (with a radius of $\gtrsim 1\,000$ km) to a multitude ($> 10 - 100$) of smaller impacts by $\sim 100 - 200$ km asteroids [21]. In the single giant impact scenario, the oceans would evaporate and full reduction of all water and CO_2 would occur, leading to a H_2-rich atmosphere and efficient in situ production of organics

via Fischer-Tropsch and Urey-Miller syntheses [36]. In contrast, in the scenario of multiple smaller impacts, the oceans wouldn't vaporize, and the atmosphere will remain CO_2-rich and H_2-poor.

Planetesimals with sizes $\gtrsim 20$ km that formed within ~ 2.7 Myr after the formation of the Ca–Al-rich inclusions (CAIs) have become differentiated due to the radiogenic heating by the short-lived radionuclides [37]. A fraction of these differentiated planetesimals shattered by planetesimal collisions has formed a population of iron cores like 16 Psyche, as well as smaller metallic fragments and particles [38, 39]. The relative abundance of these iron-rich cores could have been at least $\sim 10\%$ of the total impactor's population [40]. Another exogenous source of metals on the early Earth has been partly differentiated stony and stony-iron planetesimals similar to the S-type main-belt asteroids. These siliceous planetesimals have contained up to ~ 10 wt% of metallic iron [41], and had a relative abundance of $\gtrsim 20\%$. The most primitive carbonaceous chondrite planetesimals, similar in composition to the C-type meteorites, had retained $\sim 1 - 15$ wt% of metals and metal oxides, and hence could have been an exogenous source of both the metallic and iron-rich silicate particles [42–44].

We use the early bombardment model for the "late veneer" from Pearce et al. [24]. It has a linearly declining exogenous delivery rate, with a minimum and maximum values of $\sim 3 \times 10^{12}$ and 2×10^{15} kg yr^{-1} at 4.4 Ga. According to this bombardment model, an impactor with a radius of 10-100 km would fall on the Hadean Earth on average once per $\lesssim 10^3 - 3 \times 10^6$ years [24, 45]. Over a timespan of $\gtrsim 10 - 100$ Myr, at least several large impacts could occur [46]. Impacts by the asteroids with diameters $\lesssim 100$ km would not have been energetic enough ($< 10^{28}$ J) to vaporize the early Earth's pristine ocean and to erode its surface, favoring the early origin and evolution of life [31, 47–51].

Only nanometer and micrometer-sized metallic and iron-rich silicate particles are important for the proposed organic synthesis. We consider the following main exogenous sources of such particles: 1) (sub)micron-sized iron and iron-rich interplanetary dust particles (IDPs) landing mainly intact, 2) ablation and evaporation of the iron and stony meteorites (mm- to meter-sized), and 3) evaporative impacts of $\gtrsim 1 - 100$ km-sized planetesimals [52–54]. Since the exogenous delivery was dominated by large asteroids [48], the evaporative impacts have likely been the most important exogenous source of the catalytic particles.

Assuming that the fraction of the iron(-rich) IDPs has been $\sim 40\%$ as nowadays ($\sim 10\%$ iron-rich silicates and 30% iron-sulfur-nickel) [55], the delivery rate of such catalytically

active IDPs could have been up to $\sim 4 \times 10^9$ kg yr^{-1} at 4.4 Ga [52]. The delivery rate of the nano- and micron-sized iron(-rich) particles produced by the ablation, evaporation or airbursts of meteorites and asteroids is harder to estimate. Ablation leads to the removal of $\gtrsim 3 - 25\%$ of the mass from the infalling $\lesssim 100$ kg meteorites (with typical diameters of $\lesssim 20$ cm), depending on their atmospheric entry velocity, angle, composition and cross-section [56]. Slow meter-sized and larger meteorites lose only $\lesssim 1\%$ of the initial mass by ablation, but $\gtrsim 60 - 99\%$ of their mass is lost by subsequent evaporation after the impact [43, 56, 57]. *Ab initio* simulations of the evaporative impacts for larger iron cores have shown that their peak temperatures could reach 15 000 K, and up to 22 wt% of the body could be vaporized, depending on the composition and the entry angle, collision velocity, etc. [58]. An FeNi impactor with a radius of 5 km and an impact velocity of 15 km s^{-1} would produce about 3.5 times its mass as the vapor plume [59]. Impactors with radii > 100 km could produce a mass of the vapor plume comparable with that of the modern atmosphere [46].

In the CO_2-rich atmosphere, post-impact gases would cool and condense out at $\sim 1\,700$ K as metals, metal oxides and silicates on a timescale of less than a few months [46, 47, 60, 61]. Condensates are also launched from the impact zone to space and reenter the atmosphere all around the globe, undergoing secondary heating and ablation [59]. The iron and iron-rich condensate particles will react with atmospheric gasses, reducing atmospheric CO or CO_2 into CH_4 and other light hydrocarbons [46, 60]. Experiments has shown that such "iron-silicate smokes" can catalytically convert H_2, CO and N_2 to water, CO_2, NH_3, CH_4 with comparable efficiency to industrial catalysts [62].

Sekine et al. (2003) have estimated that a typical size of the condensed particles would be $\sim 10\mu$m-1 mm and smaller [59]. In our calculations, we assumed that the fraction of catalytically active, nanometer- to micron-sized iron and iron-rich silicate particles is $\lesssim 0.1 - 1\%$ of the evaporated mass of the impactors. We estimate that a total delivery rate of the catalytically active exogenous particles could have been up to $\sim 3 \times 10^9 - 2 \times 10^{13}$ kg yr^{-1}.

Only a fraction of catalytic particles would settle on the subaerial Earth' surface, though. Sedimentation of these nano- and (sub-)micron-sized particles in the dynamically active atmosphere, with atmospheric circulations and winds, would result in their total delivery rate onto the subaerial surface of $\dot{R}_{\rm cat}^{\rm exo} \sim 5 \times 10^7 - 4 \times 10^{11}$ kg yr^{-1}. Please bear in mind that giant impacts are stochastic and can deliver a substantial mass ($\gtrsim 10^{10}$ kg) of catalytic particles within a short time period. Even in an extreme case of a single impact by a

planetesimal with a diameter of 100 km, a representative density of 2 700 $kg\,m^{-3}$, a mass of $\approx 1.4 \times 10^{18}$ kg, the mass of the nano/micron-sized condensates would cover only $\sim 0.1-1$ cm of the Earth' surface, allowing efficient reduction of the atmospheric CO_2 and H_2.

C. Deposition rate of volcanic ash particles

The Hadean Earth has likely been more geologically active than the Earth today due to a higher internal heat, a more radiogenic mantle, and a much higher rate of the asteroid impacts [63]. It is unclear when the plate tectonics and the modern-like volcanism at the plate boundaries have commenced on the early Earth. The earliest geological records point to the Paleoarchean or an earlier era, $\gtrsim 3.2-4.2$ Ga as the onset of the plate tectonics [64–67]. Here, we conservatively assume that the Hadean Earth's geodynamics at ~ 4.4 Ga has been driven by the stagnant lid convection. The hotspot, hot plume volcanism should have created explosive volcanic chains and Large Igneous Provinces (LIPs) [23, 68–71]. Due to the much hotter mantle, the volcanic activity has likely been dominated by the lava-dominated silicic LIPs, driving explosive rhyolitic volcanism [63, 72–75].

Following Bada et al. [23], we assume that at ~ 4.4 Ga there have been at least ~ 50 active hotspot volcanic islands on Earth as nowadays. Assuming that all these islands has a similar circular area, a typical radius of the volcanic island is $\sim 50-200$ km. Using the data from Tagaris et al. (2013)[76], we further assume that about several tens of volcanoes have been erupting at any given time, with a total number of 50–70 eruptions and at least one large eruption with a Volcanic Explosivity Index (VEI) ~ 5 annually. While violent eruptions with VEI of $\gtrsim 8$ and $\gtrsim 1\,000$ km^3 of the ash deposition, similar to or larger in magnitude to the Toba eruption at 73 ka [77] cannot be ruled out, it is hard to predict how often such eruptions could have occurred on the early Earth. A too intense volcanism would have produced high amounts of atmospheric dust as well as SO_2 and H_2S aerosols, leading to a too excessive cooling of the early Earth's atmosphere [12, 78, 79]. It has been estimated from geophysical models that the volcanic activity on Hadean Earth could have been at most a few times higher than nowadays [80].

With these assumptions, the emission volume of the volcanic ash into the atmosphere would be at least $\sim 20-100$ km^3. Volcanic ash particles with diameters $\lesssim 30\mu$m would constitute a few and $\approx 30-50$ wt% of the total ash content during basaltic and rhyolitic

eruptions, respectively [76]. In our calculations, the fraction of 10 wt% is assumed. The iron-rich fraction in these particles could have been up to $\sim 30 - 50\%$, similar to the felsic ash produced by the modern rhyolite eruptions [81, 82]. We used the iron-rich fraction of 40% in our model. Hence, the total deposition rate of the catalytic iron-rich ash particles on the early Earth could have been $\sim 5 \times 10^{12} - 10^{13}$ kg yr^{-1}.

In a $\sim 1 - 10$ bar atmosphere, the volcanic ash particles with diameters $\lesssim 1 - 10 \mu$m brought up to the altitudes of $\gtrsim 1$ km would attain settling velocities of $\lesssim 0.3 - 3$ m/s or higher [83]. Despite such low settling velocities, the fine ash particles often sediment very close to the volcanoes due to gravitational instabilities occurring in the interface between the ash cloud and the surrounding atmosphere [82, 84–87]. Thus, a substantial fraction ($> 10\%$) of the catalytically active volcanic ash particles would settle on the volcanic islands, and the resulting ash deposition rate on the subaerial surface could have been $\dot{R}_{\rm cat}^{\rm vul} \sim 2 \times 10^{11} - 10^{12}$ kg yr^{-1}.

D. Mass estimate of synthesized organics

The catalytically active exogenous and volcanic particles on the early Earth should have had a diversity of size distributions, depending on the distance between the impact or eruption site and the landing region, atmospheric and meteorological conditions, a type of impact or eruption, etc. [82, 88]. Since only nanometer and (sub)micrometer-sized grains matter for the proposed organic synthesis, we assume that a majority of such small grains would have settled onto the subaerial surface after the sedimentation of bigger airborne fragments of a meteorite or an asteroid or volcanic pyroclasts.

The catalytic particles used in our experiments have typical sizes of $\sim 1 - 10$ nm when prepared by dissolution and wet impregnation, and at least 10 μm when prepared by milling (see section IV in Supplementary Information). The measured yields of the organic synthesis for micrometer-sized catalysts are lower than those of nanoparticles by at least a factor of several (see Fig. 4 in the main text).

Then, assuming a linear relationship between the yield of the organic reactions and the reaction time, the production rate of the prebiotic organics can be roughly estimated as follows:

$$\dot{R}_{\rm org} \sim (\dot{R}_{\rm cat}^{\rm vul} \gamma_{\rm cat}^{\rm vul} + \dot{R}_{\rm cat}^{\rm exo} \gamma_{\rm cat}^{\rm exo}) \Delta t \ ({\rm g\,yr}^{-1}), \qquad (1)$$

where $\dot{R}_{\rm cat}^{\rm vul}$ and $\dot{R}_{\rm cat}^{\rm exo}$ are the volcanic and exogenous delivery rates of the catalytic particles ($\rm kg\,yr^{-1}$), respectively, $\gamma_{\rm cat}^{\rm vul}$ and $\gamma_{\rm cat}^{\rm exo}$ are the yields of the organic synthesis for the volcanic and exogenous catalytic particles (in units of g of synthesized organics per kg of catalytic particles per day), respectively, and Δt describes how long catalytic particles remain active (days).

We assume a 1 bar of the N_2 pressure and adopt the CO_2 and H_2 atmospheric pressures from the model of Zahnle et al. (2020)[21] (see their Table 1) for the scenarios when the exogenous delivery would have been dominated by 3-20 ("Sub-Vesta") or 10-100 ("South Pole–Aitken Moon basin") impacts. In the first scenario, the $P(CO_2) = P(H_2) = 2.7$ bar. In the second scenario, $P(CO_2) = 1.32$ bar and $P(H_2) = 0.65$ bar. We used 130-200 C as the surface temperature of the volcanic islands, heated either via geological activity or by the atmosphere if the geological removal of greenhouse gases was slow [69, 89, 90].

We also assume that the minerals on the subaerial surface have resembled montmorillonite, a a typical product of the weathering of volcanic rocks at the acidic conditions representative of the early Earth [91–93]. The measured production yields for the volcanic and *Campo del Cielo* catalytic particles on the montmorillonite support have been rescaled to the assumed temperatures and pressures. Based on the results of our experiments, we assume that the catalytic particles landed on these minerals would remain active for ~ 30 days. Finally, using the above assumptions and values, and the delivery rate from the Eq. 1, the total production rate of the prebiotic organics by the volcanic and exogenous particles is estimated to be $\sim 10^6 - 6 \times 10^8$ $\rm kg\,yr^{-1}$ (depending on the atmospheric temperature and pressure, bombardment rate, and the volcanic activity). Thus, the total mass of the synthesized organics on Hadean Earth could have been $\sim 10^{13} - 6 \times 10^{15}$ kg over a ~ 10 Myr or $\sim 10^{14} - 6 \times 10^{16}$ kg over a ~ 100 Myr, respectively.

IX. REACTIONS

Reaction 1: Campo del Cielo @ Montmorillonite

Table S15. Reaction conditions and masses of the entire catalysts (Cat) and its metal share (Met).

Synthesis	m(Cat) [g]	m(Met) [g]	t [d]	T [°C]	$p(H_2)$ [bar]	$p(CO_2)$ [bar]
Nano Particles	1.005	0.077	2	300	5.0	40.0

Table S16. Masses (m) and turnover numbers (TON) of the products: Oxygenated products (Oxy), alkanes (Alk), *iso*-alkanes (Iso), and their sum (Tot).

	Oxy	Alk	Iso	Tot
m [mg]	0.517	0.020	0.038	0.575
TON [g/(kg d)]	3.343	0.130	0.245	3.719

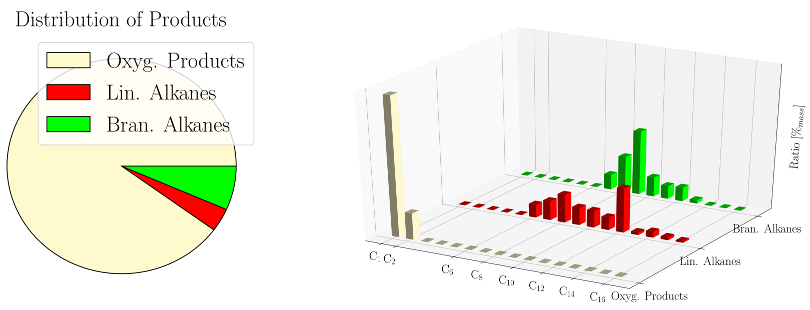

Figure S16. Distribution of products in the liquid phase.

Reaction 2: Campo del Cielo @ Montmorillonite

Table S17. Reaction conditions and masses of the entire catalysts (Cat) and its metal share (Met).

Synthesis	m(Cat) [g]	m(Met) [g]	t [d]	T [°C]	$p(H_2)$ [bar]	$p(CO_2)$ [bar]
Nano Particles	1.020	0.078	3	300	14.0	7.0

Table S18. Masses (m) and turnover numbers (TON) of the products: Oxygenated products (Oxy), alkanes (Alk), *iso*-alkanes (Iso), and their sum (Tot).

	Oxy	Alk	Iso	Tot
m [mg]	0.009	0.009	0.009	0.027
TON [g/(kg d)]	0.040	0.036	0.037	0.114

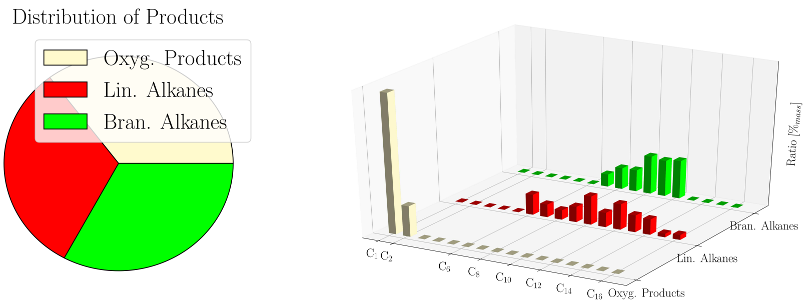

Figure S17. Distribution of products in the liquid phase.

Reaction 3: Campo del Cielo @ Silica Gel

Table S19. Reaction conditions and masses of the entire catalysts (Cat) and its metal share (Met).

Synthesis	m(Cat) [g]	m(Met) [g]	t [d]	T [°C]	p(H_2) [bar]	p(CO_2) [bar]
blank	0.541	0.046	1	300	50.0	0.0

Table S20. Masses (m) and turnover numbers (TON) of the products: Oxygenated products (Oxy), alkanes (Alk), *iso*-alkanes (Iso), and their sum (Tot).

	Oxy	Alk	Iso	Tot
m [mg]	0.001	0.000	0.000	0.001
TON [g/(kg d)]	0.011	0.000	0.004	0.015

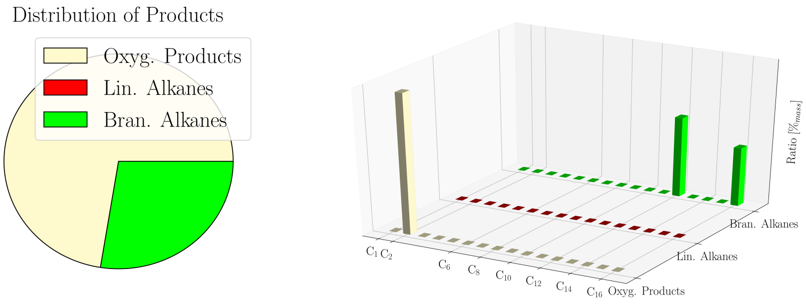

Figure S18. Distribution of products in the liquid phase.

Reaction 4: Campo del Cielo @ Silica Gel

Table S21. Reaction conditions and masses of the entire catalysts (Cat) and its metal share (Met).

Synthesis	m(Cat) [g]	m(Met) [g]	t [d]	T [°C]	p(H_2) [bar]	p(CO_2) [bar]
Nano Particles	1.100	0.262	4	300	30.0	15.0

Table S22. Masses (m) and turnover numbers (TON) of the products: Oxygenated products (Oxy), alkanes (Alk), *iso*-alkanes (Iso), and their sum (Tot).

	Oxy	Alk	Iso	Tot
m [mg]	1.424	0.014	0.000	1.438
TON [g/(kg d)]	1.360	0.013	0.000	1.373

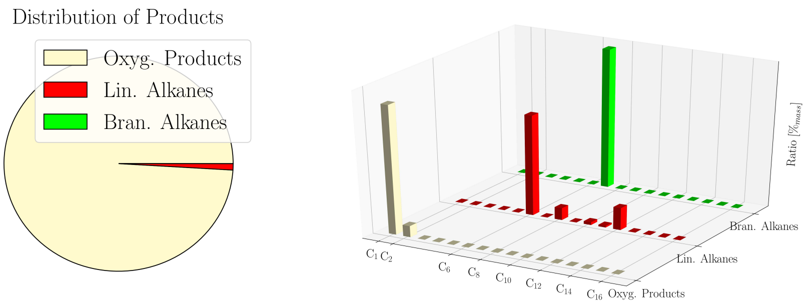

Figure S19. Distribution of products in the liquid phase.

Reaction 5: Campo del Cielo @ Montmorillonite

Table S23. Reaction conditions and masses of the entire catalysts (Cat) and its metal share (Met).

Synthesis	m(Cat) [g]	m(Met) [g]	t [d]	T [°C]	p(H_2) [bar]	p(CO_2) [bar]
Nano Particles	1.017	0.078	3	300	24.0	21.0

Table S24. Masses (m) and turnover numbers (TON) of the products: Oxygenated products (Oxy), alkanes (Alk), *iso*-alkanes (Iso), and their sum (Tot).

	Oxy	Alk	Iso	Tot
m [mg]	1.611	0.058	0.044	1.713
TON [g/(kg d)]	6.870	0.248	0.187	7.305

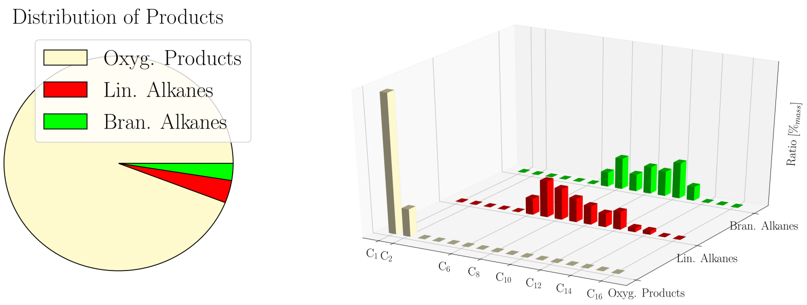

Figure S20. Distribution of products in the liquid phase.

Reaction 6: Campo del Cielo @ Montmorillonite

Table S25. Reaction conditions and masses of the entire catalysts (Cat) and its metal share (Met).

Synthesis	m(Cat) [g]	m(Met) [g]	t [d]	T [°C]	$p(H_2)$ [bar]	$p(CO_2)$ [bar]
Nano Particles	1.053	0.122	3	300	30.0	15.0

Table S26. Masses (m) and turnover numbers (TON) of the products: Oxygenated products (Oxy), alkanes (Alk), *iso*-alkanes (Iso), and their sum (Tot).

	Oxy	Alk	Iso	Tot
m [mg]	1.588	0.002	-0.000	1.590
TON [g/(kg d)]	4.321	0.006	0.000	4.327

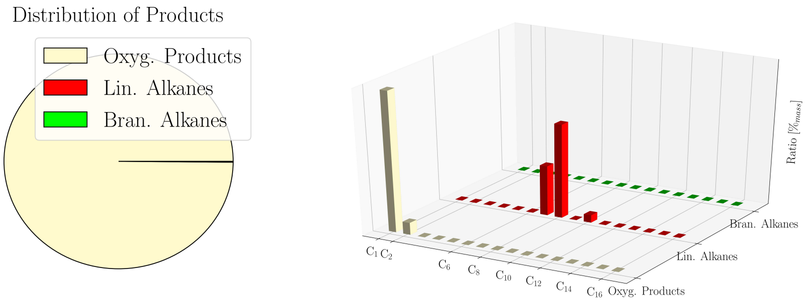

Figure S21. Distribution of products in the liquid phase.

Reaction 7: Campo del Cielo @ Montmorillonite

Table S27. Reaction conditions and masses of the entire catalysts (Cat) and its metal share (Met).

Synthesis	m(Cat) [g]	m(Met) [g]	t [d]	T [°C]	$p(H_2)$ [bar]	$p(CO_2)$ [bar]
Nano Particles	1.025	0.079	2	300	27.0	19.0

Table S28. Masses (m) and turnover numbers (TON) of the products: Oxygenated products (Oxy), alkanes (Alk), *iso*-alkanes (Iso), and their sum (Tot).

	Oxy	Alk	Iso	Tot
m [mg]	0.846	0.035	0.025	0.906
TON [g/(kg d)]	5.367	0.224	0.160	5.751

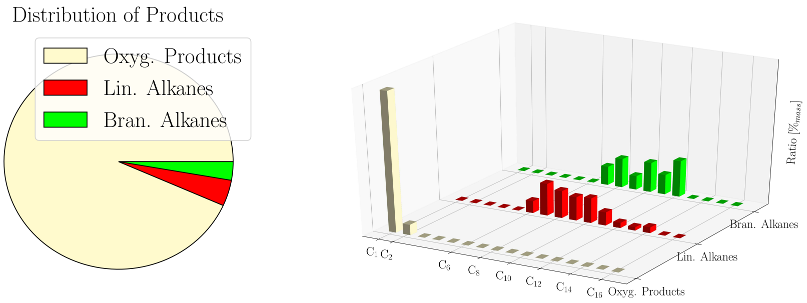

Figure S22. Distribution of products in the liquid phase.

Reaction 8: Campo del Cielo @ Montmorillonite

Table S29. Reaction conditions and masses of the entire catalysts (Cat) and its metal share (Met).

Synthesis	m(Cat) [g]	m(Met) [g]	t [d]	T [°C]	$p(H_2)$ [bar]	$p(CO_2)$ [bar]
Nano Particles	1.031	0.001	3	300	30.0	15.0

Table S30. Masses (m) and turnover numbers (TON) of the products: Oxygenated products (Oxy), alkanes (Alk), *iso*-alkanes (Iso), and their sum (Tot).

	Oxy	Alk	Iso	Tot
m [mg]	0.000	0.000	0.000	0.000
TON [g/(kg d)]	0.215	0.000	0.000	0.215

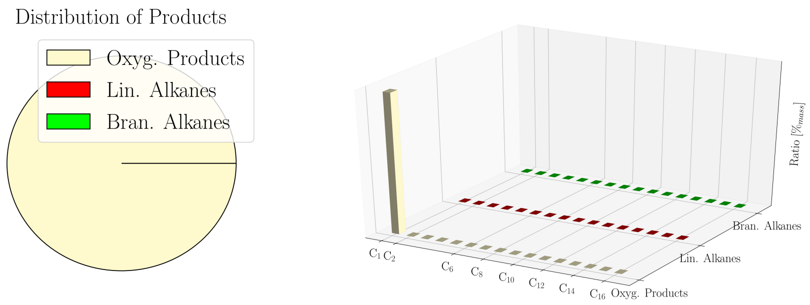

Figure S23. Distribution of products in the liquid phase.

Reaction 9: Campo del Cielo @ Montmorillonite

Table S31. Reaction conditions and masses of the entire catalysts (Cat) and its metal share (Met).

Synthesis	m(Cat) [g]	m(Met) [g]	t [d]	T [°C]	$p(H_2)$ [bar]	$p(CO_2)$ [bar]
Nano Particles	1.059	0.081	7	250	30.0	15.0

Table S32. Masses (m) and turnover numbers (TON) of the products: Oxygenated products (Oxy), alkanes (Alk), *iso*-alkanes (Iso), and their sum (Tot).

	Oxy	Alk	Iso	Tot
m [mg]	0.437	0.003	-0.000	0.440
TON [g/(kg d)]	0.767	0.004	0.000	0.771

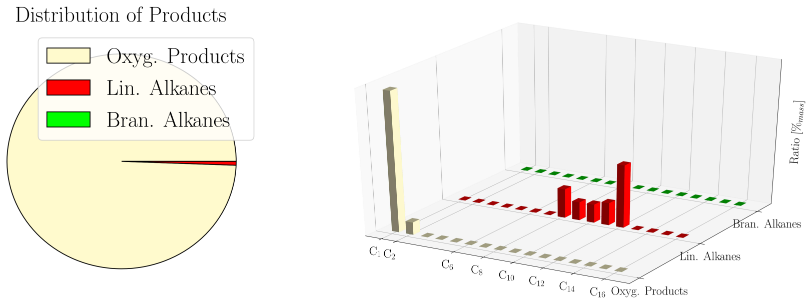

Figure S24. Distribution of products in the liquid phase.

Reaction 10: Campo del Cielo @ Montmorillonite

Table S33. Reaction conditions and masses of the entire catalysts (Cat) and its metal share (Met).

Synthesis	m(Cat) [g]	m(Met) [g]	t [d]	T [°C]	p(H_2) [bar]	p(CO_2) [bar]
Nano Particles	1.056	0.081	7	200	30.0	15.0

Table S34. Masses (m) and turnover numbers (TON) of the products: Oxygenated products (Oxy), alkanes (Alk), *iso*-alkanes (Iso), and their sum (Tot).

	Oxy	Alk	Iso	Tot
m [mg]	0.258	0.003	-0.000	0.261
TON [g/(kg d)]	0.454	0.005	0.000	0.459

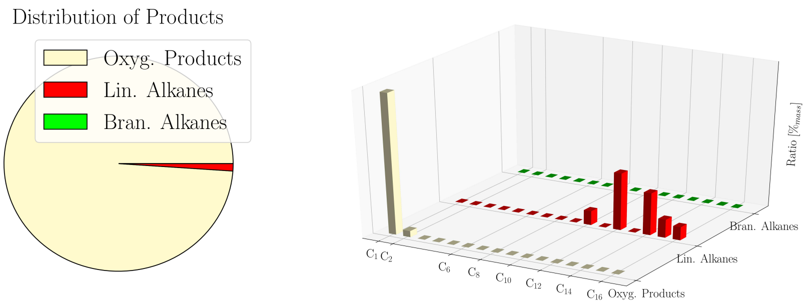

Figure S25. Distribution of products in the liquid phase.

Reaction 11: Campo del Cielo @ Silica Gel

Table S35. Reaction conditions and masses of the entire catalysts (Cat) and its metal share (Met).

Synthesis	m(Cat) [g]	m(Met) [g]	t [d]	T [°C]	p(H_2) [bar]	p(CO_2) [bar]
Nano Particles	1.076	0.145	4	300	30.0	15.0

Table S36. Masses (m) and turnover numbers (TON) of the products: Oxygenated products (Oxy), alkanes (Alk), *iso*-alkanes (Iso), and their sum (Tot).

	Oxy	Alk	Iso	Tot
m [mg]	0.758	0.000	0.000	0.758
TON [g/(kg d)]	1.304	0.000	0.000	1.304

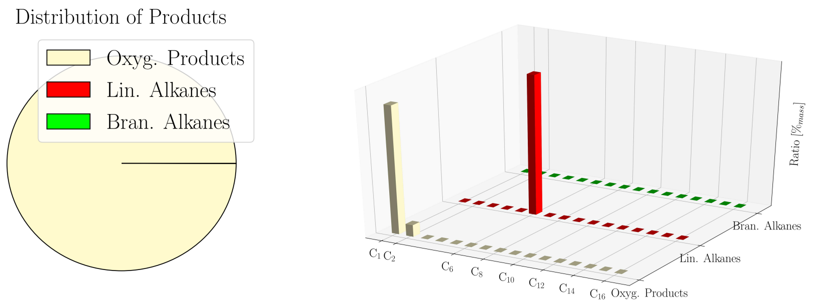

Figure S26. Distribution of products in the liquid phase.

Reaction 12: Campo del Cielo @ Montmorillonite

Table S37. Reaction conditions and masses of the entire catalysts (Cat) and its metal share (Met).

Synthesis	m(Cat) [g]	m(Met) [g]	t [d]	T [°C]	$p(H_2)$ [bar]	$p(CO_2)$ [bar]
Nano Particles	1.007	0.077	8	300	29.0	17.0

Table S38. Masses (m) and turnover numbers (TON) of the products: Oxygenated products (Oxy), alkanes (Alk), *iso*-alkanes (Iso), and their sum (Tot).

	Oxy	Alk	Iso	Tot
m [mg]	0.887	0.006	0.000	0.893
TON [g/(kg d)]	1.432	0.010	0.000	1.442

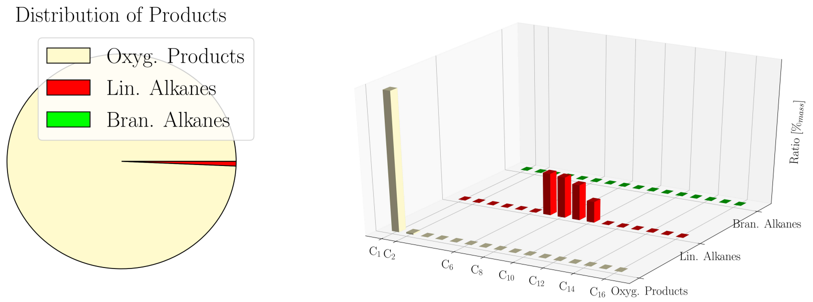

Figure S27. Distribution of products in the liquid phase.

Reaction 13: Campo del Cielo @ Montmorillonite

Table S39. Reaction conditions and masses of the entire catalysts (Cat) and its metal share (Met).

Synthesis	m(Cat) [g]	m(Met) [g]	t [d]	T [°C]	p(H_2) [bar]	p(CO_2) [bar]
Nano Particles	0.981	0.075	2	300	22.5	22.5

Table S40. Masses (m) and turnover numbers (TON) of the products: Oxygenated products (Oxy), alkanes (Alk), *iso*-alkanes (Iso), and their sum (Tot).

	Oxy	Alk	Iso	Tot
m [mg]	0.656	0.021	0.009	0.686
TON [g/(kg d)]	4.345	0.138	0.061	4.544

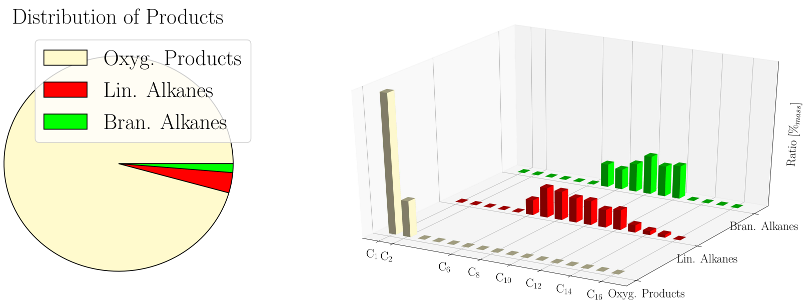

Figure S28. Distribution of products in the liquid phase.

Reaction 14: Campo del Cielo @ Montmorillonite

Table S41. Reaction conditions and masses of the entire catalysts (Cat) and its metal share (Met).

Synthesis	m(Cat) [g]	m(Met) [g]	t [d]	T [°C]	p(H_2) [bar]	p(CO_2) [bar]
Nano Particles	1.052	0.081	3	300	28.0	17.0

Table S42. Masses (m) and turnover numbers (TON) of the products: Oxygenated products (Oxy), alkanes (Alk), *iso*-alkanes (Iso), and their sum (Tot).

	Oxy	Alk	Iso	Tot
m [mg]	1.250	0.022	0.003	1.275
TON [g/(kg d)]	5.154	0.091	0.011	5.255

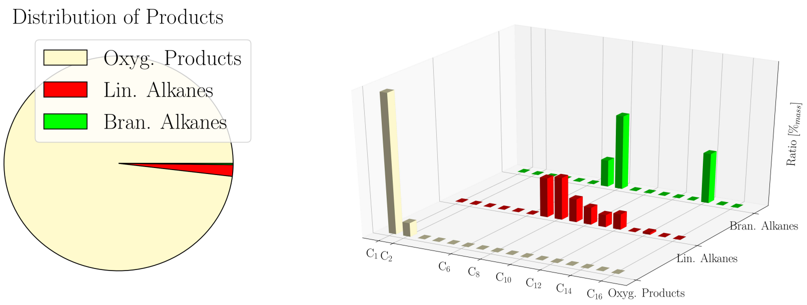

Figure S29. Distribution of products in the liquid phase.

Reaction 15: Campo del Cielo @ Montmorillonite

Table S43. Reaction conditions and masses of the entire catalysts (Cat) and its metal share (Met).

Synthesis	m(Cat) [g]	m(Met) [g]	t [d]	T [°C]	$p(H_2)$ [bar]	$p(CO_2)$ [bar]
Nano Particles	1.023	0.079	38	300	30.0	15.0

Table S44. Masses (m) and turnover numbers (TON) of the products: Oxygenated products (Oxy), alkanes (Alk), *iso*-alkanes (Iso), and their sum (Tot).

	Oxy	Alk	Iso	Tot
m [mg]	0.438	0.060	0.127	0.625
TON [g/(kg d)]	0.147	0.020	0.042	0.209

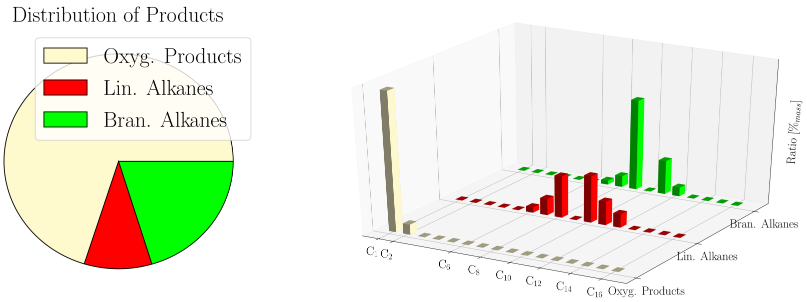

Figure S30. Distribution of products in the liquid phase.

Reaction 16: Campo del Cielo @ Montmorillonite

Table S45. Reaction conditions and masses of the entire catalysts (Cat) and its metal share (Met).

Synthesis	m(Cat) [g]	m(Met) [g]	t [d]	T [°C]	p(H_2) [bar]	p(CO_2) [bar]
Nano Particles	1.022	0.079	4	300	0.2	40.0

Table S46. Masses (m) and turnover numbers (TON) of the products: Oxygenated products (Oxy), alkanes (Alk), *iso*-alkanes (Iso), and their sum (Tot).

	Oxy	Alk	Iso	Tot
m [mg]	0.023	0.000	0.000	0.023
TON [g/(kg d)]	0.074	0.000	0.000	0.074

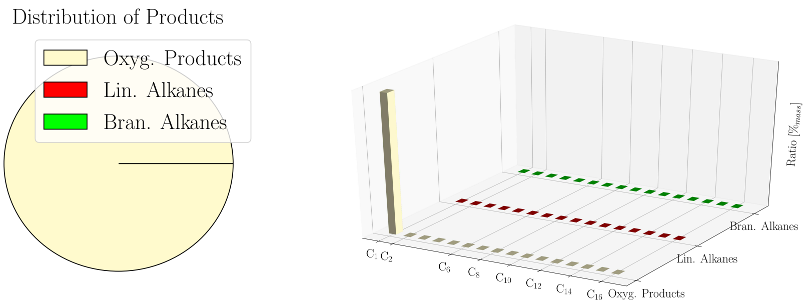

Figure S31. Distribution of products in the liquid phase.

Reaction 17: Campo del Cielo @ Montmorillonite

Table S47. Reaction conditions and masses of the entire catalysts (Cat) and its metal share (Met).

Synthesis	m(Cat) [g]	m(Met) [g]	t [d]	T [°C]	p(H_2) [bar]	p(CO_2) [bar]
Nano Particles	0.973	0.075	17	170	30.0	15.0

Table S48. Masses (m) and turnover numbers (TON) of the products: Oxygenated products (Oxy), alkanes (Alk), *iso*-alkanes (Iso), and their sum (Tot).

	Oxy	Alk	Iso	Tot
m [mg]	2.280	0.004	-0.000	2.283
TON [g/(kg d)]	1.792	0.003	-0.000	1.795

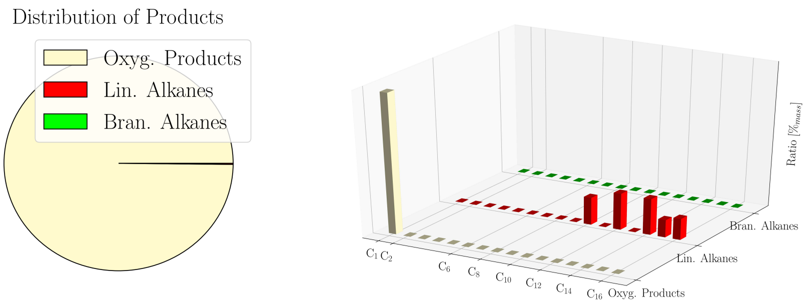

Figure S32. Distribution of products in the liquid phase.

Reaction 18: Campo del Cielo @ Montmorillonite

Table S49. Reaction conditions and masses of the entire catalysts (Cat) and its metal share (Met).

Synthesis	m(Cat) [g]	m(Met) [g]	t [d]	T [°C]	p(H_2) [bar]	p(CO_2) [bar]
Nano Particles	1.009	0.078	4	300	41.0	5.0

Table S50. Masses (m) and turnover numbers (TON) of the products: Oxygenated products (Oxy), alkanes (Alk), *iso*-alkanes (Iso), and their sum (Tot).

	Oxy	Alk	Iso	Tot
m [mg]	0.034	0.012	0.004	0.051
TON [g/(kg d)]	0.111	0.040	0.013	0.164

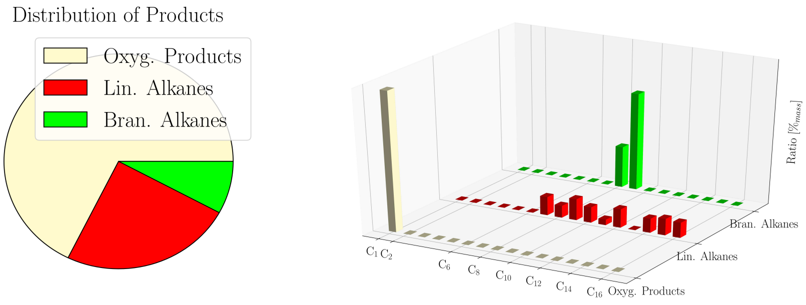

Figure S33. Distribution of products in the liquid phase.

Reaction 19: Campo del Cielo @ Montmorillonite

Table S51. Reaction conditions and masses of the entire catalysts (Cat) and its metal share (Met).

Synthesis	m(Cat) [g]	m(Met) [g]	t [d]	T [°C]	p(H_2) [bar]	p(CO_2) [bar]
Nano Particles	1.004	0.077	14	150	30.0	15.0

Table S52. Masses (m) and turnover numbers (TON) of the products: Oxygenated products (Oxy), alkanes (Alk), *iso*-alkanes (Iso), and their sum (Tot).

	Oxy	Alk	Iso	Tot
m [mg]	0.934	0.010	0.004	0.948
TON [g/(kg d)]	0.865	0.009	0.004	0.877

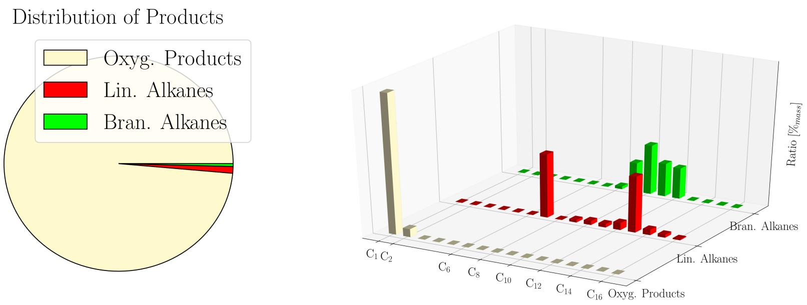

Figure S34. Distribution of products in the liquid phase.

Reaction 20: Campo del Cielo @ Olivine

Table S53. Reaction conditions and masses of the entire catalysts (Cat) and its metal share (Met).

Synthesis	m(Cat) [g]	m(Met) [g]	t [d]	T [°C]	p(H_2) [bar]	p(CO_2) [bar]
Nano Particles	1.004	0.077	6	300	10.0	40.0

Table S54. Masses (m) and turnover numbers (TON) of the products: Oxygenated products (Oxy), alkanes (Alk), *iso*-alkanes (Iso), and their sum (Tot).

	Oxy	Alk	Iso	Tot
m [mg]	0.002	0.004	-0.000	0.006
TON [g/(kg d)]	0.004	0.009	0.000	0.013

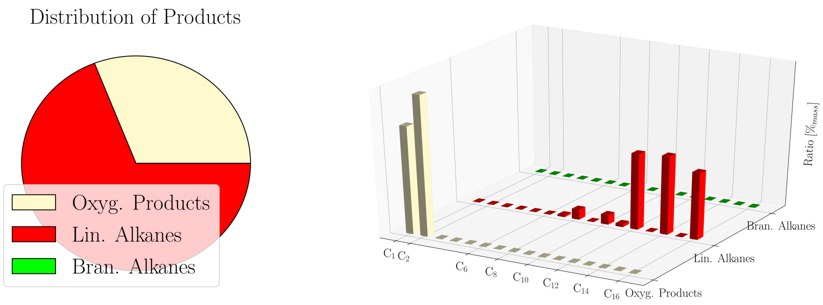

Figure S35. Distribution of products in the liquid phase.

Reaction 21: Campo del Cielo @ Olivine

Table S55. Reaction conditions and masses of the entire catalysts (Cat) and its metal share (Met).

Synthesis	m(Cat) [g]	m(Met) [g]	t [d]	T [°C]	$p(H_2)$ [bar]	$p(CO_2)$ [bar]
Nano Particles	1.151	0.073	2	300	30.0	15.0

Table S56. Masses (m) and turnover numbers (TON) of the products: Oxygenated products (Oxy), alkanes (Alk), *iso*-alkanes (Iso), and their sum (Tot).

	Oxy	Alk	Iso	Tot
m [mg]	0.002	0.003	0.002	0.007
TON [g/(kg d)]	0.015	0.021	0.011	0.047

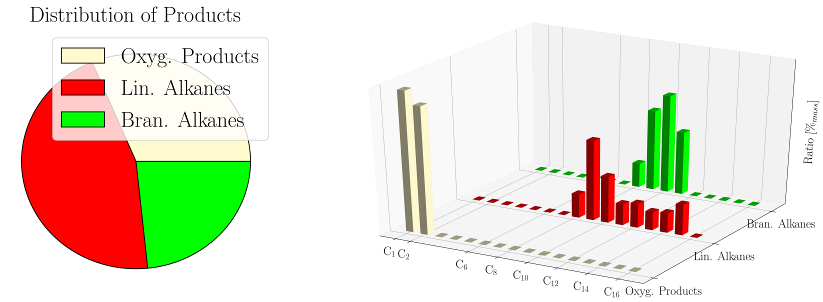

Figure S36. Distribution of products in the liquid phase.

Reaction 22: Campo del Cielo @ Montmorillonite

Table S57. Reaction conditions and masses of the entire catalysts (Cat) and its metal share (Met).

Synthesis	m(Cat) [g]	m(Met) [g]	t [d]	T [°C]	p(H_2) [bar]	p(CO_2) [bar]
Nano Particles	1.058	0.081	2	300	23.0	12.0

Table S58. Masses (m) and turnover numbers (TON) of the products: Oxygenated products (Oxy), alkanes (Alk), *iso*-alkanes (Iso), and their sum (Tot).

	Oxy	Alk	Iso	Tot
m [mg]	0.209	0.023	0.019	0.252
TON [g/(kg d)]	1.285	0.143	0.119	1.547

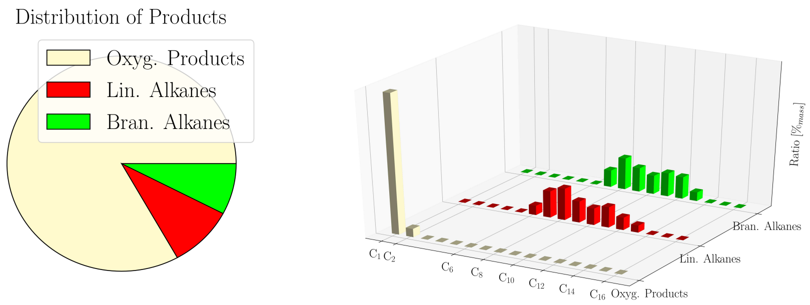

Figure S37. Distribution of products in the liquid phase.

Reaction 23: Campo del Cielo @ Montmorillonite

Table S59. Reaction conditions and masses of the entire catalysts (Cat) and its metal share (Met).

Synthesis	m(Cat) [g]	m(Met) [g]	t [d]	T [°C]	p(H_2) [bar]	p(CO_2) [bar]
Nano Particles	1.000	0.077	2	300	21.0	24.0

Table S60. Masses (m) and turnover numbers (TON) of the products: Oxygenated products (Oxy), alkanes (Alk), *iso*-alkanes (Iso), and their sum (Tot).

	Oxy	Alk	Iso	Tot
m [mg]	0.727	0.015	0.013	0.755
TON [g/(kg d)]	4.729	0.098	0.082	4.908

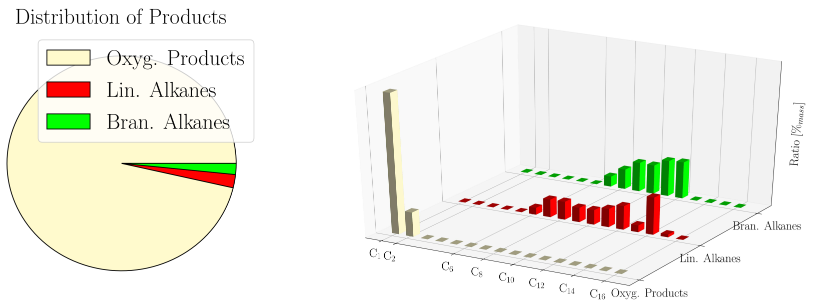

Figure S38. Distribution of products in the liquid phase.

Reaction 24: Campo del Cielo @ Montmorillonite

Table S61. Reaction conditions and masses of the entire catalysts (Cat) and its metal share (Met).

Synthesis	m(Cat) [g]	m(Met) [g]	t [d]	T [°C]	$p(H_2)$ [bar]	$p(CO_2)$ [bar]
Nano Particles	0.997	0.077	3	300	6.0	3.0

Table S62. Masses (m) and turnover numbers (TON) of the products: Oxygenated products (Oxy), alkanes (Alk), *iso*-alkanes (Iso), and their sum (Tot).

	Oxy	Alk	Iso	Tot
m [mg]	0.033	0.012	0.016	0.061
TON [g/(kg d)]	0.144	0.054	0.068	0.265

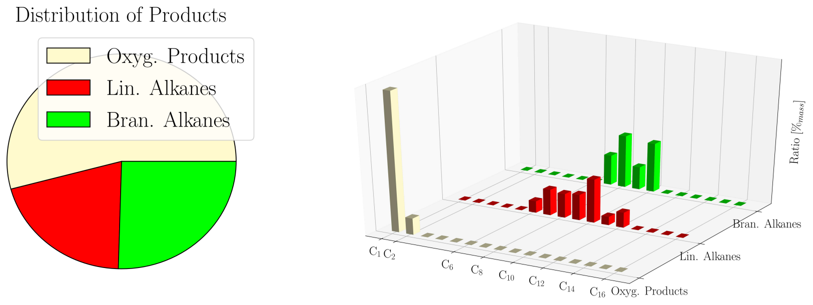

Figure S39. Distribution of products in the liquid phase.

Reaction 25: Campo del Cielo @ Montmorillonite

Table S63. Reaction conditions and masses of the entire catalysts (Cat) and its metal share (Met).

Synthesis	m(Cat) [g]	m(Met) [g]	t [d]	T [°C]	$p(H_2)$ [bar]	$p(CO_2)$ [bar]
Nano Particles	0.973	0.075	2	300	19.0	26.0

Table S64. Masses (m) and turnover numbers (TON) of the products: Oxygenated products (Oxy), alkanes (Alk), *iso*-alkanes (Iso), and their sum (Tot).

	Oxy	Alk	Iso	Tot
m [mg]	0.746	0.057	0.030	0.834
TON [g/(kg d)]	4.985	0.382	0.203	5.571

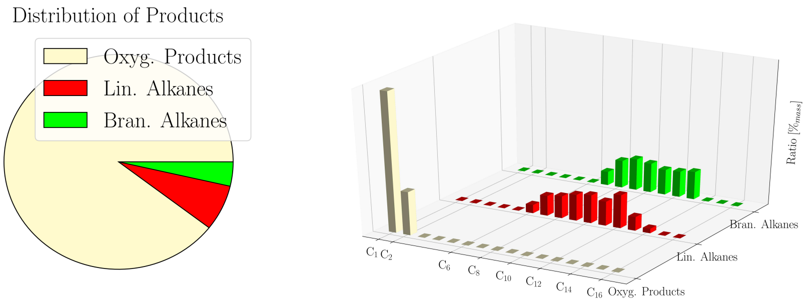

Figure S40. Distribution of products in the liquid phase.

Reaction 26: Muonionalusta @ Montmorillonite

Table S65. Reaction conditions and masses of the entire catalysts (Cat) and its metal share (Met).

Synthesis	m(Cat) [g]	m(Met) [g]	t [d]	T [°C]	$p(H_2)$ [bar]	$p(CO_2)$ [bar]
Nano Particles	1.003	0.075	2	300	30.0	15.0

Table S66. Masses (m) and turnover numbers (TON) of the products: Oxygenated products (Oxy), alkanes (Alk), *iso*-alkanes (Iso), and their sum (Tot).

	Oxy	Alk	Iso	Tot
m [mg]	0.266	0.010	0.004	0.280
TON [g/(kg d)]	1.784	0.069	0.024	1.876

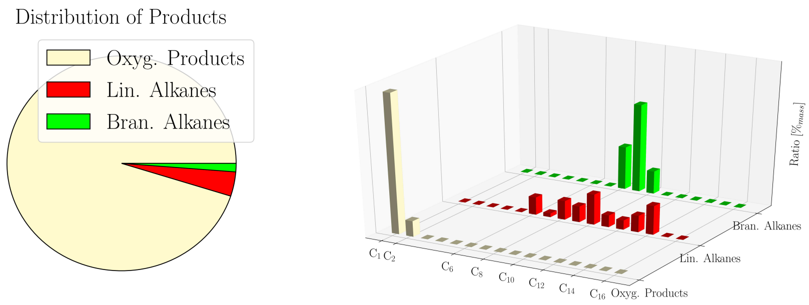

Figure S41. Distribution of products in the liquid phase.

Reaction 27: Campo del Cielo @ Silica Gel

Table S67. Reaction conditions and masses of the entire catalysts (Cat) and its metal share (Met).

Synthesis	m(Cat) [g]	m(Met) [g]	t [d]	T [°C]	$p(H_2)$ [bar]	$p(CO_2)$ [bar]
Nano Particles	1.007	0.075	2	300	30.0	15.0

Table S68. Masses (m) and turnover numbers (TON) of the products: Oxygenated products (Oxy), alkanes (Alk), *iso*-alkanes (Iso), and their sum (Tot).

	Oxy	Alk	Iso	Tot
m [mg]	5.365	0.118	0.055	5.539
TON [g/(kg d)]	35.809	0.791	0.367	36.966

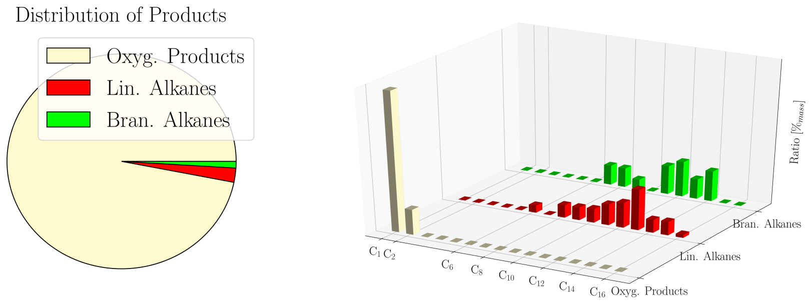

Figure S42. Distribution of products in the liquid phase.

Reaction 28: Campo del Cielo @ Montmorillonite

Table S69. Reaction conditions and masses of the entire catalysts (Cat) and its metal share (Met).

Synthesis	m(Cat) [g]	m(Met) [g]	t [d]	T [°C]	p(H_2) [bar]	p(CO_2) [bar]
Nano Particles	1.007	0.077	3	300	15.0	30.0

Table S70. Masses (m) and turnover numbers (TON) of the products: Oxygenated products (Oxy), alkanes (Alk), *iso*-alkanes (Iso), and their sum (Tot).

	Oxy	Alk	Iso	Tot
m [mg]	0.406	0.013	0.014	0.433
TON [g/(kg d)]	1.749	0.054	0.059	1.863

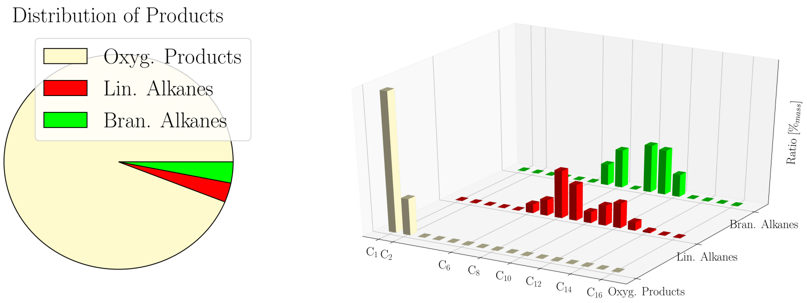

Figure S43. Distribution of products in the liquid phase.

Reaction 29: blank @ Montmorillonite

Table S71. Reaction conditions and masses of the entire catalysts (Cat) and its metal share (Met).

Synthesis	m(Cat) [g]	m(Met) [g]	t [d]	T [°C]	p(H_2) [bar]	p(CO_2) [bar]
blank	1.001	0.000	6	300	30.0	15.0

Table S72. Masses (m) and turnover numbers (TON) of the products: Oxygenated products (Oxy), alkanes (Alk), *iso*-alkanes (Iso), and their sum (Tot).

	Oxy	Alk	Iso	Tot
m [mg]	0.000	0.004	0.000	0.004
TON [g/(kg d)]	0.000	0.001	0.000	0.001

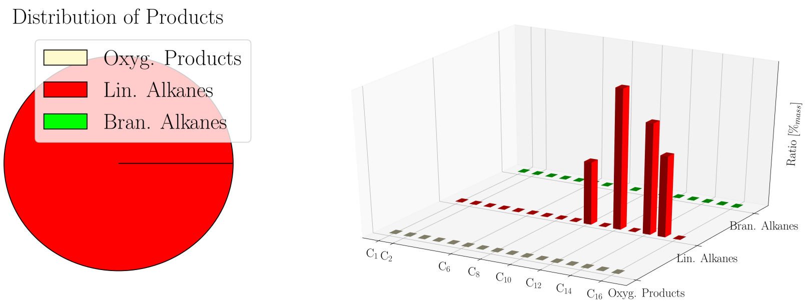

Figure S44. Distribution of products in the liquid phase.

Reaction 30: blank @ Silica Gel

Table S73. Reaction conditions and masses of the entire catalysts (Cat) and its metal share (Met).

Synthesis	m(Cat) [g]	m(Met) [g]	t [d]	T [°C]	p(H_2) [bar]	p(CO_2) [bar]
blank	1.023	0.000	6	300	30.0	15.0

Table S74. Masses (m) and turnover numbers (TON) of the products: Oxygenated products (Oxy), alkanes (Alk), *iso*-alkanes (Iso), and their sum (Tot).

	Oxy	Alk	Iso	Tot
m [mg]	0.000	0.001	0.000	0.001
TON [g/(kg d)]	0.000	0.000	0.000	0.000

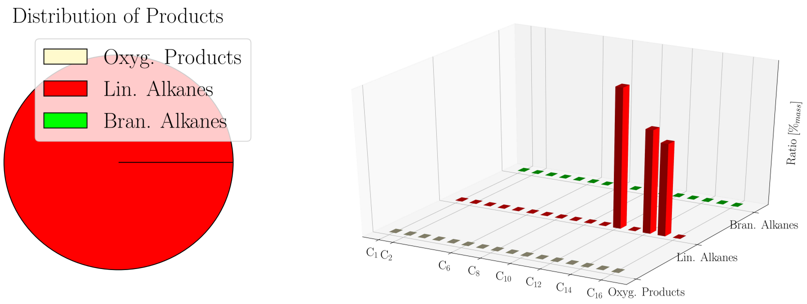

Figure S45. Distribution of products in the liquid phase.

Reaction 31: Muonionalusta @ blank

Table S75. Reaction conditions and masses of the entire catalysts (Cat) and its metal share (Met).

Synthesis	m(Cat) [g]	m(Met) [g]	t [d]	T [°C]	p(H_2) [bar]	p(CO_2) [bar]
blank	1.672	1.672	3	300	30.0	15.0

Table S76. Masses (m) and turnover numbers (TON) of the products: Oxygenated products (Oxy), alkanes (Alk), *iso*-alkanes (Iso), and their sum (Tot).

	Oxy	Alk	Iso	Tot
m [mg]	0.000	0.002	0.000	0.002
TON [g/(kg d)]	0.000	0.000	0.000	0.000

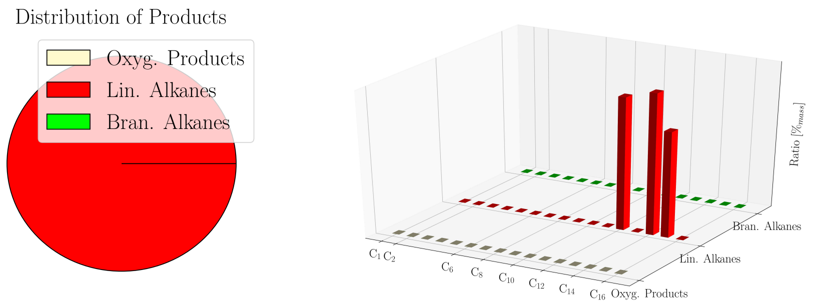

Figure S46. Distribution of products in the liquid phase.

Reaction 32: Campo del Cielo @ Montmorillonite

Table S77. Reaction conditions and masses of the entire catalysts (Cat) and its metal share (Met).

Synthesis	m(Cat) [g]	m(Met) [g]	t [d]	T [°C]	p(H_2) [bar]	p(CO_2) [bar]
Nano Particles	1.002	0.077	4	300	0.1	40.0

Table S78. Masses (m) and turnover numbers (TON) of the products: Oxygenated products (Oxy), alkanes (Alk), *iso*-alkanes (Iso), and their sum (Tot).

	Oxy	Alk	Iso	Tot
m [mg]	0.002	0.000	0.000	0.002
TON [g/(kg d)]	0.005	0.000	0.000	0.005

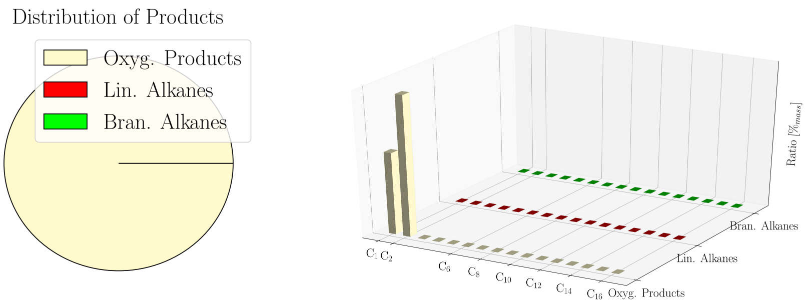

Figure S47. Distribution of products in the liquid phase.

Reaction 33: Campo del Cielo @ Montmorillonite

Table S79. Reaction conditions and masses of the entire catalysts (Cat) and its metal share (Met).

Synthesis	m(Cat) [g]	m(Met) [g]	t [d]	T [°C]	$p(H_2)$ [bar]	$p(CO_2)$ [bar]
Nano Particles	1.031	0.243	4	300	30.0	15.0

Table S80. Masses (m) and turnover numbers (TON) of the products: Oxygenated products (Oxy), alkanes (Alk), *iso*-alkanes (Iso), and their sum (Tot).

	Oxy	Alk	Iso	Tot
m [mg]	1.626	0.015	0.000	1.642
TON [g/(kg d)]	1.675	0.016	0.000	1.691

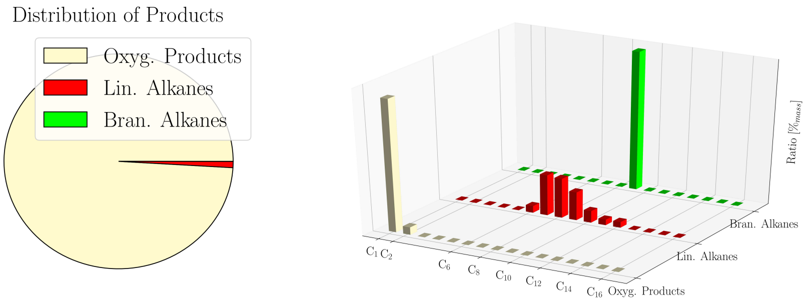

Figure S48. Distribution of products in the liquid phase.

Reaction 34: Volcanic Ash @ blank

Table S81. Reaction conditions and masses of the entire catalysts (Cat) and its metal share (Met).

Synthesis	m(Cat) [g]	m(Met) [g]	t [d]	T [°C]	$p(H_2)$ [bar]	$p(CO_2)$ [bar]
blank	1.065	0.128	3	300	30.0	15.0

Table S82. Masses (m) and turnover numbers (TON) of the products: Oxygenated products (Oxy), alkanes (Alk), *iso*-alkanes (Iso), and their sum (Tot).

	Oxy	Alk	Iso	Tot
m [mg]	0.002	0.000	0.000	0.002
TON [g/(kg d)]	0.005	0.000	0.000	0.005

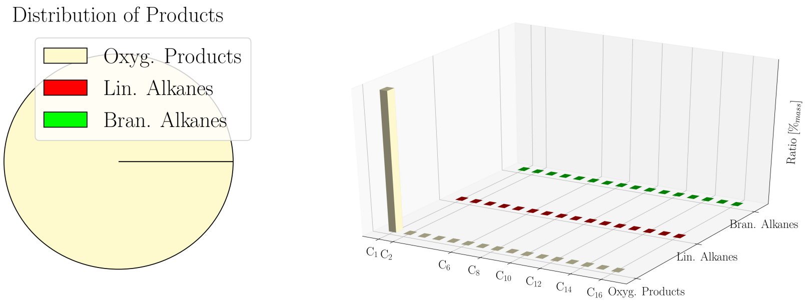

Figure S49. Distribution of products in the liquid phase.

Reaction 35: Campo del Cielo @ Montmorillonite

Table S83. Reaction conditions and masses of the entire catalysts (Cat) and its metal share (Met).

Synthesis	m(Cat) [g]	m(Met) [g]	t [d]	T [°C]	$p(H_2)$ [bar]	$p(CO_2)$ [bar]
Ball Mill	1.071	0.010	2	300	30.0	15.0

Table S84. Masses (m) and turnover numbers (TON) of the products: Oxygenated products (Oxy), alkanes (Alk), *iso*-alkanes (Iso), and their sum (Tot).

	Oxy	Alk	Iso	Tot
m [mg]	0.011	0.001	0.000	0.011
TON [g/(kg d)]	0.550	0.044	0.000	0.594

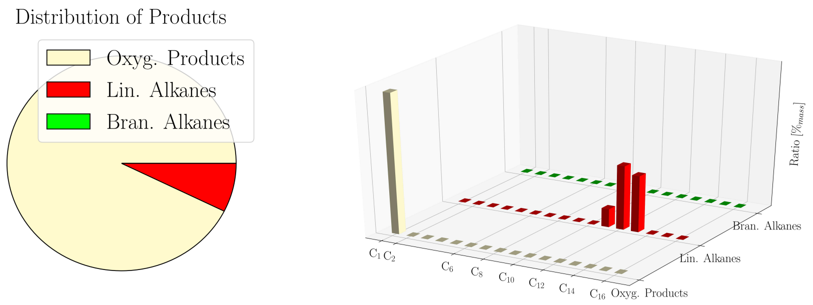

Figure S50. Distribution of products in the liquid phase.

Reaction 36: blank @ Diopside

Table S85. Reaction conditions and masses of the entire catalysts (Cat) and its metal share (Met).

Synthesis	m(Cat) [g]	m(Met) [g]	t [d]	T [°C]	p(H_2) [bar]	p(CO_2) [bar]
blank	0.998	0.000	3	300	30.0	15.0

Table S86. Masses (m) and turnover numbers (TON) of the products: Oxygenated products (Oxy), alkanes (Alk), *iso*-alkanes (Iso), and their sum (Tot).

	Oxy	Alk	Iso	Tot
m [mg]	0.000	0.000	0.000	0.000
TON [g/(kg d)]	0.000	0.000	0.000	0.000

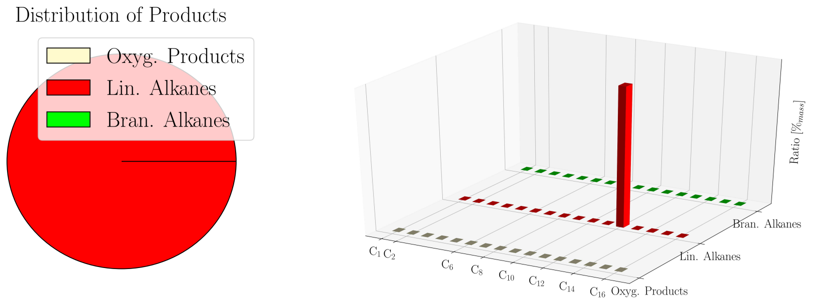

Figure S51. Distribution of products in the liquid phase.

Reaction 37: blank @ Olivine

Table S87. Reaction conditions and masses of the entire catalysts (Cat) and its metal share (Met).

Synthesis	m(Cat) [g]	m(Met) [g]	t [d]	T [°C]	p(H_2) [bar]	p(CO_2) [bar]
blank	1.011	0.000	3	300	30.0	15.0

Table S88. Masses (m) and turnover numbers (TON) of the products: Oxygenated products (Oxy), alkanes (Alk), *iso*-alkanes (Iso), and their sum (Tot).

	Oxy	Alk	Iso	Tot
m [mg]	0.000	0.000	0.000	0.000
TON [g/(kg d)]	0.000	0.000	0.000	0.000

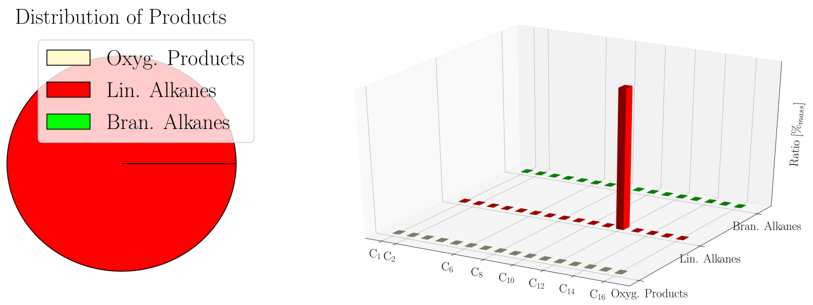

Figure S52. Distribution of products in the liquid phase.

Reaction 38: Muonionalusta @ Diopside

Table S89. Reaction conditions and masses of the entire catalysts (Cat) and its metal share (Met).

Synthesis	m(Cat) [g]	m(Met) [g]	t [d]	T [°C]	p(H_2) [bar]	p(CO_2) [bar]
Nano Particles	0.830	0.027	2	300	30.0	15.0

Table S90. Masses (m) and turnover numbers (TON) of the products: Oxygenated products (Oxy), alkanes (Alk), *iso*-alkanes (Iso), and their sum (Tot).

	Oxy	Alk	Iso	Tot
m [mg]	0.909	0.001	0.000	0.910
TON [g/(kg d)]	17.122	0.021	0.001	17.144

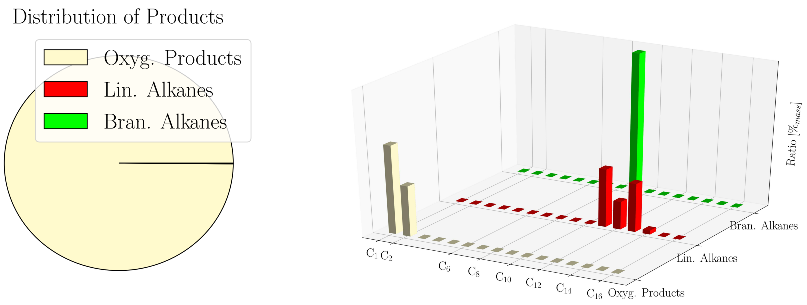

Figure S53. Distribution of products in the liquid phase.

Reaction 39: Campo del Cielo @ Hydroxyapatite

Table S91. Reaction conditions and masses of the entire catalysts (Cat) and its metal share (Met).

Synthesis	m(Cat) [g]	m(Met) [g]	t [d]	T [°C]	$p(H_2)$ [bar]	$p(CO_2)$ [bar]
Nano Particles	1.009	0.068	2	300	30.0	15.0

Table S92. Masses (m) and turnover numbers (TON) of the products: Oxygenated products (Oxy), alkanes (Alk), *iso*-alkanes (Iso), and their sum (Tot).

	Oxy	Alk	Iso	Tot
m [mg]	0.056	0.002	0.000	0.058
TON [g/(kg d)]	0.411	0.013	0.003	0.426

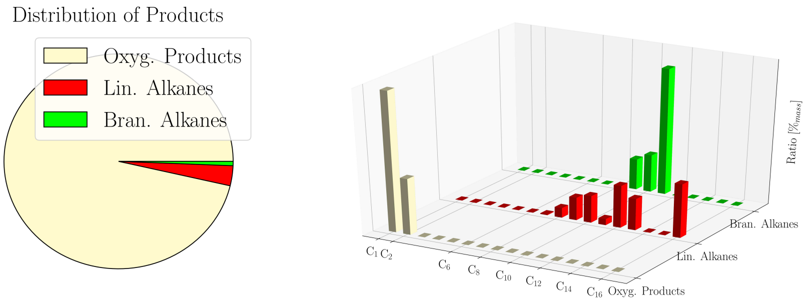

Figure S54. Distribution of products in the liquid phase.

Reaction 40: Gao-Guenie @ blank

Table S93. Reaction conditions and masses of the entire catalysts (Cat) and its metal share (Met).

Synthesis	m(Cat) [g]	m(Met) [g]	t [d]	T [°C]	p(H_2) [bar]	p(CO_2) [bar]
blank	0.818	0.164	2	300	30.0	15.0

Table S94. Masses (m) and turnover numbers (TON) of the products: Oxygenated products (Oxy), alkanes (Alk), *iso*-alkanes (Iso), and their sum (Tot).

	Oxy	Alk	Iso	Tot
m [mg]	0.023	0.001	-0.000	0.024
TON [g/(kg d)]	0.070	0.002	0.000	0.072

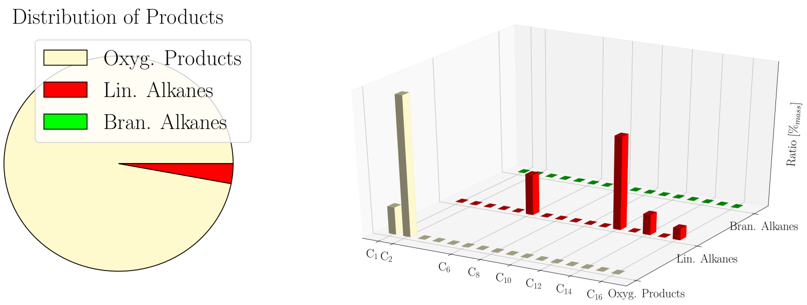

Figure S55. Distribution of products in the liquid phase.

Reaction 41: Muonionalusta @ Hydroxyapatite

Table S95. Reaction conditions and masses of the entire catalysts (Cat) and its metal share (Met).

Synthesis	m(Cat) [g]	m(Met) [g]	t [d]	T [°C]	p(H_2) [bar]	p(CO_2) [bar]
Nano Particles	1.022	0.033	3	300	30.0	15.0

Table S96. Masses (m) and turnover numbers (TON) of the products: Oxygenated products (Oxy), alkanes (Alk), *iso*-alkanes (Iso), and their sum (Tot).

	Oxy	Alk	Iso	Tot
m [mg]	0.000	0.000	0.000	0.000
TON [g/(kg d)]	0.000	0.001	0.000	0.001

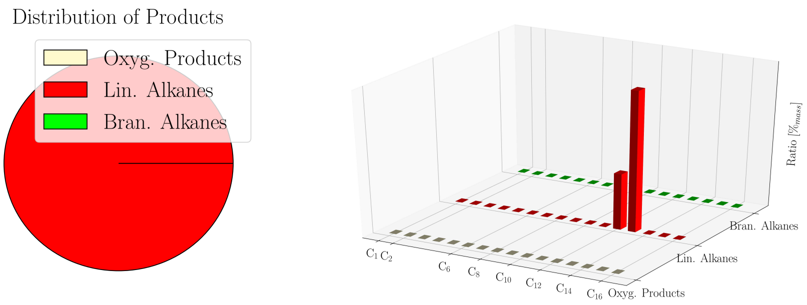

Figure S56. Distribution of products in the liquid phase.

Reaction 42: Muonionalusta @ Olivine

Table S97. Reaction conditions and masses of the entire catalysts (Cat) and its metal share (Met).

Synthesis	m(Cat) [g]	m(Met) [g]	t [d]	T [°C]	p(H_2) [bar]	p(CO_2) [bar]
Nano Particles	0.992	0.032	2	300	30.0	15.0

Table S98. Masses (m) and turnover numbers (TON) of the products: Oxygenated products (Oxy), alkanes (Alk), *iso*-alkanes (Iso), and their sum (Tot).

	Oxy	Alk	Iso	Tot
m [mg]	0.001	0.001	0.000	0.002
TON [g/(kg d)]	0.012	0.018	0.007	0.036

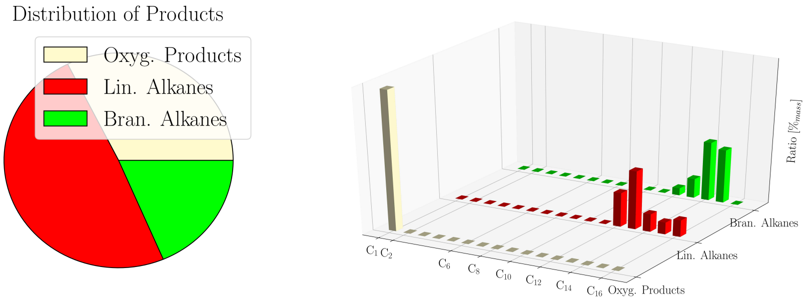

Figure S57. Distribution of products in the liquid phase.

Reaction 43: Volcanic Ash @ Diopside

Table S99. Reaction conditions and masses of the entire catalysts (Cat) and its metal share (Met).

Synthesis	m(Cat) [g]	m(Met) [g]	t [d]	T [°C]	p(H_2) [bar]	p(CO_2) [bar]
Ball Mill	0.771	0.003	2	300	30.0	15.0

Table S100. Masses (m) and turnover numbers (TON) of the products: Oxygenated products (Oxy), alkanes (Alk), *iso*-alkanes (Iso), and their sum (Tot).

	Oxy	Alk	Iso	Tot
m [mg]	0.041	0.003	0.002	0.046
TON [g/(kg d)]	7.701	0.565	0.284	8.550

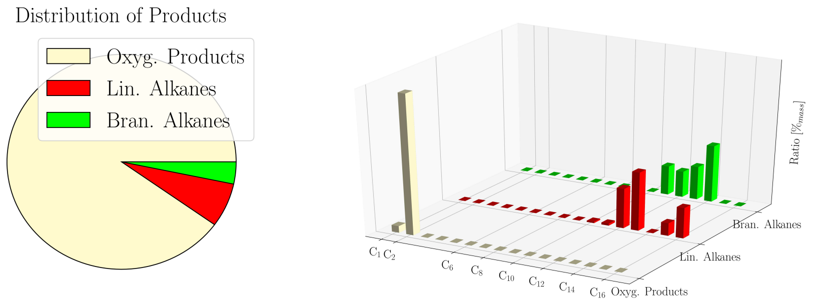

Figure S58. Distribution of products in the liquid phase.

Reaction 44: Volcanic Ash @ Olivine

Table S101. Reaction conditions and masses of the entire catalysts (Cat) and its metal share (Met).

Synthesis	m(Cat) [g]	m(Met) [g]	t [d]	T [°C]	p(H_2) [bar]	p(CO_2) [bar]
Ball Mill	1.055	0.005	2	300	30.0	15.0

Table S102. Masses (m) and turnover numbers (TON) of the products: Oxygenated products (Oxy), alkanes (Alk), *iso*-alkanes (Iso), and their sum (Tot).

	Oxy	Alk	Iso	Tot
m [mg]	0.001	0.000	0.000	0.001
TON [g/(kg d)]	0.148	0.000	0.000	0.148

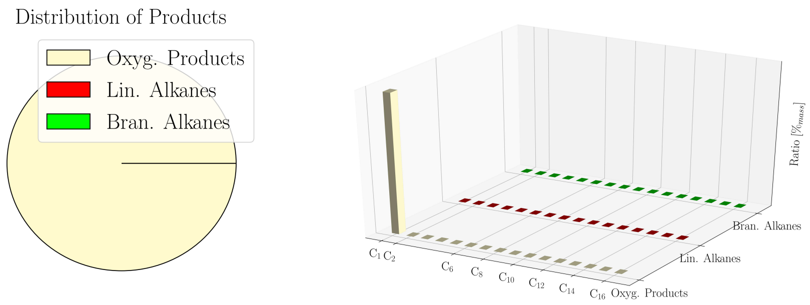

Figure S59. Distribution of products in the liquid phase.

Reaction 45: Volcanic Ash @ Hydroxyapatite

Table S103. Reaction conditions and masses of the entire catalysts (Cat) and its metal share (Met).

Synthesis	m(Cat) [g]	m(Met) [g]	t [d]	T [°C]	p(H_2) [bar]	p(CO_2) [bar]
Ball Mill	0.999	0.009	3	300	30.0	15.0

Table S104. Masses (m) and turnover numbers (TON) of the products: Oxygenated products (Oxy), alkanes (Alk), *iso*-alkanes (Iso), and their sum (Tot).

	Oxy	Alk	Iso	Tot
m [mg]	0.007	0.000	0.000	0.008
TON [g/(kg d)]	0.283	0.009	0.013	0.305

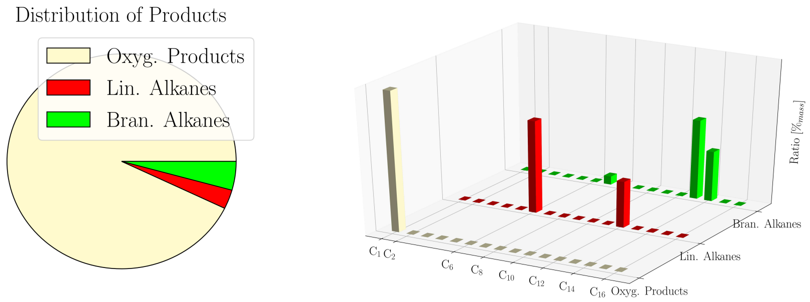

Figure S60. Distribution of products in the liquid phase.

Reaction 46: Volcanic Ash @ Montmorillonite

Table S105. Reaction conditions and masses of the entire catalysts (Cat) and its metal share (Met).

Synthesis	m(Cat) [g]	m(Met) [g]	t [d]	T [°C]	p(H_2) [bar]	p(CO_2) [bar]
Ball Mill	1.012	0.004	3	300	30.0	15.0

Table S106. Masses (m) and turnover numbers (TON) of the products: Oxygenated products (Oxy), alkanes (Alk), *iso*-alkanes (Iso), and their sum (Tot).

	Oxy	Alk	Iso	Tot
m [mg]	0.000	0.001	0.001	0.002
TON [g/(kg d)]	0.000	0.077	0.075	0.152

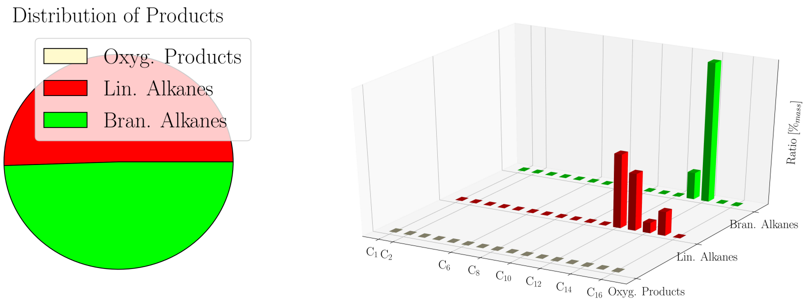

Figure S61. Distribution of products in the liquid phase.

Reaction 47: Volcanic Ash @ Silica Gel

Table S107. Reaction conditions and masses of the entire catalysts (Cat) and its metal share (Met).

Synthesis	m(Cat) [g]	m(Met) [g]	t [d]	T [°C]	p(H_2) [bar]	p(CO_2) [bar]
Ball Mill	1.055	0.003	2	300	30.0	15.0

Table S108. Masses (m) and turnover numbers (TON) of the products: Oxygenated products (Oxy), alkanes (Alk), *iso*-alkanes (Iso), and their sum (Tot).

	Oxy	Alk	Iso	Tot
m [mg]	0.008	0.000	-0.000	0.008
TON [g/(kg d)]	1.474	0.007	0.000	1.481

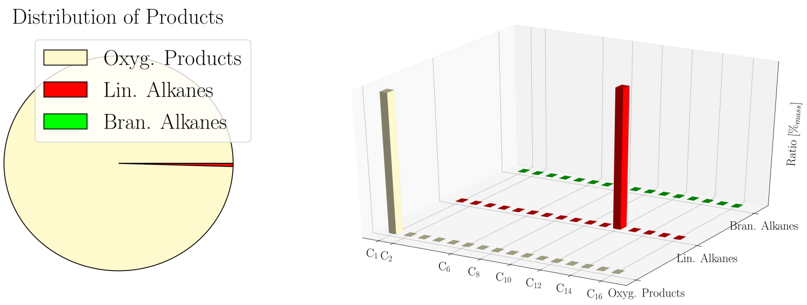

Figure S62. Distribution of products in the liquid phase.

Reaction 48: Campo del Cielo @ Hydroxyapatite

Table S109. Reaction conditions and masses of the entire catalysts (Cat) and its metal share (Met).

Synthesis	m(Cat) [g]	m(Met) [g]	t [d]	T [°C]	$p(H_2)$ [bar]	$p(CO_2)$ [bar]
Ball Mill	1.010	0.023	2	300	30.0	15.0

Table S110. Masses (m) and turnover numbers (TON) of the products: Oxygenated products (Oxy), alkanes (Alk), *iso*-alkanes (Iso), and their sum (Tot).

	Oxy	Alk	Iso	Tot
m [mg]	0.003	0.000	0.000	0.003
TON [g/(kg d)]	0.058	0.005	0.004	0.068

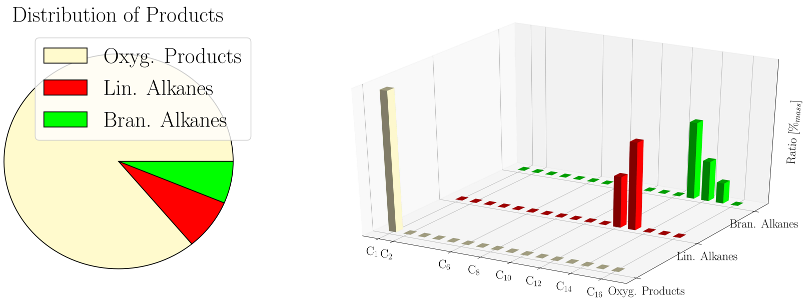

Figure S63. Distribution of products in the liquid phase.

Reaction 49: Campo del Cielo @ Olivine

Table S111. Reaction conditions and masses of the entire catalysts (Cat) and its metal share (Met).

Synthesis	m(Cat) [g]	m(Met) [g]	t [d]	T [°C]	p(H_2) [bar]	p(CO_2) [bar]
Ball Mill	1.043	0.004	3	300	30.0	15.0

Table S112. Masses (m) and turnover numbers (TON) of the products: Oxygenated products (Oxy), alkanes (Alk), *iso*-alkanes (Iso), and their sum (Tot).

	Oxy	Alk	Iso	Tot
m [mg]	0.000	0.000	0.000	0.000
TON [g/(kg d)]	0.000	0.000	0.000	0.000

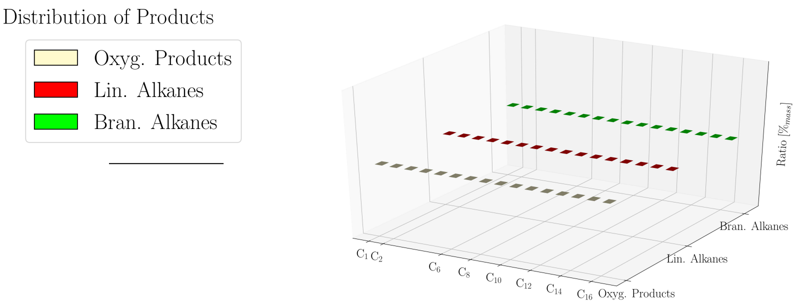

Figure S64. Distribution of products in the liquid phase.

Reaction 50: Gao-Guenie @ Diopside

Table S113. Reaction conditions and masses of the entire catalysts (Cat) and its metal share (Met).

Synthesis	m(Cat) [g]	m(Met) [g]	t [d]	T [°C]	p(H_2) [bar]	p(CO_2) [bar]
Nano Particles	1.228	0.016	2	300	30.0	15.0

Table S114. Masses (m) and turnover numbers (TON) of the products: Oxygenated products (Oxy), alkanes (Alk), *iso*-alkanes (Iso), and their sum (Tot).

	Oxy	Alk	Iso	Tot
m [mg]	0.000	0.003	0.000	0.003
TON [g/(kg d)]	0.000	0.078	0.000	0.078

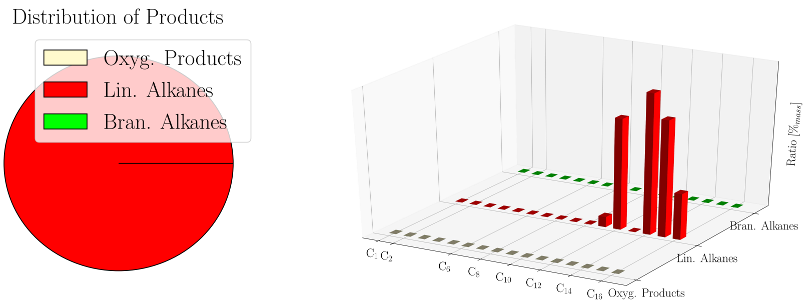

Figure S65. Distribution of products in the liquid phase.

Reaction 51: Gao-Guenie @ Silica Gel

Table S115. Reaction conditions and masses of the entire catalysts (Cat) and its metal share (Met).

Synthesis	m(Cat) [g]	m(Met) [g]	t [d]	T [°C]	p(H_2) [bar]	p(CO_2) [bar]
Nano Particles	1.243	0.016	2	300	30.0	15.0

Table S116. Masses (m) and turnover numbers (TON) of the products: Oxygenated products (Oxy), alkanes (Alk), *iso*-alkanes (Iso), and their sum (Tot).

	Oxy	Alk	Iso	Tot
m [mg]	0.000	0.000	0.000	0.000
TON [g/(kg d)]	0.000	0.013	0.000	0.013

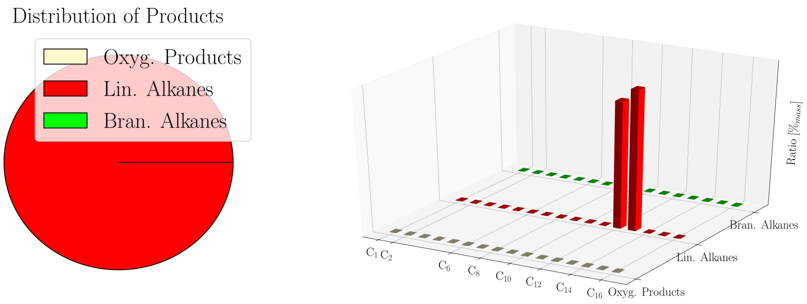

Figure S66. Distribution of products in the liquid phase.

Reaction 52: Gao-Guenie @ Montmorillonite

Table S117. Reaction conditions and masses of the entire catalysts (Cat) and its metal share (Met).

Synthesis	m(Cat) [g]	m(Met) [g]	t [d]	T [°C]	$p(H_2)$ [bar]	$p(CO_2)$ [bar]
Nano Particles	1.056	0.015	2	300	30.0	15.0

Table S118. Masses (m) and turnover numbers (TON) of the products: Oxygenated products (Oxy), alkanes (Alk), *iso*-alkanes (Iso), and their sum (Tot).

	Oxy	Alk	Iso	Tot
m [mg]	0.017	0.000	0.000	0.018
TON [g/(kg d)]	0.597	0.005	0.002	0.603

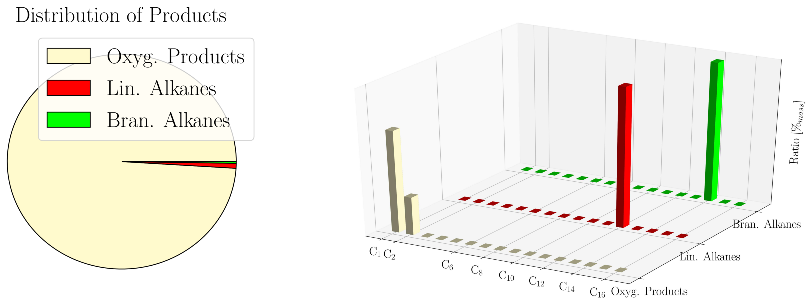

Figure S67. Distribution of products in the liquid phase.

Reaction 53: Volcanic Ash @ Silica Gel

Table S119. Reaction conditions and masses of the entire catalysts (Cat) and its metal share (Met).

Synthesis	m(Cat) [g]	m(Met) [g]	t [d]	T [°C]	$p(H_2)$ [bar]	$p(CO_2)$ [bar]
Nano Particles	1.243	0.014	2	300	30.0	15.0

Table S120. Masses (m) and turnover numbers (TON) of the products: Oxygenated products (Oxy), alkanes (Alk), *iso*-alkanes (Iso), and their sum (Tot).

	Oxy	Alk	Iso	Tot
m [mg]	1.066	0.006	0.006	1.077
TON [g/(kg d)]	37.989	0.223	0.202	38.413

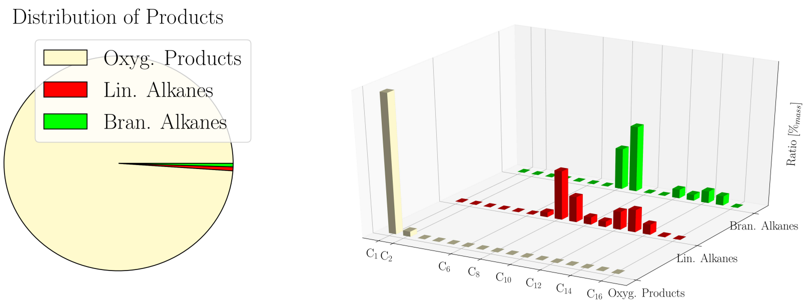

Figure S68. Distribution of products in the liquid phase.

Reaction 54: Volcanic Ash @ Montmorillonite

Table S121. Reaction conditions and masses of the entire catalysts (Cat) and its metal share (Met).

Synthesis	m(Cat) [g]	m(Met) [g]	t [d]	T [°C]	$p(H_2)$ [bar]	$p(CO_2)$ [bar]
Nano Particles	1.144	0.008	2	300	30.0	15.0

Table S122. Masses (m) and turnover numbers (TON) of the products: Oxygenated products (Oxy), alkanes (Alk), *iso*-alkanes (Iso), and their sum (Tot).

	Oxy	Alk	Iso	Tot
m [mg]	0.013	0.001	0.000	0.014
TON [g/(kg d)]	0.811	0.037	0.020	0.867

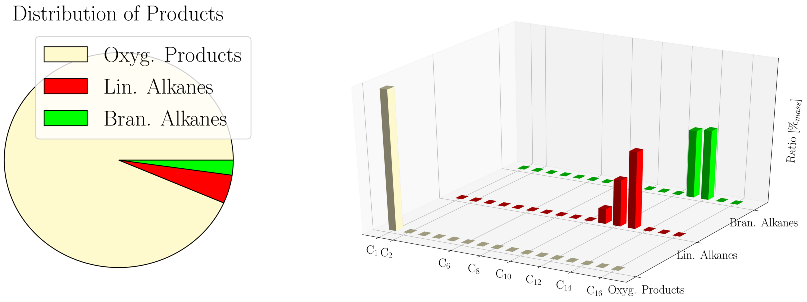

Figure S69. Distribution of products in the liquid phase.

Reaction 55: Volcanic Ash @ Hydroxyapatite

Table S123. Reaction conditions and masses of the entire catalysts (Cat) and its metal share (Met).

Synthesis	m(Cat) [g]	m(Met) [g]	t [d]	T [°C]	p(H_2) [bar]	p(CO_2) [bar]
Nano Particles	0.857	0.006	2	300	30.0	15.0

Table S124. Masses (m) and turnover numbers (TON) of the products: Oxygenated products (Oxy), alkanes (Alk), *iso*-alkanes (Iso), and their sum (Tot).

	Oxy	Alk	Iso	Tot
m [mg]	0.000	0.001	0.001	0.001
TON [g/(kg d)]	0.000	0.066	0.053	0.119

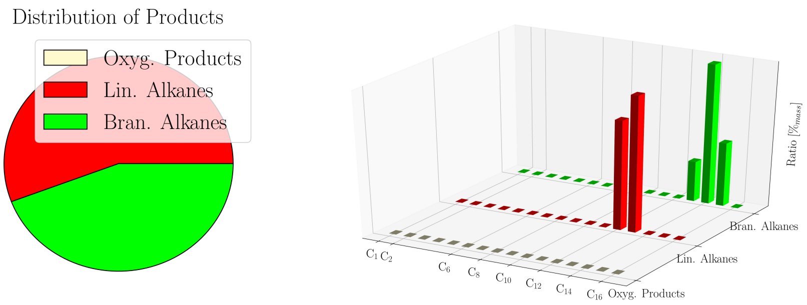

Figure S70. Distribution of products in the liquid phase.

Reaction 56: Volcanic Ash @ Diopside

Table S125. Reaction conditions and masses of the entire catalysts (Cat) and its metal share (Met).

Synthesis	m(Cat) [g]	m(Met) [g]	t [d]	T [°C]	p(H_2) [bar]	p(CO_2) [bar]
Nano Particles	1.008	0.009	2	300	30.0	15.0

Table S126. Masses (m) and turnover numbers (TON) of the products: Oxygenated products (Oxy), alkanes (Alk), *iso*-alkanes (Iso), and their sum (Tot).

	Oxy	Alk	Iso	Tot
m [mg]	1.588	0.023	0.002	1.613
TON [g/(kg d)]	84.198	1.217	0.083	85.498

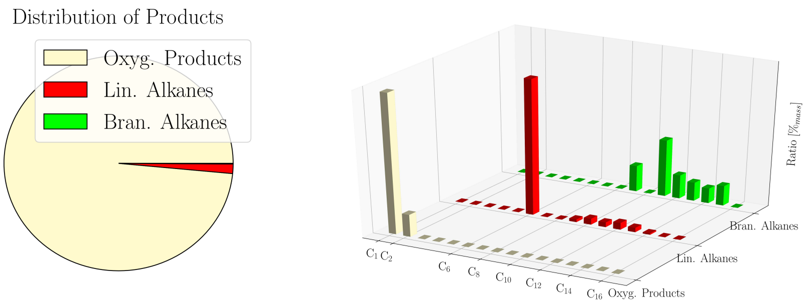

Figure S71. Distribution of products in the liquid phase.

Reaction 57: Volcanic Ash @ Olivine

Table S127. Reaction conditions and masses of the entire catalysts (Cat) and its metal share (Met).

Synthesis	m(Cat) [g]	m(Met) [g]	t [d]	T [°C]	p(H_2) [bar]	p(CO_2) [bar]
Nano Particles	1.012	0.009	2	300	30.0	15.0

Table S128. Masses (m) and turnover numbers (TON) of the products: Oxygenated products (Oxy), alkanes (Alk), *iso*-alkanes (Iso), and their sum (Tot).

	Oxy	Alk	Iso	Tot
m [mg]	0.096	0.001	0.000	0.097
TON [g/(kg d)]	5.072	0.030	0.025	5.127

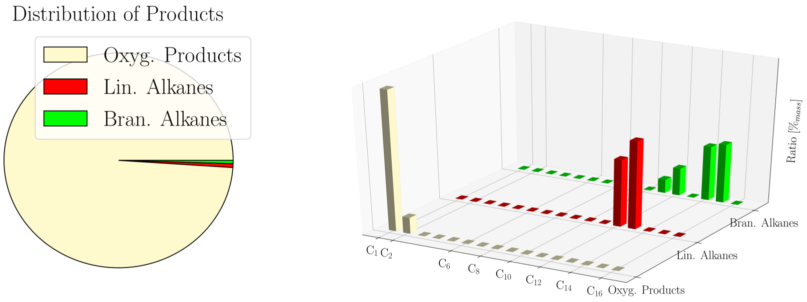

Figure S72. Distribution of products in the liquid phase.

Reaction 58: Gao-Guenie @ Olivine

Table S129. Reaction conditions and masses of the entire catalysts (Cat) and its metal share (Met).

Synthesis	m(Cat) [g]	m(Met) [g]	t [d]	T [°C]	p(H_2) [bar]	p(CO_2) [bar]
Nano Particles	1.151	0.020	2	300	30.0	15.0

Table S130. Masses (m) and turnover numbers (TON) of the products: Oxygenated products (Oxy), alkanes (Alk), *iso*-alkanes (Iso), and their sum (Tot).

	Oxy	Alk	Iso	Tot
m [mg]	0.000	0.000	0.000	0.001
TON [g/(kg d)]	0.005	0.010	-0.000	0.016

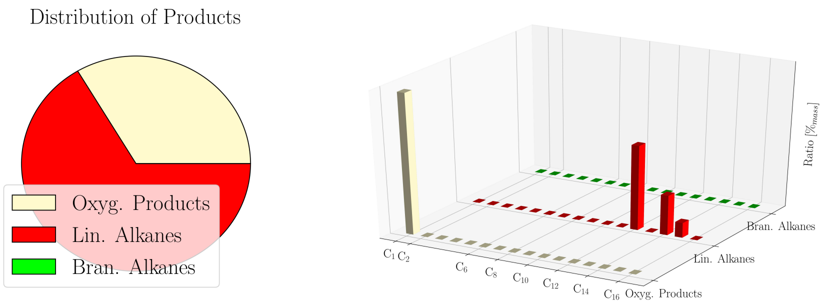

Figure S73. Distribution of products in the liquid phase.

Reaction 59: blank @ Hydroxyapatite

Table S131. Reaction conditions and masses of the entire catalysts (Cat) and its metal share (Met).

Synthesis	m(Cat) [g]	m(Met) [g]	t [d]	T [°C]	p(H_2) [bar]	p(CO_2) [bar]
blank	1.151	0.000	2	300	30.0	15.0

Table S132. Masses (m) and turnover numbers (TON) of the products: Oxygenated products (Oxy), alkanes (Alk), *iso*-alkanes (Iso), and their sum (Tot).

	Oxy	Alk	Iso	Tot
m [mg]	0.000	0.002	0.000	0.002
TON [g/(kg d)]	0.000	0.001	0.000	0.001

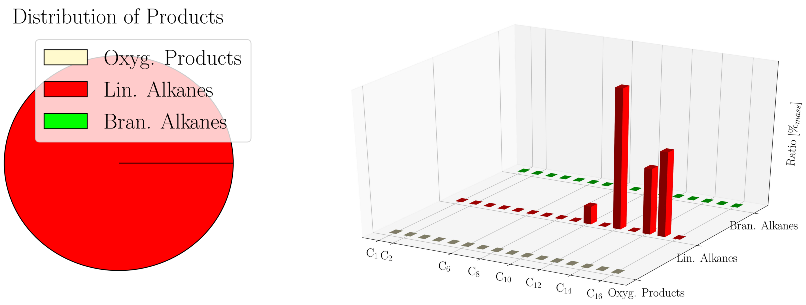

Figure S74. Distribution of products in the liquid phase.

Reaction 60: Gao-Guenie @ Hydroxyapatite

Table S133. Reaction conditions and masses of the entire catalysts (Cat) and its metal share (Met).

Synthesis	m(Cat) [g]	m(Met) [g]	t [d]	T [°C]	p(H_2) [bar]	p(CO_2) [bar]
Nano Particles	0.978	0.013	4	300	30.0	15.0

Table S134. Masses (m) and turnover numbers (TON) of the products: Oxygenated products (Oxy), alkanes (Alk), *iso*-alkanes (Iso), and their sum (Tot).

	Oxy	Alk	Iso	Tot
m [mg]	0.001	0.000	0.000	0.001
TON [g/(kg d)]	0.027	0.001	0.000	0.027

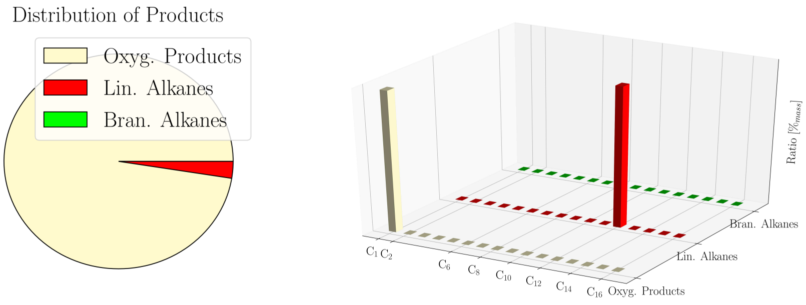

Figure S75. Distribution of products in the liquid phase.

Reaction 61: Campo del Cielo @ Silica Gel

Table S135. Reaction conditions and masses of the entire catalysts (Cat) and its metal share (Met).

Synthesis	m(Cat) [g]	m(Met) [g]	t [d]	T [°C]	p(H_2) [bar]	p(CO_2) [bar]
Ball Mill	1.003	0.009	2	300	30.0	15.0

Table S136. Masses (m) and turnover numbers (TON) of the products: Oxygenated products (Oxy), alkanes (Alk), *iso*-alkanes (Iso), and their sum (Tot).

	Oxy	Alk	Iso	Tot
m [mg]	0.159	0.002	0.001	0.162
TON [g/(kg d)]	8.807	0.108	0.077	8.992

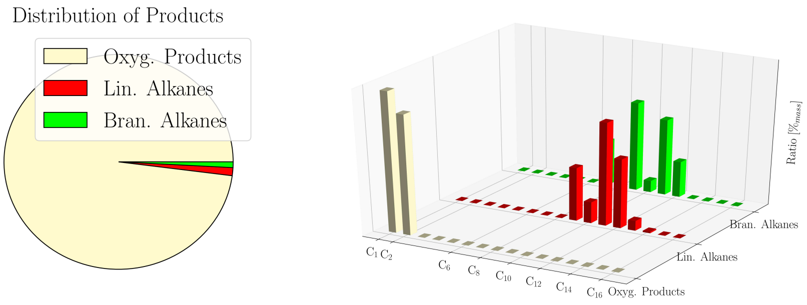

Figure S76. Distribution of products in the liquid phase.

Reaction 62: Muonionalusta @ Silica Gel

Table S137. Reaction conditions and masses of the entire catalysts (Cat) and its metal share (Met).

Synthesis	m(Cat) [g]	m(Met) [g]	t [d]	T [°C]	p(H_2) [bar]	p(CO_2) [bar]
Nano Particles	1.002	0.086	2	300	15.0	30.0

Table S138. Masses (m) and turnover numbers (TON) of the products: Oxygenated products (Oxy), alkanes (Alk), *iso*-alkanes (Iso), and their sum (Tot).

	Oxy	Alk	Iso	Tot
m [mg]	2.852	0.077	0.000	2.930
TON [g/(kg d)]	16.609	0.451	0.000	17.060

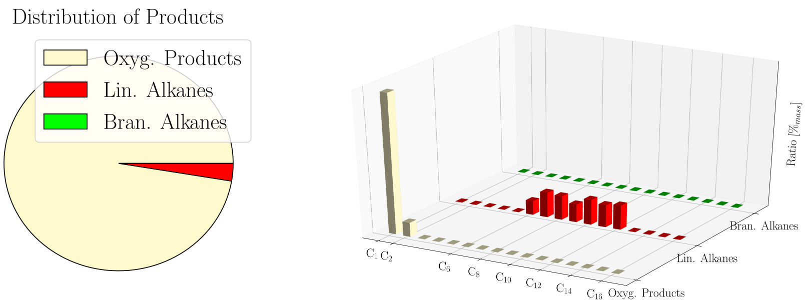

Figure S77. Distribution of products in the liquid phase.

Reaction 63: Campo del Cielo @ Montmorillonite

Table S139. Reaction conditions and masses of the entire catalysts (Cat) and its metal share (Met).

Synthesis	m(Cat) [g]	m(Met) [g]	t [d]	T [°C]	p(H_2) [bar]	p(CO_2) [bar]
Nano Particles	1.042	0.080	3	300	30.0	15.0

Table S140. Masses (m) and turnover numbers (TON) of the products: Oxygenated products (Oxy), alkanes (Alk), *iso*-alkanes (Iso), and their sum (Tot).

	Oxy	Alk	Iso	Tot
m [mg]	0.682	0.152	0.120	0.953
TON [g/(kg d)]	2.837	0.632	0.499	3.968

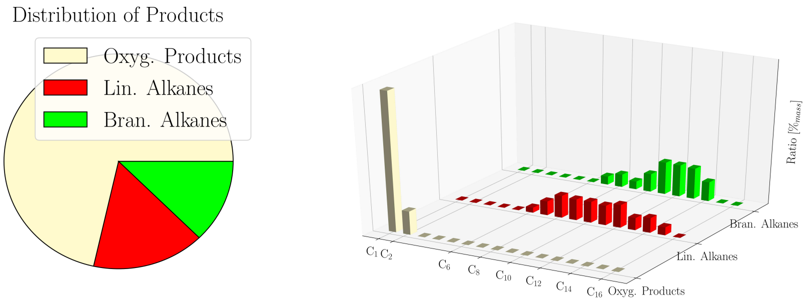

Figure S78. Distribution of products in the liquid phase.

Reaction 64: blank Dichlormethane

Table S141. Reaction conditions and masses of the entire catalysts (Cat) and its metal share (Met).

Synthesis	m(Cat) [g]	m(Met) [g]	t [d]	T [°C]	p(H_2) [bar]	p(CO_2) [bar]
Nano Particles	0.000	0.000	0	0	0.0	0.0

Table S142. Masses (m) and turnover numbers (TON) of the products: Oxygenated products (Oxy), alkanes (Alk), *iso*-alkanes (Iso), and their sum (Tot).

	Oxy	Alk	Iso	Tot
m [mg]	0.001	0.000	0.000	0.001
TON [g/(kg d)]	inf	0.000	nan	inf

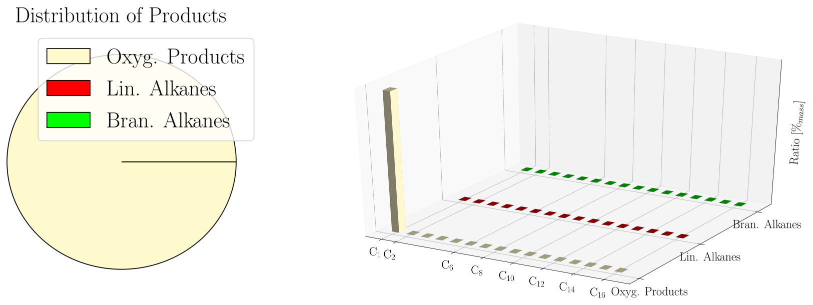

Figure S79. Distribution of products in the liquid phase.

Reaction 65: blank @ blank

Table S143. Reaction conditions and masses of the entire catalysts (Cat) and its metal share (Met).

Synthesis	m(Cat) [g]	m(Met) [g]	t [d]	T [°C]	p(H_2) [bar]	p(CO_2) [bar]
blank	1.000	1.000	5	300	30.0	15.0

Table S144. Masses (m) and turnover numbers (TON) of the products: Oxygenated products (Oxy), alkanes (Alk), *iso*-alkanes (Iso), and their sum (Tot).

	Oxy	Alk	Iso	Tot
m [mg]	0.000	0.000	0.000	0.000
TON [g/(kg d)]	0.000	0.000	0.000	0.000

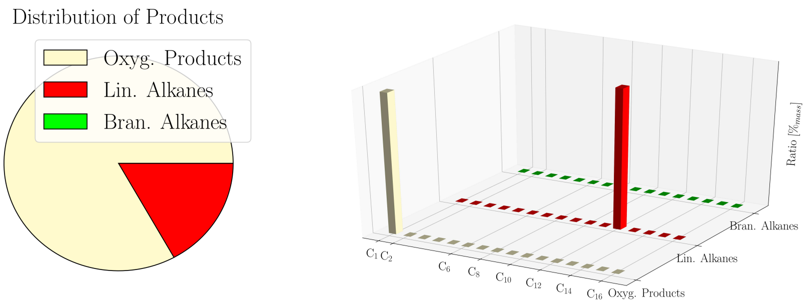

Figure S80. Distribution of products in the liquid phase.

Reaction 67: Campo del Cielo @ blank

Table S145. Reaction conditions and masses of the entire catalysts (Cat) and its metal share (Met).

Synthesis	m(Cat) [g]	m(Met) [g]	t [d]	T [°C]	p(H_2) [bar]	p(CO_2) [bar]
Ball Mill	0.598	0.598	3	300	30.0	15.0

Table S146. Masses (m) and turnover numbers (TON) of the products: Oxygenated products (Oxy), alkanes (Alk), *iso*-alkanes (Iso), and their sum (Tot).

	Oxy	Alk	Iso	Tot
m [mg]	0.000	0.000	0.000	0.000
TON [g/(kg d)]	0.000	0.000	0.000	0.000

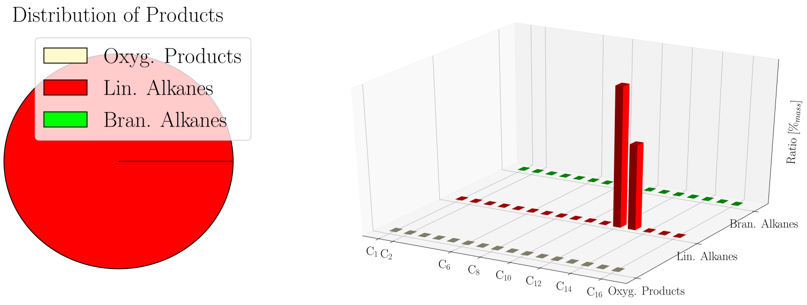

Figure S81. Distribution of products in the liquid phase.

Reaction 68: Campo del Cielo @ blank

Table S147. Reaction conditions and masses of the entire catalysts (Cat) and its metal share (Met).

Synthesis	m(Cat) [g]	m(Met) [g]	t [d]	T [°C]	p(H_2) [bar]	p(CO_2) [bar]
Nano Particles	1.000	1.000	4	300	30.0	15.0

Table S148. Masses (m) and turnover numbers (TON) of the products: Oxygenated products (Oxy), alkanes (Alk), *iso*-alkanes (Iso), and their sum (Tot).

	Oxy	Alk	Iso	Tot
m [mg]	0.015	0.001	0.000	0.016
TON [g/(kg d)]	0.004	0.000	-0.000	0.004

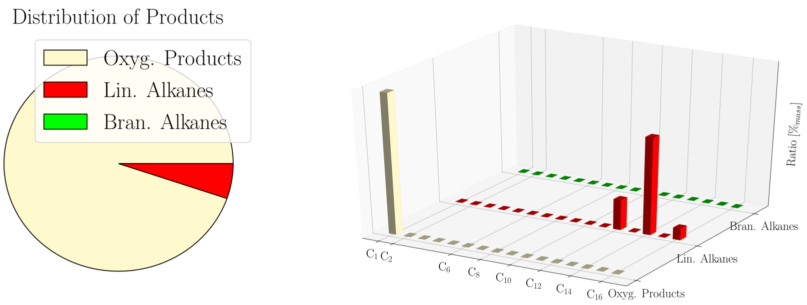

Figure S82. Distribution of products in the liquid phase.

Reaction 69: Campo del Cielo @ Diopside

Table S149. Reaction conditions and masses of the entire catalysts (Cat) and its metal share (Met).

Synthesis	m(Cat) [g]	m(Met) [g]	t [d]	T [°C]	p(H_2) [bar]	p(CO_2) [bar]
Nano Particles	1.009	0.075	4	300	30.0	15.0

Table S150. Masses (m) and turnover numbers (TON) of the products: Oxygenated products (Oxy), alkanes (Alk), *iso*-alkanes (Iso), and their sum (Tot).

	Oxy	Alk	Iso	Tot
m [mg]	2.130	0.074	0.004	2.208
TON [g/(kg d)]	7.128	0.248	0.013	7.389

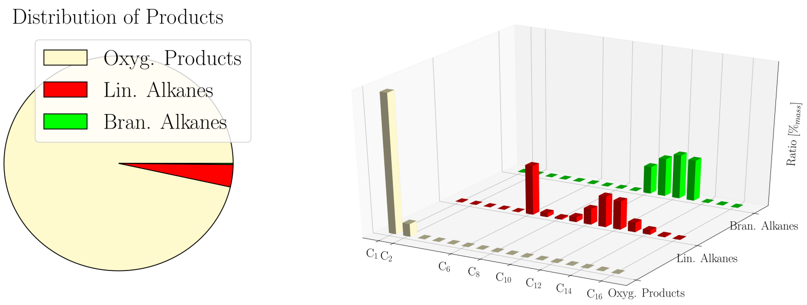

Figure S83. Distribution of products in the liquid phase.

Reaction 70: Campo del Cielo @ Diopside

Table S151. Reaction conditions and masses of the entire catalysts (Cat) and its metal share (Met).

Synthesis	m(Cat) [g]	m(Met) [g]	t [d]	T [°C]	$p(H_2)$ [bar]	$p(CO_2)$ [bar]
Ball Mill	1.020	0.003	2	300	30.0	15.0

Table S152. Masses (m) and turnover numbers (TON) of the products: Oxygenated products (Oxy), alkanes (Alk), *iso*-alkanes (Iso), and their sum (Tot).

	Oxy	Alk	Iso	Tot
m [mg]	0.051	0.000	0.000	0.051
TON [g/(kg d)]	8.255	0.040	0.000	8.295

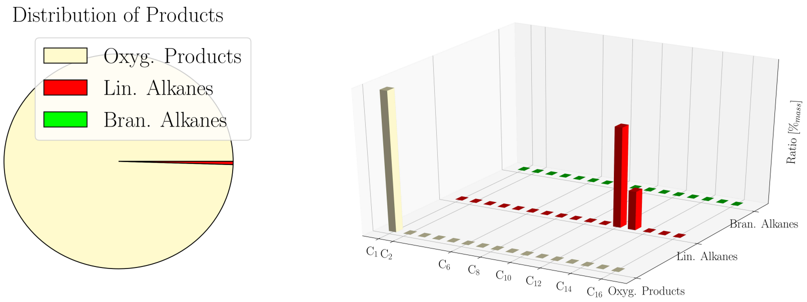

Figure S84. Distribution of products in the liquid phase.

Reaction 71: Campo del Cielo @ synthetic Olivine

Table S153. Reaction conditions and masses of the entire catalysts (Cat) and its metal share (Met).

Synthesis	m(Cat) [g]	m(Met) [g]	t [d]	T [°C]	p(H_2) [bar]	p(CO_2) [bar]
Nano Particles	0.541	0.064	3	300	30.0	15.0

Table S154. Masses (m) and turnover numbers (TON) of the products: Oxygenated products (Oxy), alkanes (Alk), *iso*-alkanes (Iso), and their sum (Tot).

	Oxy	Alk	Iso	Tot
m [mg]	0.640	0.005	0.002	0.646
TON [g/(kg d)]	3.350	0.026	0.008	3.384

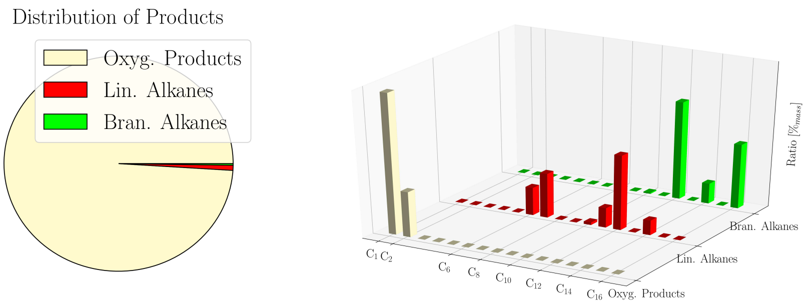

Figure S85. Distribution of products in the liquid phase.

Reaction 72: Campo del Cielo @ Calcium Carbonate

Table S155. Reaction conditions and masses of the entire catalysts (Cat) and its metal share (Met).

Synthesis	m(Cat) [g]	m(Met) [g]	t [d]	T [°C]	p(H_2) [bar]	p(CO_2) [bar]
Nano Particles	1.000	0.053	4	300	30.0	15.0

Table S156. Masses (m) and turnover numbers (TON) of the products: Oxygenated products (Oxy), alkanes (Alk), *iso*-alkanes (Iso), and their sum (Tot).

	Oxy	Alk	Iso	Tot
m [mg]	0.059	0.000	0.000	0.059
TON [g/(kg d)]	0.275	0.000	0.000	0.275

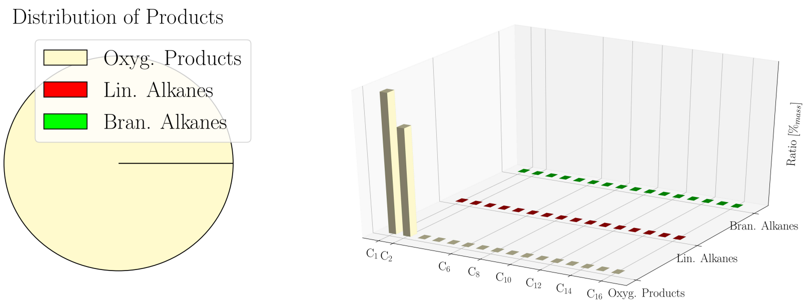

Figure S86. Distribution of products in the liquid phase.

Reaction 73: Campo del Cielo @ Hydroxyapatite

Table S157. Reaction conditions and masses of the entire catalysts (Cat) and its metal share (Met).

Synthesis	m(Cat) [g]	m(Met) [g]	t [d]	T [°C]	p(H_2) [bar]	p(CO_2) [bar]
Nano Particles	1.051	0.038	4	300	30.0	15.0

Table S158. Masses (m) and turnover numbers (TON) of the products: Oxygenated products (Oxy), alkanes (Alk), *iso*-alkanes (Iso), and their sum (Tot).

	Oxy	Alk	Iso	Tot
m [mg]	0.151	0.000	0.000	0.151
TON [g/(kg d)]	0.997	0.000	0.000	0.997

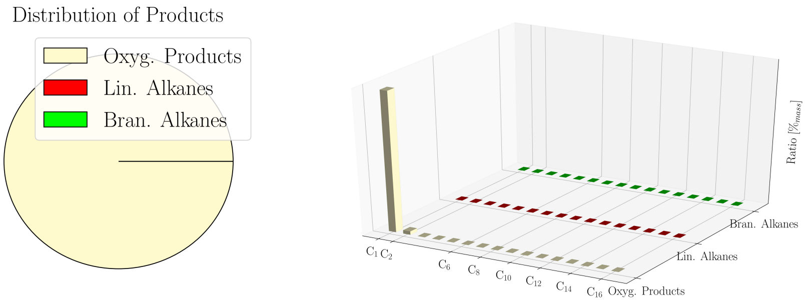

Figure S87. Distribution of products in the liquid phase.

Reaction 74: Campo del Cielo @ Silica Gel

Table S159. Reaction conditions and masses of the entire catalysts (Cat) and its metal share (Met).

Synthesis	m(Cat) [g]	m(Met) [g]	t [d]	T [°C]	p(H_2) [bar]	p(CO_2) [bar]
Ball Mill	0.992	0.005	10	300	30.0	15.0

Table S160. Masses (m) and turnover numbers (TON) of the products: Oxygenated products (Oxy), alkanes (Alk), *iso*-alkanes (Iso), and their sum (Tot).

	Oxy	Alk	Iso	Tot
m [mg]	0.008	0.000	-0.000	0.008
TON [g/(kg d)]	0.168	0.001	0.000	0.169

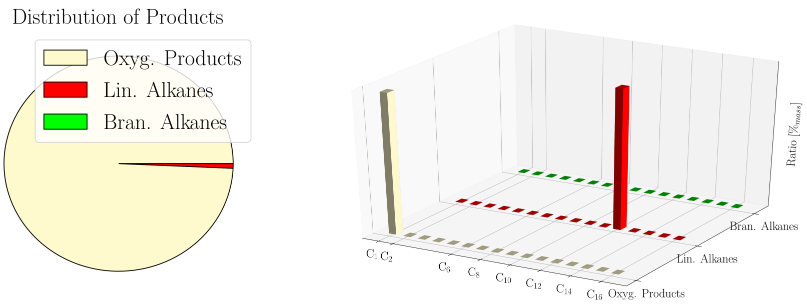

Figure S88. Distribution of products in the liquid phase.

Reaction 75: Campo del Cielo @ Montmorillonite

Table S161. Reaction conditions and masses of the entire catalysts (Cat) and its metal share (Met).

Synthesis	m(Cat) [g]	m(Met) [g]	t [d]	T [°C]	p(H_2) [bar]	p(CO_2) [bar]
Nano Particles	1.078	0.083	10	200	1.0	20.0

Table S162. Masses (m) and turnover numbers (TON) of the products: Oxygenated products (Oxy), alkanes (Alk), *iso*-alkanes (Iso), and their sum (Tot).

	Oxy	Alk	Iso	Tot
m [mg]	0.106	0.002	0.000	0.107
TON [g/(kg d)]	0.127	0.002	0.000	0.130

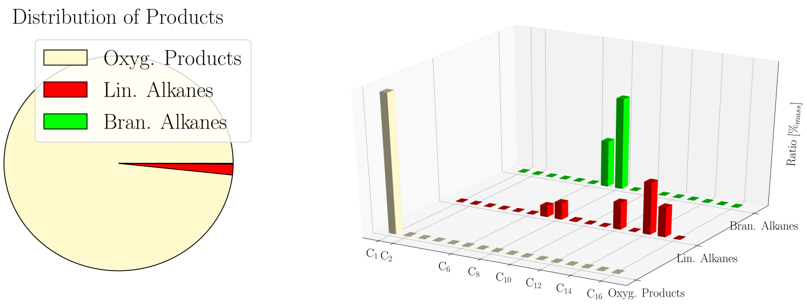

Figure S89. Distribution of products in the liquid phase.

Reaction 76: Campo del Cielo @ Montmorillonite

Table S163. Reaction conditions and masses of the entire catalysts (Cat) and its metal share (Met).

Synthesis	m(Cat) [g]	m(Met) [g]	t [d]	T [°C]	p(H_2) [bar]	p(CO_2) [bar]
Nano Particles	0.999	0.077	10	250	1.0	20.0

Table S164. Masses (m) and turnover numbers (TON) of the products: Oxygenated products (Oxy), alkanes (Alk), *iso*-alkanes (Iso), and their sum (Tot).

	Oxy	Alk	Iso	Tot
m [mg]	0.038	0.000	-0.000	0.038
TON [g/(kg d)]	0.050	0.000	0.000	0.050

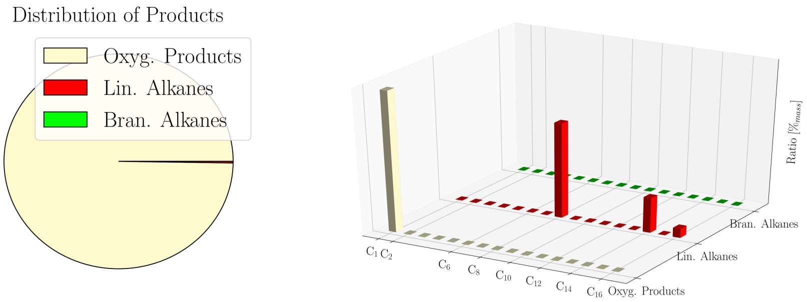

Figure S90. Distribution of products in the liquid phase.

Reaction 77: Campo del Cielo @ Montmorillonite

Table S165. Reaction conditions and masses of the entire catalysts (Cat) and its metal share (Met).

Synthesis	m(Cat) [g]	m(Met) [g]	t [d]	T [°C]	p(H_2) [bar]	p(CO_2) [bar]
Nano Particles	1.078	0.083	10	200	1.0	10.0

Table S166. Masses (m) and turnover numbers (TON) of the products: Oxygenated products (Oxy), alkanes (Alk), *iso*-alkanes (Iso), and their sum (Tot).

	Oxy	Alk	Iso	Tot
m [mg]	0.090	0.001	-0.000	0.090
TON [g/(kg d)]	0.108	0.001	-0.000	0.109

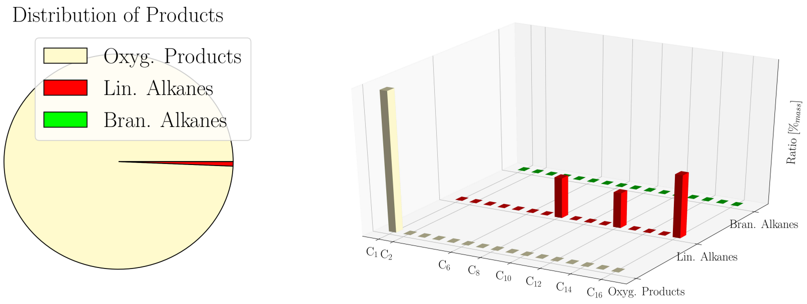

Figure S91. Distribution of products in the liquid phase.

Reaction 78: Campo del Cielo @ Montmorillonite

Table S167. Reaction conditions and masses of the entire catalysts (Cat) and its metal share (Met).

Synthesis	m(Cat) [g]	m(Met) [g]	t [d]	T [°C]	p(H_2) [bar]	p(CO_2) [bar]
Nano Particles	1.029	0.079	10	300	1.0	20.0

Table S168. Masses (m) and turnover numbers (TON) of the products: Oxygenated products (Oxy), alkanes (Alk), *iso*-alkanes (Iso), and their sum (Tot).

	Oxy	Alk	Iso	Tot
m [mg]	0.009	0.005	-0.000	0.013
TON [g/(kg d)]	0.011	0.006	0.000	0.017

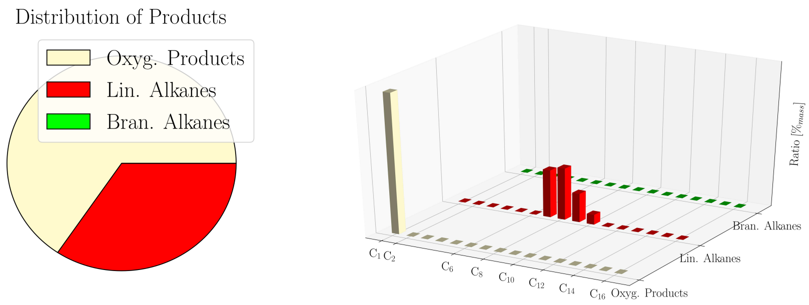

Figure S92. Distribution of products in the liquid phase.

Reaction 79: Campo del Cielo @ Montmorillonite

Table S169. Reaction conditions and masses of the entire catalysts (Cat) and its metal share (Met).

Synthesis	m(Cat) [g]	m(Met) [g]	t [d]	T [°C]	$p(H_2)$ [bar]	$p(CO_2)$ [bar]
Nano Particles	1.008	0.077	5	300	2.0	40.0

Table S170. Masses (m) and turnover numbers (TON) of the products: Oxygenated products (Oxy), alkanes (Alk), *iso*-alkanes (Iso), and their sum (Tot).

	Oxy	Alk	Iso	Tot
m [mg]	0.003	0.001	0.001	0.005
TON [g/(kg d)]	0.009	0.002	0.001	0.012

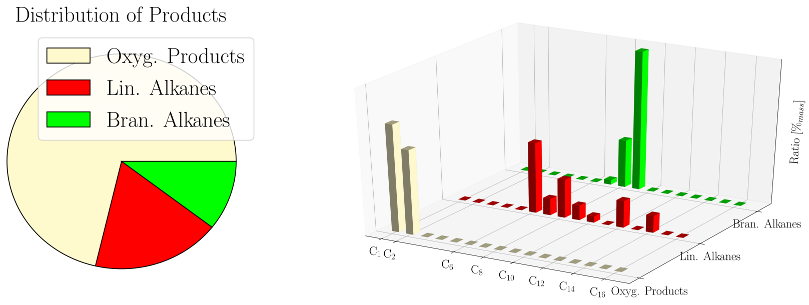

Figure S93. Distribution of products in the liquid phase.

Reaction 80: Campo del Cielo @ Montmorillonite

Table S171. Reaction conditions and masses of the entire catalysts (Cat) and its metal share (Met).

Synthesis	m(Cat) [g]	m(Met) [g]	t [d]	T [°C]	p(H_2) [bar]	p(CO_2) [bar]
Nano Particles	1.021	0.078	8	300	2.0	30.0

Table S172. Masses (m) and turnover numbers (TON) of the products: Oxygenated products (Oxy), alkanes (Alk), *iso*-alkanes (Iso), and their sum (Tot).

	Oxy	Alk	Iso	Tot
m [mg]	0.003	0.000	0.000	0.003
TON [g/(kg d)]	0.004	0.000	0.000	0.004

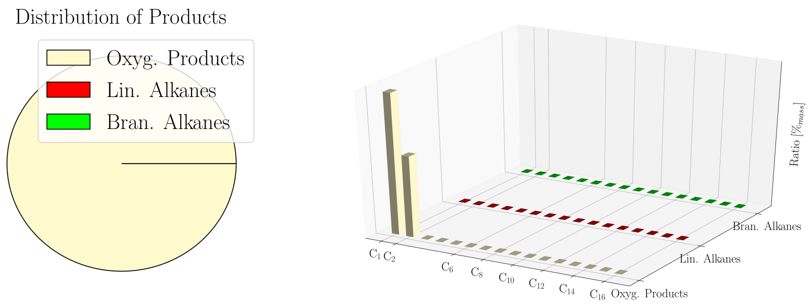

Figure S94. Distribution of products in the liquid phase.

Reaction 81: Campo del Cielo @ Montmorillonite

Table S173. Reaction conditions and masses of the entire catalysts (Cat) and its metal share (Met).

Synthesis	m(Cat) [g]	m(Met) [g]	t [d]	T [°C]	p(H_2) [bar]	p(CO_2) [bar]
Nano Particles	1.020	0.078	3	300	2.0	8.0

Table S174. Masses (m) and turnover numbers (TON) of the products: Oxygenated products (Oxy), alkanes (Alk), *iso*-alkanes (Iso), and their sum (Tot).

	Oxy	Alk	Iso	Tot
m [mg]	0.003	0.000	0.000	0.003
TON [g/(kg d)]	0.011	0.002	0.000	0.013

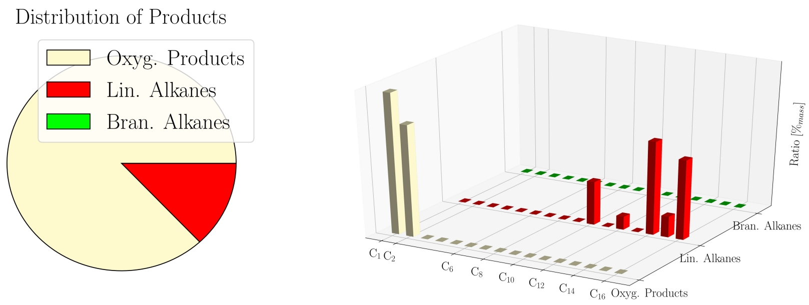

Figure S95. Distribution of products in the liquid phase.

[1] E. Hayek and W. Stadlmann, Angew. Chemie **67**, 327 (1955).

[2] J. M. Burlitch, M. L. Beeman, B. Riley, and D. L. Kohlstedt, Chem. Mater. **3**, 692 (1991).

[3] M. Siebert, G. Storch, and O. Trapp, Org. Process Res. Dev. **24**, 1304 (2020).

[4] R. L. Espinoza, S. Harding, J. Labuschagne, and A. P. Steynberg, Hydrogenation of Hydrocarbons **WO9837168A2** (1998-08-27).

[5] J. N. Connelly, Y. Amelin, A. N. Krot, and M. Bizzarro, ApJL **675**, L121 (2008).

[6] S. J. Mojzsis, "Earth, formation and early evolution," in *Encyclopedia of Astrobiology*, edited by M. Gargaud, R. Amils, J. C. Quintanilla, H. J. J. Cleaves, W. M. Irvine, D. L. Pinti, and M. Viso (Springer Berlin Heidelberg, Berlin, Heidelberg, 2011) pp. 467–474.

[7] G. Yu and S. B. Jacobsen, Proceedings of the National Academy of Sciences **108**, 17604 (2011), https://www.pnas.org/content/108/43/17604.full.pdf.

[8] H. Lammer, A. L. Zerkle, S. Gebauer, N. Tosi, L. Noack, M. Scherf, E. Pilat-Lohinger, M. Güdel, J. L. Grenfell, M. Godolt, and A. Nikolaou, Astron. Astrophys. Rev. **26**, 2 (2018).

[9] M. M. Thiemens, P. Sprung, R. O. C. Fonseca, F. P. Leitzke, and C. Münker, Nature Geoscience **12**, 696 (2019).

[10] S. J. Mojzsis, AGU Fall Meeting Abstracts , U52A-0009 (2001).

[11] L. Schaefer and B. Fegley, Icarus **208**, 438 (2010), arXiv:0909.4050 [astro-ph.EP].

[12] R. Heller, J.-P. Duda, M. Winkler, J. Reitner, and L. Gizon, arXiv e-prints , arXiv:2007.03423 (2020), arXiv:2007.03423 [astro-ph.EP].

[13] S. J. Mojzsis, T. M. Harrison, and R. T. Pidgeon, Nature **409**, 178 (2001).

[14] S. A. Wilde, J. W. Valley, W. H. Peck, and C. M. Graham, Nature **409**, 175 (2001).

[15] J. Kasting, Science **259**, 920 (1993).

[16] J. F. Kasting and S. Ono, Philos. Trans. R. Soc. Lond., B., Biol. Sci. **361**, 917 (2006).

[17] L. Schaefer and B. Fegley, Icarus **186**, 462 (2007).

[18] G. H. Shaw, Chem. Erde **68**, 235 (2008).

[19] K. Zahnle, L. Schaefer, and B. Fegley, Cold Spring Harbor Perspectives in Biology **2** (2010), 10.1101/cshperspect.a004895, http://cshperspectives.cshlp.org/content/2/10/a004895.full.pdf+html.

[20] K. J. Zahnle, D. C. Catling, and M. W. Claire, Chemical Geology **362**, 26 (2013), special Issue dedicated to H.D. Holland: Evolution of the atmosphere and ocean through time.

[21] K. J. Zahnle, R. Lupu, D. C. Catling, and N. Wogan, The Planetary Science Journal **1**, 11 (2020).

[22] C. Bounama, S. Franck, and W. von Bloh, Hydrology and Earth System Sciences **5**, 569 (2001).

[23] J. L. Bada and J. Korenaga, Life **8** (2018), 10.3390/life8040055.

[24] B. K. D. Pearce, R. E. Pudritz, D. A. Semenov, and T. K. Henning, Proc. Natl. Acad. Sci. U.S.A. **114**, 11327 (2017).

[25] K. Takai, K. Nakamura, T. Toki, U. Tsunogai, M. Miyazaki, J. Miyazaki, H. Hirayama, S. Nakagawa, T. Nunoura, and K. Horikoshi, Proc Natl Acad Sci U S A **105**, 10949 (2008).

[26] R. Gomes, H. F. Levison, K. Tsiganis, and A. Morbidelli, Nature **435**, 466 (2005).

[27] P. Claeys and A. Morbidelli, "Late heavy bombardment," in *Encyclopedia of Astrobiology*, edited by M. Gargaud, R. Amils, J. C. Quintanilla, H. J. J. Cleaves, W. M. Irvine, D. L. Pinti, and M. Viso (Springer Berlin Heidelberg, Berlin, Heidelberg, 2011) pp. 909–912.

[28] W. F. Bottke, D. Vokrouhlický, D. Minton, D. Nesvorny, A. Morbidelli, R. Brasser, B. Simonson, and H. F. Levison, Nature **485**, 78 (2012).

[29] O. Abramov, D. A. Kring, and S. J. Mojzsis, Chem. Erde **73**, 227 (2013).

[30] S. Marchi, W. F. Bottke, L. T. Elkins-Tanton, M. Bierhaus, K. Wuennemann, A. Morbidelli, and D. A. Kring, Nature **511**, 578 EP (2014).

[31] S. J. Mojzsis, R. Brasser, N. M. Kelly, O. Abramov, and S. C. Werner, The Astrophysical Journal **881**, 44 (2019).

[32] R. G. Strom, R. Malhotra, T. Ito, F. Yoshida, and D. A. Kring, Science **309**, 1847 (2005), arXiv:astro-ph/0510200 [astro-ph].

[33] C. Koeberl, W. U. Reimold, and R. L. Gibson, "The record of impact processes on the early earth: A review of the first 2.5 billion years," in *Processes on the Early Earth* (Geological Society of America, 2006).

[34] A. Morbidelli, W. F. Bottke, D. Nesvorný, and H. F. Levison, Icarus **204**, 558 (2009), arXiv:0907.2512 [astro-ph.EP].

[35] B. Marty, K. Altwegg, H. Balsiger, A. Bar-Nun, D. V. Bekaert, J.-J. Berthelier, A. Bieler, C. Briois, U. Calmonte, M. Combi, J. De Keyser, B. Fiethe, S. A. Fuselier, S. Gasc, T. I. Gombosi, K. C. Hansen, M. Hässig, A. Jäckel, E. Kopp, A. Korth, L. Le Roy, U. Mall, O. Mousis, T. Owen, H. Rème, M. Rubin, T. Sémon, C.-Y. Tzou, J. H. Waite, and P. Wurz, Science **356**, 1069 (2017), https://science.sciencemag.org/content/356/6342/1069.full.pdf.

[36] J. L. Bada, Chem. Soc. Rev. **42**, 2186 (2013).

[37] N. MOSKOVITZ and E. GAIDOS, Meteoritics & Planetary Science **46**, 903–918 (2011).

[38] W. F. Bottke, D. Nesvorný, R. E. Grimm, A. Morbidelli, and D. P. O'Brien, Nature **439**, 821 (2006).

[39] T. S. Kruijer, M. Touboul, M. Fischer-Gödde, K. R. Bermingham, R. J. Walker, and T. Kleine, Science **344**, 1150 (2014), https://science.sciencemag.org/content/344/6188/1150.full.pdf.

[40] J. L. Margot and M. E. Brown, Science **300**, 1939 (2003).

[41] H. P. Gail, M. Trieloff, D. Breuer, and T. Spohn, in *Protostars and Planets VI*, edited by H. Beuther, R. S. Klessen, C. P. Dullemond, and T. Henning (2014) p. 571, arXiv:1312.3509 [astro-ph.EP].

[42] H. Palme, Reviews in Modern Astronomy **1**, 28 (1988).

[43] P. Agrawal, P. M. Jenniskens, E. Stern, J. Arnold, and Y.-K. Chen, "Arcjet ablation of stony and iron meteorites," in *2018 Aerodynamic Measurement Technology and Ground Testing Conference* (2018) https://arc.aiaa.org/doi/pdf/10.2514/6.2018-4284.

[44] E. M. van Kooten, E. Kubik, J. Siebert, B. D. Heredia, T. B. Thomsen, and F. Moynier, Geochimica et Cosmochimica Acta **321**, 52 (2022).

[45] W. F. Bottke, D. D. Durda, D. Nesvorný, R. Jedicke, A. Morbidelli, D. Vokrouhlický, and H. Levison, Icarus **175**, 111 (2005).

[46] S. Marchi, B. Black, L. Elkins-Tanton, and W. Bottke, Earth and Planetary Science Letters **449**, 96 (2016).

[47] N. H. Sleep and K. Zahnle, Journal of Geophysical Research: Planets **103**, 28529 (1998).

[48] O. Abramov and S. J. Mojzsis, Nature **459**, 419 (2009).

[49] D. de Niem, E. Kührt, A. Morbidelli, and U. Motschmann, Icarus **221**, 495 (2012).

[50] H. E. Schlichting, R. Sari, and A. Yalinewich, Icarus **247**, 81 (2015).

[51] C. A. Sinclair, M. C. Wyatt, A. Morbidelli, and D. Nesvorný, Mon. Not. R. Astron. Soc **499**, 5334 (2020), arXiv:2010.06254 [astro-ph.EP].

[52] C. Chyba and C. Sagan, Nature **355**, 125 (1992).

[53] A. D. Anbar, K. J. Zahnle, G. L. Arnold, and S. J. Mojzsis, J. Geophys. Res. **106**, 3219 (2001).

[54] U. G. Jørgensen, P. W. Appel, Y. Hatsukawa, R. Frei, M. Oshima, Y. Toh, and A. Kimura, Icarus **204**, 368 (2009).

[55] H. Krüger and E. Grün, in *Encyclopedia of the Solar System (Third Edition)*, edited by T. Spohn, D. Breuer, and T. V. Johnson (Elsevier, Boston, 2014) third edition ed., pp. 657–682.

[56] C. Mehta, A. Perez, G. Thompson, and M. A. Pasek, Life (Basel, Switzerland) **8**, 13 (2018).

[57] J. Lovering, L. Parry, and J. Jaeger, Geochimica et Cosmochimica Acta **19**, 156 (1960).

[58] Z. Li, R. Caracas, and F. Soubiran, Earth and Planetary Science Letters **547**, 116463 (2020).

[59] Y. Sekine, S. Sugita, T. Kadono, and T. Matsui, Journal of Geophysical Research: Planets **108** (2003), https://doi.org/10.1029/2002JE002034, https://agupubs.onlinelibrary.wiley.com/doi/pdf/10.1029/2002JE002034.

[60] M. E. Kress and C. P. McKay, Icarus **168**, 475 (2004).

[61] A. Giesen, J. Herzler, and P. Roth, Phys. Chem. Chem. Phys. **4**, 3665 (2002).

[62] R. Ferrante, M. Moore, J. Nuth, and T. Smith, Icarus **145**, 297 (2000).

[63] D. R. Mole, M. L. Fiorentini, N. Thebaud, K. F. Cassidy, T. C. McCuaig, C. L. Kirkland, S. S. Romano, M. P. Doublier, E. A. Belousova, S. J. Barnes, and J. Miller, Proceedings of the National Academy of Sciences **111**, 10083 (2014), https://www.pnas.org/content/111/28/10083.full.pdf.

[64] B. Dhuime, C. J. Hawkesworth, P. A. Cawood, and C. D. Storey, Science **335**, 1334 (2012).

[65] J. Korenaga, Annual Review of Earth and Planetary Sciences **41**, 117 (2013).

[66] H. Gamal El Dien, L. S. Doucet, J. B. Murphy, and Z.-X. Li, Scientific Reports **10**, 9461 (2020).

[67] M. Hopkins, T. M. Harrison, and C. E. Manning, Nature **456**, 493 (2008).

[68] A. Prokoph, R. E. Ernst, and K. L. Buchan, The Journal of Geology **112**, 1 (2004), https://doi.org/10.1086/379689.

[69] N. T. Arndt and E. G. Nisbet, Annual Review of Earth and Planetary Sciences **40**, 521 (2012), https://doi.org/10.1146/annurev-earth-042711-105316.

[70] S. D. King and C. Adam, Physics of the Earth and Planetary Interiors **235**, 66 (2014).

[71] B. Damer and D. Deamer, Life **5**, 872 (2015).

[72] E. Nisbet, M. Cheadle, N. Arndt, and M. Bickle, Lithos **30**, 291 (1993), the evolving earth.

[73] T. Grove and S. Parman, Earth and Planetary Science Letters **219**, 173 (2004).

[74] B. McConnell, Geological Journal **46**, 109 (2011), https://onlinelibrary.wiley.com/doi/pdf/10.1002/gj.1240.

[75] T. J. Jones and J. K. Russell, Scientific Reports **7**, 5538 (2017).

[76] E. Tagaris, A. M. Siani, and B. Langmann, ISRN Atmospheric Sciences **2013**, 245076 (2013).

[77] P. Wignall, Earth-Science Reviews **53**, 1 (2001).

[78] G. Feulner, Reviews of Geophysics **50** (2012), 10.1029/2011RG000375, https://agupubs.onlinelibrary.wiley.com/doi/pdf/10.1029/2011RG000375.

[79] S. Ranjan, Z. R. Todd, J. D. Sutherland, and D. D. Sasselov, Astrobiology **18**, 1023 (2018).

[80] C. Herzberg, K. Condie, and J. Korenaga, Earth and Planetary Science Letters **292**, 79 (2010).

[81] B. Langmann, ISRN Atmospheric Sciences **2013**, 17 (2013).

[82] A. Vogel, S. Diplas, A. J. Durant, A. S. Azar, M. F. Sunding, W. I. Rose, A. Sytchkova, C. Bonadonna, K. Krüger, and A. Stohl, *Journal of Geophysical Research: Atmospheres*, Journal of Geophysical Research: Atmospheres **122**, 9485 (2017).

[83] B. Esse, M. Burton, M. Varnam, R. Kazahaya, P. A. Wallace, F. Von-Aulock, Y. Lavallée, G. Salerno, S. Scollo, and H. Coe, Scientific Reports **8**, 15680 (2018).

[84] I. Manzella, C. Bonadonna, J. C. Phillips, and H. Monnard, Geology **43**, 211 (2015).

[85] C. Bonadonna, G. Ernst, and R. Sparks, Journal of Volcanology and Geothermal Research **81**, 173 (1998).

[86] A. J. Durant, Geology **43**, 271 (2015).

[87] M. Gouhier, J. Eychenne, N. Azzaoui, A. Guillin, M. Deslandes, M. Poret, A. Costa, and P. Husson, Scientific Reports **9**, 1449 (2019).

[88] L. Pioli, C. Bonadonna, and M. Pistolesi, Scientific Reports **9**, 10006 (2019).

[89] K. Zahnle, N. Arndt, C. Cockell, A. Halliday, E. Nisbet, F. Selsis, and N. H. Sleep, Space

Science Reviews **129**, 35 (2007).

[90] N. H. Sleep, Cold Spring Harb Perspect Biol. **2**, 10.1101/cshperspect.a002527.

[91] H. James Cleaves II, A. Michalkova Scott, F. C. Hill, J. Leszczynski, N. Sahai, and R. Hazen, Chem. Soc. Rev. **41**, 5502 (2012).

[92] H. Bu, P. Yuan, H. Liu, D. Liu, Z. Qin, X. Zhong, H. Song, and Y. Li, Chemical Geology **510**, 72 (2019).

[93] J. T. T. Kloprogge and H. Hartman, Life **12** (2022), 10.3390/life12020259.
